# Sparse high-dimensional decomposition of non-primary auditory cortical receptive fields

**DOI:** 10.1371/journal.pcbi.1012721

**Published:** 2025-01-02

**Authors:** Shoutik Mukherjee, Behtash Babadi, Shihab Shamma

**Affiliations:** 1 Department of Electrical and Computer Engineering, University of Maryland, College Park, Maryland, United States of America; 2 Institute for Systems Research, University of Maryland, College Park, Maryland, United States of America; 3 Laboratoire des Systèmes Perceptifs, Department des Études Cognitive, École Normale Supériure, Paris Sciences et Lettres University, Paris, France; Royal Institute of Technology (KTH), SWEDEN

## Abstract

Characterizing neuronal responses to natural stimuli remains a central goal in sensory neuroscience. In auditory cortical neurons, the stimulus selectivity of elicited spiking activity is summarized by a spectrotemporal receptive field (STRF) that relates neuronal responses to the stimulus spectrogram. Though effective in characterizing primary auditory cortical responses, STRFs of non-primary auditory neurons can be quite intricate, reflecting their mixed selectivity. The complexity of non-primary STRFs hence impedes understanding how acoustic stimulus representations are transformed along the auditory pathway. Here, we focus on the relationship between ferret primary auditory cortex (A1) and a secondary region, dorsal posterior ectosylvian gyrus (PEG). We propose estimating receptive fields in PEG with respect to a well-established high-dimensional computational model of primary-cortical stimulus representations. These “cortical receptive fields” (CortRF) are estimated greedily to identify the salient primary-cortical features modulating spiking responses and in turn related to corresponding spectrotemporal features. Hence, they provide biologically plausible hierarchical decompositions of STRFs in PEG. Such CortRF analysis was applied to PEG neuronal responses to speech and temporally orthogonal ripple combination (TORC) stimuli and, for comparison, to A1 neuronal responses. CortRFs of PEG neurons captured their selectivity to more complex spectrotemporal features than A1 neurons; moreover, CortRF models were more predictive of PEG (but not A1) responses to speech. Our results thus suggest that secondary-cortical stimulus representations can be computed as sparse combinations of primary-cortical features that facilitate encoding natural stimuli. Thus, by adding the primary-cortical representation, we can account for PEG single-unit responses to natural sounds better than bypassing it and considering as input the auditory spectrogram. These results confirm with explicit details the presumed hierarchical organization of the auditory cortex.

## Author summary

Spectrotemporal receptive fields (STRF) summarize how auditory neurons respond to the time-lagged frequency content of acoustic stimuli. However, in non-primary auditory cortex, where neurons can be sensitive to a wide range of spectrotemporal features, complex STRFs pose difficulty in understanding how stimulus representations change along the auditory path. In this study, we focus on relating ferret primary auditory cortex (A1) to a secondary area known as the posterior ectosylvian gyrus (PEG). We propose a methodology in which we model PEG responses with respect to a well-established computational model of the earlier primary cortical stage (A1), thus estimating a “cortical receptive field” (CortRF). We demonstrate the utility of CortRF analysis in application to single-unit recordings of PEG and A1 spiking responses to speech and artificial frequency-modulated noise stimuli. CortRFs of PEG neurons were found to capture their selectivity to more complex spectrotemporal features than A1 neurons; moreover, CortRF models were more predictive of PEG responses to speech. Consistent with previous hypotheses about hierarchical organization in auditory cortex, our results show that adding the primary-cortical representation accounts for PEG single-unit responses to natural sounds better than otherwise and indicate that PEG neurons encode natural stimuli better than earlier areas.

## Introduction

Animals must be able to parse complex sensory inputs in order to navigate their environments. In relation to audition, this includes scene analysis, semantic processing, and vocal learning [1–5]. Neural representations of the acoustic environment must be sufficiently rich to support such higher-order processing, and hence characterizing the acoustic stimulus representations encoded by neuronal responses remains a central goal in auditory neuroscience. Receptive field estimation continues to be a widely used approach to characterize single-neuron level stimulus representations in all sensory systems, especially auditory and visual systems [6–13]. The receptive field of a neuron describes its feature selectivity, or equally, a stimulus transformation that the neuron represents. Hence, the receptive fields of a neuronal population describes how stimuli are represented by it, as shared receptive field properties elucidate the nature of the stimulus transformation the population’s activity represents.

Conventionally, receptive fields are estimated by reverse-correlating the measured neuronal responses to a diverse range of stimuli with features of the stimuli, assuming a linear relationship with additive Gaussian observation noise [6, 7, 11, 14–16]. However, if insufficiently diverse stimuli are used, or if stimulus presentations are too few to obtain smooth peristimulus time histograms (PSTH), an unconstrained linear-Gaussian response model is susceptible to inaccurately characterizing the receptive fields. These challenges have motivated the use of sparse or smooth receptive field priors (especially when using responses to natural stimuli) [17–20] and generalized linear models (GLM) for neuronal spiking activity [10, 21–24]; individually and jointly, both assumptions have been shown to improve the accuracy and generalizability of the receptive field. Low-dimensional factorizations [9, 25] have also been proposed to obtain tractable and generalizable receptive field models. Notably, these approaches all utilize early-stage stimulus representations to estimate the receptive fields irrespective of the cortical area of interest. Specifically, auditory cortical neurons are typically characterized by *spectrotemporal* receptive fields (STRFs) that act on spectrogram inputs—the neural representations of acoustic signals at the earliest stages.

Such descriptions of response selectivity with respect to simple stimulus features can provide intuitive interpretations of how stimuli are encoded by cortical neurons. However, receptive fields estimated in non-primary areas are often highly complex, reflecting the mixed selectivity that neurons in higher-order areas exhibit [9, 26–30]. Consequently, studies of higher sensory processing have sought to characterize and investigate the utility of hierarchical stimulus representations [3, 31–38]. The advent of deep learning has inspired computational models for higher-order areas that implement feed-forward neural network architectures. For example, convolutional neural networks have been utilized to directly model neuronal responses in higher-order visual areas [37, 39–41], and similarly more recent studies have applied feed-forward neural network models to the auditory system [32, 42–45]. Estimating deep convolutional models essentially characterizes receptive fields of each intermediate layer with respect to embedded features produced through the composition of hierarchical stimulus transformations over the preceding layers in the pursuit of interpretable decompositions of complex features. Such models have been demonstrated post hoc to accurately characterize the neural responses of higher-order visual [39, 40] neurons and non-primary auditory areas [32, 42] in deeper network layers, with several intermediate network layers resembling primary cortical responses. While neural network models describe hierarchical schemes by which higher-order sensory areas encode complex stimulus representations, they do not explicitly characterize hierarchical computations underlying the neuronal responses along the sensory pathway. Therefore, to this end, we sought to model directly how non-primary neuronal responses are related to primary-cortical stimulus representations.

With a focus on the mammalian auditory system and using a ferret animal model, we demonstrate here an approach to estimating the receptive fields of neurons in secondary auditory cortex utilizing a well-established computational model of acoustic signal representations in primary auditory cortex (A1) [33, 46, 47]. The spiking responses of neurons in ferret dorsal posterior ectosylvian gyrus (PEG), a secondary auditory area with direct inputs from A1, are modeled by GLMs that use primary-cortical features obtained via multiresolution analysis of the spectrogram as regressors [46]. The proposed model is conceptually similar to neural network models in that a hierarchical stimulus representation is utilized to decompose complex STRFs. However, since the stimulus regressors represent the neural responses in primary auditory cortex (A1), the cortical receptive fields (CortRF) estimated in this proposed approach directly describe the computations by PEG neurons involving primary-cortical features that produce a secondary stimulus representation. Noting that the multiresolution primary-cortical features are high-dimensional and that sparse priors in STRF estimation improve model generalizability [9, 17, 24], we also impose sparsity constraints on the estimated CortRFs by using orthogonal matching pursuit (OMP) [48, 49] over an overcomplete dictionary spanning the primary-cortical feature space. We demonstrate that the proposed receptive field analysis can recover the true stimulus dependence of simulated spiking activity generated in response to a wide range of stimuli. In its application to PEG neurons, we show that estimated CortRFs are predictive of spiking responses, specifically outperforming STRF-based predictions of unseen responses to speech. We additionally analyze neurons in A1 and find that not only is the predictive advantage absent, but that substantial differences in receptive field properties emerge between PEG and A1. Thus, this proposed method provides new insights into the hierarchical stimulus representations in the mammalian auditory system.

## Results

In order to study how responses of neurons in non-primary auditory areas encode features of acoustic stimuli, we analyzed the receptive fields of neurons in ferret posterior ectosylvian gyri (PEG) recorded passively during the presentation of artificial and natural acoustic stimuli. Specifically, we propose a new approach to receptive field estimation in non-primary auditory neurons that, by leveraging the representation of acoustic stimuli in primary auditory cortex (A1), can explain how complex spectrotemporal selectivity arises downstream.

**Fig 1:**
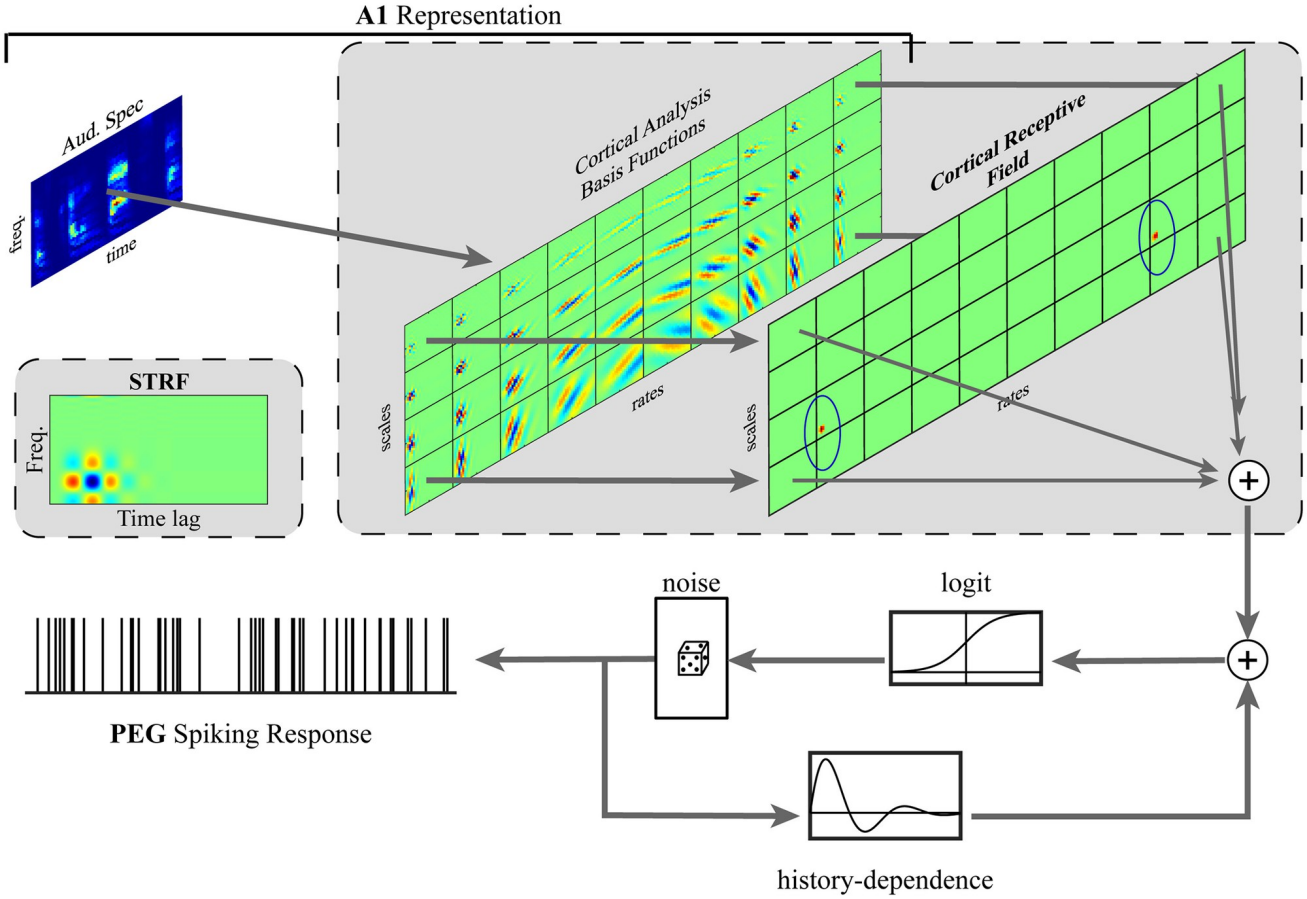
Cortical receptive fields of PEG neurons are estimated with respect to the primary-cortical representation of acoustic stimuli by fitting generalized linear models (GLM) to spiking responses. Each scale-rate channel of the primary-cortical representation, obtained by the convolving the stimulus auditory spectrogram by the associated basis function, is convolved with the corresponding channel of the cortical receptive field. The outputs of each scale-rate channel linearly combine to modulate the conditional intensity function (CIF), which is logistically linked to stimulus and spiking history modulations. The equivalent spectrotemporal receptive field (STRF) is thus computed as the linear combination of the spectrotemporal filters of each scale-rate channel.

The proposed approach, illustrated in Fig 1, treats the spiking responses of PEG neurons as point processes whose conditional intensity functions (CIF) are modeled by generalized linear models (GLM) [21] with both stimulus and spiking history regressors. While conventionally the spectrogram over a specified integration window would be used as stimulus regressors to estimate a spectrotemporal receptive field (STRF), we instead utilize the representation of acoustic stimuli in primary auditory cortex [46]. This primary-cortical representation is obtained by a 2-D multiresolution analysis of the spectrogram defined by a set of basis functions selective for different bandwidths (i.e. scales) and for different modulation rates, examples of which are shown in Fig 1.

Just as STRFs are estimated with respect to spectrotemporal features, we estimate the “cortical receptive fields” (CortRF) of neurons with respect to these primary-cortical features. However, in consideration of the high dimensionality of the feature space and based on previous studies that demonstrate the generalizability of sparsely estimated receptive fields [9, 17, 24], we utilized a generalized Orthogonal Matching Pursuit (OMP) algorithm over a dictionary of Gaussian atoms spanning the primary-cortical feature space to impose sparse priors when estimating CortRFs. OMP iteratively identifies a support set—the non-zero subset of parameters—over which the model is optimized. In the context of the proposed model, OMP iteratively identifies atoms of the dictionary and estimates their weights in order to obtain the CortRF. Noting that the proposed system model is linear with respect to the spectrogram, CortRFs have equivalent STRFs that are obtained by convolving the CortRF’s scale-rate channels by the appropriate primary-cortical basis functions and computing the sum over all scale-rate channels. To distinguish STRFs computed in this manner from those estimated directly, we denote them as CortSTRFs.

The following results validate the proposed hierarchical model of feature selectivity in PEG neurons and indicate that PEG neurons likely capture encoding of natural stimuli better than earlier areas. First, we simulated the spiking responses of a neuron with a known receptive field in order to demonstrate the efficacy of our proposed methods in recovering the receptive field and estimating a useful generative model. Then, we applied the proposed analyses to PEG neurons recorded from ferrets during the passive presentation of natural (specifically, speech) and artificial (temporally orthogonal ripple combinations, or TORCs [15]) stimuli. We additionally compared how well CortRF models vs. directly estimated STRF models of PEG neurons were able to predict the spiking responses to unseen speech and TORC stimuli, and found that the CortRF enabled significantly better predictions of responses to natural stimuli. We sought to determine if this benefit was unique to PEG neurons and hence applied the same CortRF and STRF analyses to neurons recorded in ferret A1, where we found no improvement in response predictions. Hypothesizing that CortRF analysis describes more complex spectrotemporal selectivity in PEG, we compared the complexity of the CortSTRFs and STRFs of A1 and PEG neurons. Finally, we performed a clustering analysis of the CortRFs to gain further insight into the auditory features that distinguished PEG neurons from A1 neurons.

### CortRF model of simulated neuron recovers true receptive field

**Fig 2:**
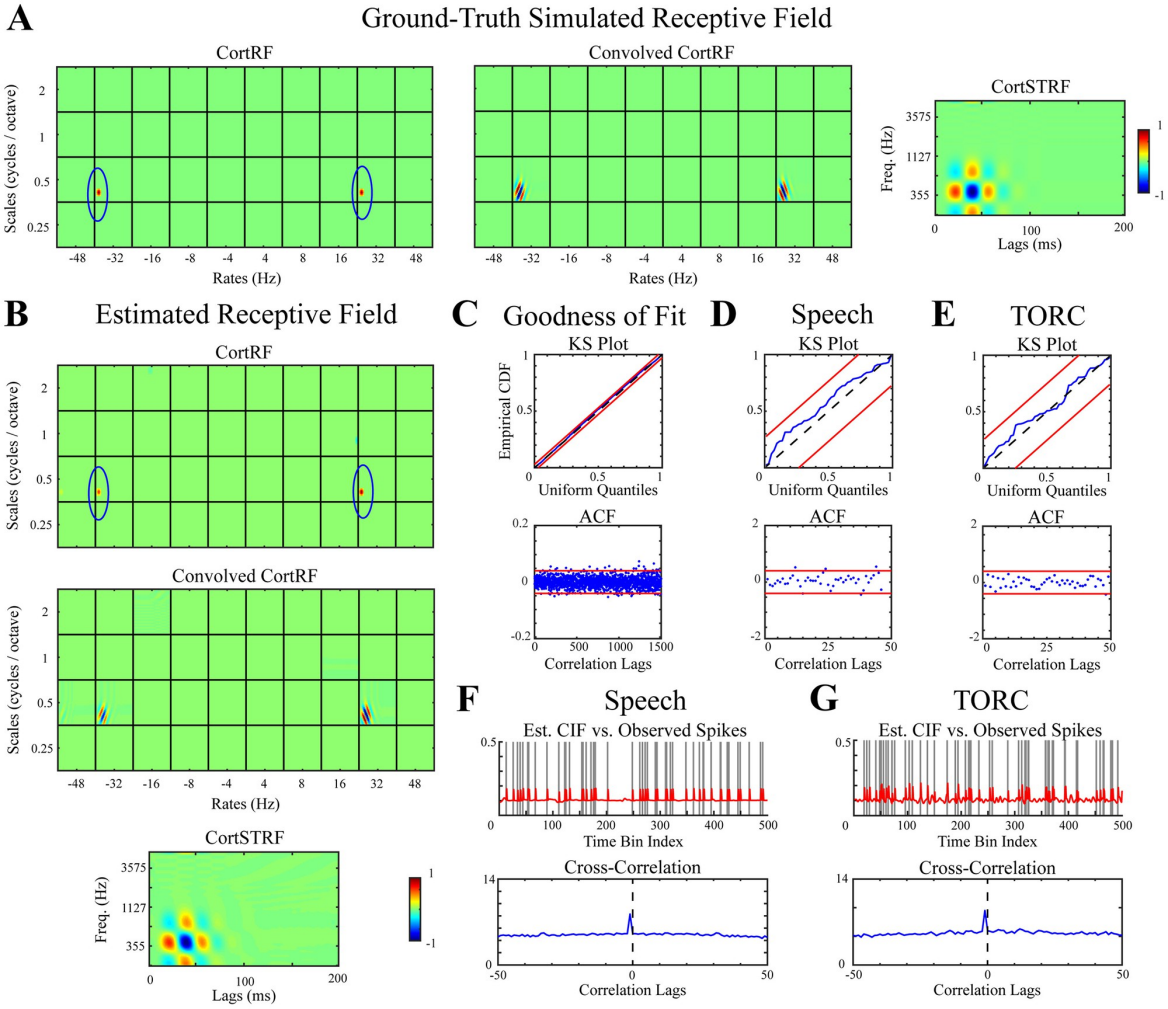
CortRF analysis of a simulated neuron. **A.** Ground-truth receptive field of simulated PEG neuron consisted of two components (circled in blue). **B.** Estimated cortical receptive field nearly exactly recovered ground-truth receptive field. The dominant features are circled in blue. **C.** Kolmogorov-Smirnov (KS) and autocorrelation function (ACF) tests of model goodness-of-fit with 95% confidence intervals show the history-dependent GLM accounted for simulated spiking statistics accurately. **D–E.** Single-realization goodness-of-fit tests showed that spiking responses of individual simulated realizations in response to both speech and TORC stimuli were well-modeled by the GLM. **F–G.** Estimated CIF vs. observed spiking. The estimated CIFs for unseen realizations of the simulated spiking process closely matched the spiking responses, with distinctive correlogram peaks close to 0-lag. Receptive fields have been normalized for visualization.

We first demonstrated the efficacy of CortRF estimation through simulation. The spiking activity of a simulated neuron was generated in response to a set of 30 TORCs and 30 sentences from the TIMIT corpus, with six realizations (i.e. “trials”) per stimulus. These stimuli were a subset of those presented to ferrets during *in vivo* recordings. The simulated neuron’s spiking history dependence was set to be self-exciting in order to mimic bursts of spiking activity observed in recorded neurons. The ground-truth CortRF consisted of two positively-weighted atoms located at the same scale-frequency channel and same time lag, but opposite rate channels. The true CortRF, the CortRF convolved by the primary-cortical basis functions, and the CortSTRF of the simulated neuron are shown in Fig 2A.

The CortRF was estimated by fitting a point process GLM to the simulated spiking responses to 24 TORCs and 24 sentences by applying OMP over an overcomplete dictionary of truncated Gaussian atoms spanning each scale-rate channel of the primary-cortical features (see Methods). The estimated CortRF nearly exactly recovered the ground-truth CortRF (Fig 2B); that is, the salient atoms in the greedily estimated CortRF (circled in blue) coincide with the true CortRF. Consequently, the convolved CortRF and CortSTRF closely match the ground truth.

We used the simulated responses to 6 TORCs and 6 sentences that were excluded during estimation to quantify the model’s goodness-of-fit. Invoking the time-rescaling theorem for point processes [50], we tested if the empirical distribution of time-rescaled interspike intervals in test responses matched a uniform distribution and if the interspike intervals were significantly correlated using graphical Kolmogorov-Smirnov (KS) and autocorrelation function (ACF) tests, respectively. The results of this evaluation over the entire test set, shown in Fig 2C with 95% confidence intervals, indicate that the estimated model accounted for the spiking statistics of unseen responses with confidence. We found this to be true of individual realizations of the simulation driven by speech and TORC presentations, as shown in Fig 2D (respectively, Fig 2E). Noting that the estimated models include spiking history regressors as well as the stimulus, the contribution of the former is evaluated in S1 Text; while important, spiking history is not sufficient to obtain models with good statistical fits to spiking observations.

We additionally evaluated how well the estimated model predicted spiking responses to stimuli excluded during estimation. For each realization, the predicted CIF was computed using the estimated GLM and cross-correlated with the observed spiking responses. Examples of the predicted CIFs and the observed spiking responses to a TORC and a sentence are showng in Fig 2F and 2G, respectively. Predicted CIFs closely matched the simulated spiking responses; correlograms had distinct peaks close to 0-lag. Specifically, predicted CIFs lagged behind simulated spiking by 1 sample due to the group delay of the causally estimated moving average filter parameterized by the spiking history modulation coefficients. Moreover, we computed the lag-corrected cosine similarities between the observed spiking and the predicted CIFs; a value of 1 indicates that both coincide exactly while a value of 0 indicates orthogonality. For speech responses, the median cosine similarity of all test speech responses was 0.6526 and was 0.6579 for test TORC responses. Thus, the results of our simulation indicate that sparse CortRF estimation using a point process model provides a descriptive and predictive model of spiking responses to acoustic stimuli.

### CortRFs facilitate predictions of speech responses in PEG neurons

#### PEG neuronal responses are well-characterized by CortRF point process models

We next applied CortRF analysis to the spiking responses of neurons recorded from ferret PEG during behaviorally passive presentation of TORC and speech stimuli. A total of 31 PEG neurons recorded across three animals were used in these analyses. Animals were presented with stimulus sets consisting of 30 TORC samples and 30 sentences from the TIMIT corpus that were repeated between 4 − 6 times in randomized orders (see Methods). The spiking responses to 24 TORCs and 24 sentences were used for estimation, while the remaining responses were used for model validation.

**Fig 3:**
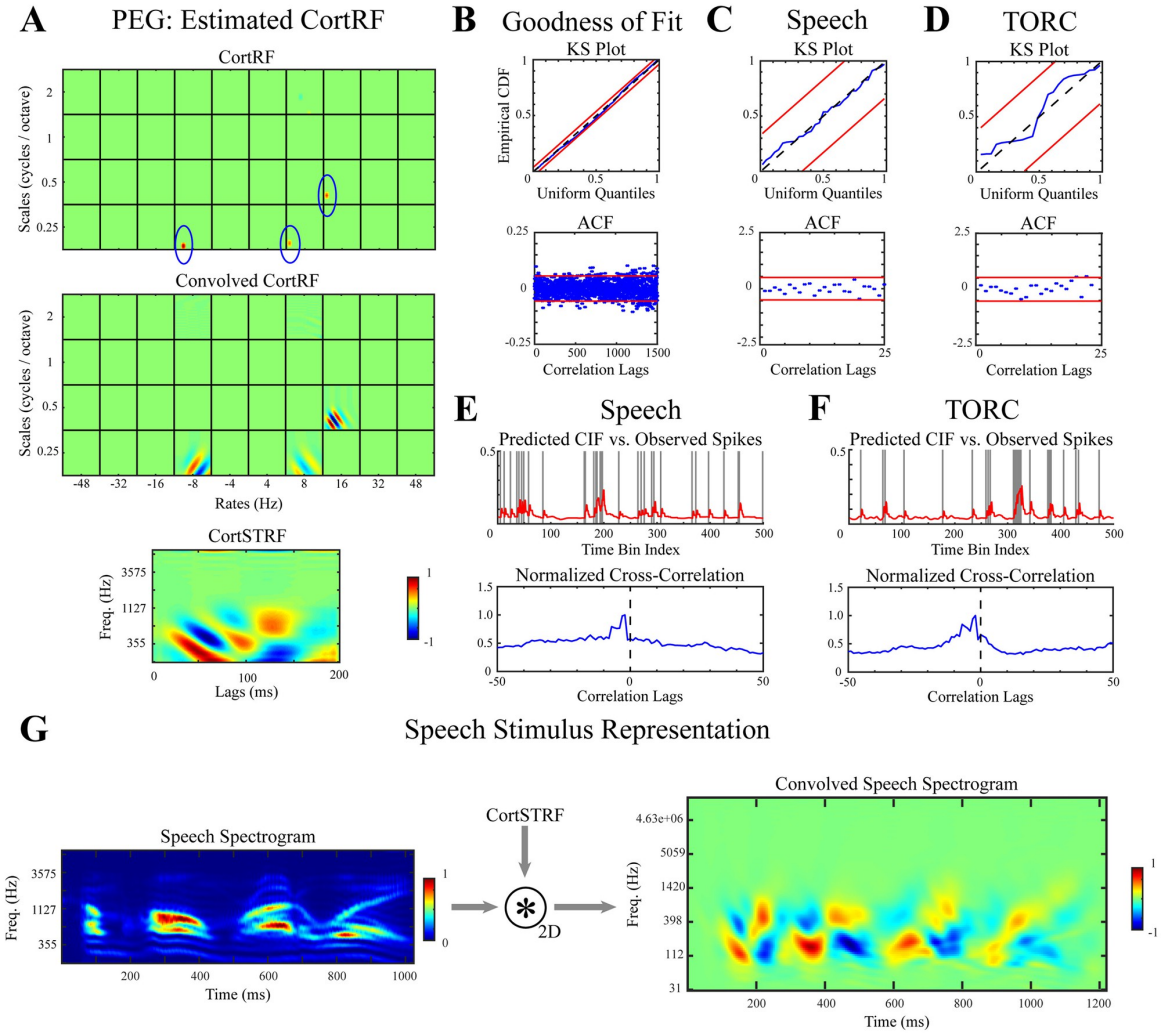
CortRF analysis of an example PEG neuron. **A.** The estimated CortRFs of PEG neurons were sparse, but produced complex CortSTRFs. Here, three atoms from the dictionary of primary-cortical features had non-zero weight, but correspond to a CortSTRF with wide temporal tuning **B.** KS and ACF tests show spiking statistics were well-matched by the estimated model over all speech and TORC stimulus repetitions withheld during model estimation. **C–D.** Single-trial spiking statistics were also well-matched. **E–F.** Comparing the predicted conditional intensity function (CIF) to withheld observed spiking responses showed the estimated model was highly predictive of spiking responses. **G.** The 2-dimensional convolution of a sample speech spectrogram with the estimated CortSTRF shows how the sentence would be represented by a family of PEG neurons with similar receptive fields translated in frequency. Spectrograms and receptive fields are normalized for visualization.

The CortRFs of PEG neurons were estimated using OMP over an overcomplete dictionary covering the primary-cortical feature space. Estimated CortRFs individually consisted of a sparse number of atoms (Fig 3A), though the atoms selected over all neurons were distributed across all scale-rate-frequency channels and time lags (S1 Fig). In order to visualize the spectrotemporal features that a CortRF described, the corresponding CortSTRF was computed by first convolving the CortRF with the primary-cortical basis function and then marginalizing over rates and scales (Fig 3A and S1 Fig). CortSTRFs demonstrated that PEG neurons were selective of complex spectrotemporal features that had sparse representations in the primary-cortical feature space.

In the absence of ground truth receptive fields to assess the accuracy of estimated CortRFs, we evaluated the goodness-of-fit of estimated models. The KS and ACF tests were applied to determine whether the distribution of time-rescaled interspike intervals in unseen responses were uniformly distributed and independent, respectively. The KS and ACF tests were applied both collectively and individually to each trial in the test set. Examples of KS and ACF tests over the entire test set, for one speech trial, and for one TORC trial are shown in Fig 3B–3D, respectively. Graphical KS and ACF tests for all PEG neurons are included in S1 Fig. We found that estimated models accounted for the spiking statistics of unseen responses with confidence, both over the entire test set and for single trials.

We additionally sought to characterize the estimated model’s predictive performance. The predicted CIFs were computed for stimuli excluded during model estimation and cross-correlated with the observed spiking responses. Examples of the CIFs predicted for one speech and one TORC trial are shown respectively in Fig 3E and 3F, along with the respective correlograms. As in the simulated example, we consistently observed closely matched predicted CIFs and spiking responses that were corroborated with correlogram peaks close to 0-lag. Lag-corrected cosine similarities between observed spiking and predicted CIFs were compute for each neuron to quantify these observations. The median cosine similarity over all PEG neurons for speech responses and TORC responses were 0.4051 and 0.4107, respectively, indicating that estimated models were predictive of spiking responses to unseen stimuli.

To visualize the effect of the receptive field on acoustic stimuli, the 2-D convolution of an example speech spectrogram with the CortSTRF was computed (Fig 3G). The convolved spectrogram illustrates how a family of neurons centered at different frequencies but with the same receptive field shape would represent the example stimulus. The convolved spectrograms, like the CortSTRFs, suggested that PEG neurons were responsive to complex spectrotemporal patterns produced through sparse combinations of primary-cortical features and additionally indicated that the secondary representations of acoustic stimuli have long latencies.

#### CortRF models are more predictive of speech responses than STRF models

The estimation of CortRFs for PEG neurons using point process models was shown to be an effective approach to describing and predicting their spiking responses to acoustic stimuli. We next sought to assess the utility of the primary-cortical representation relative to the spectrogram representation of stimuli by comparing CortRF point process models to STRF point process models of PEG neurons.

STRFs point process models were estimated for each PEG neuron using OMP over an overcomplete dictionary of truncated Gaussian atoms spanning the spectrotemporal feature space (see Methods); here, stimuli were represented using the spectrogram rather than the primary-cortical features. The same training-testing partition of spiking responses used for CortRF estimation was utilized here so that STRFs and CortRFs were estimated and evaluated over the same sets of responses.

**Fig 4:**
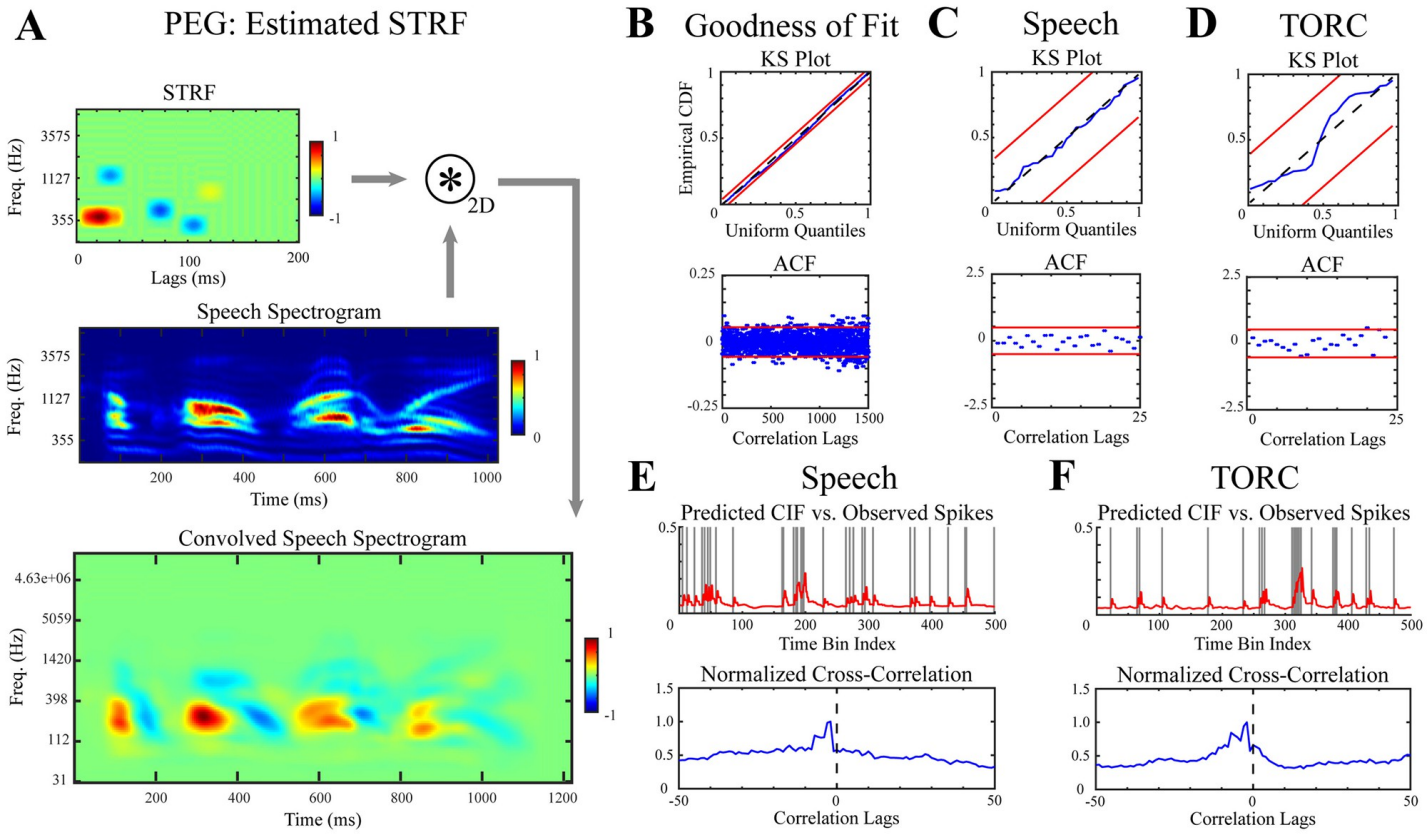
STRF analysis of a PEG neuron. **A.** The estimated STRF of PEG neurons generally provided characterizations of neurons’ selectivity that diverged from the CortRF, as seen here. The 2-dimensional convolution of a speech spectrogram with the estimated STRF suggested shorter latency in the neural representation than when convolved with the CortSTRF. **B–F.** Statistical tests for goodness-of-fit, both for each and over all speech and TORC stimulus repetitions withheld during model estimation, indicated estimated models were well-matched to the observed spiking statistics. Additionally, the withheld spiking responses were closely matched by predicted CIFs. Spectrograms and STRFs are normalized for visualization.

In general, STRFs estimated for PEG neurons did not resemble the estimated CortSTRFs (Fig 4A and S1 Fig); rather, estimated STRFs suggested simpler spectrotemporal feature selectivity as they were comprised of sparse combinations of dictionary atoms. This mismatch was noteworthy since mixtures of an arbitrary number of Gaussian components can approximate most distributions. This difference can also be seen from the convolved speech spectrograms (Fig 4A), where the STRF-convolved speech spectograms tend to either be more positive-valued (i.e. having excitatory effects on spiking responses) or negative-valued (i.e. having inhibitory effects) rather than combinations.

Despite these discrepancies, estimated STRF models were both descriptive and predictive of spiking responses. The KS and ACF tests showed that estimated models accounted for the spiking statistics of unseen responses with confidence, both over the entire test set and for individual trials (Fig 4B–4D). Additionally, the predicted CIFs for test stimuli closely aligned with observed spiking responses as can be seen by inspection and in correlograms (Fig 4E and 4F). The lag-corrected cosine similarities between observed spiking and predict CIFs for TORC and speech responses further corroborated the predictive capability of the STRF point process model: the median similarity for speech responses and TORC responses were 0.4037 and 0.4096, respectively.

**Fig 5:**
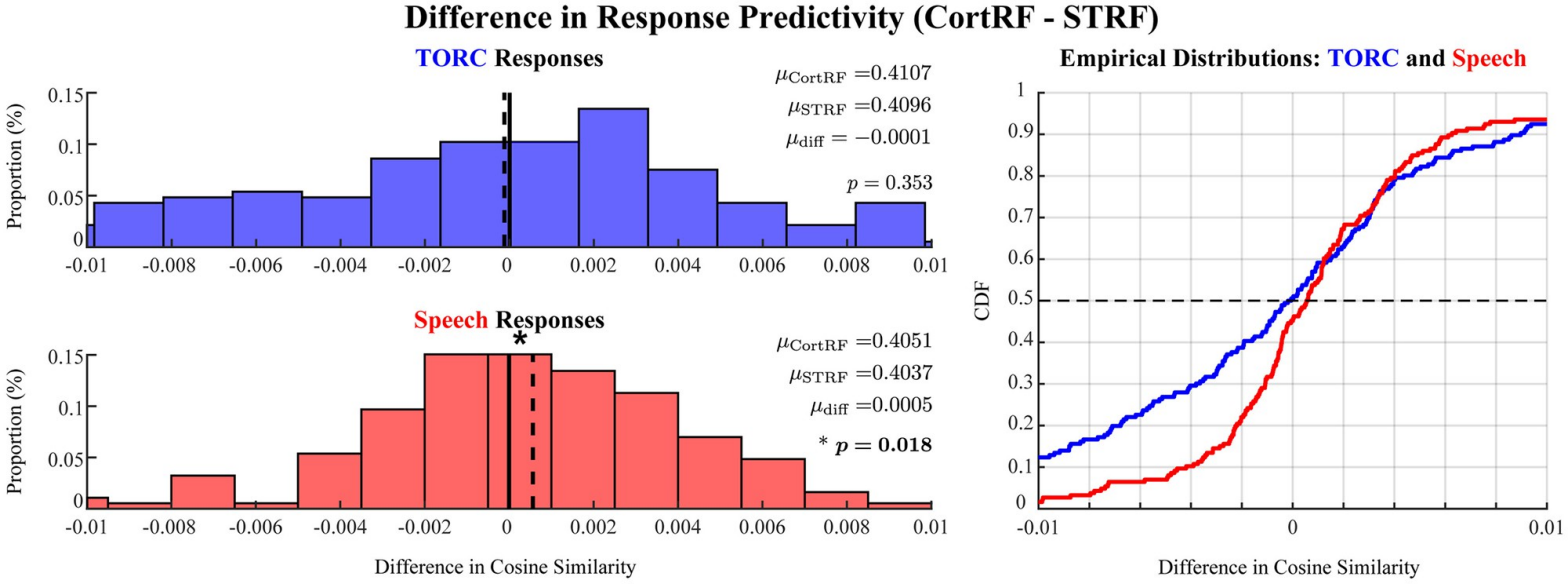
CortRF models of PEG neurons were more predictive of speech responses than STRF models. Quantifying predictive performance by the cosine similarity between the predicted CIFs and observed spiking responses, CortRF models were compared to the STRF models over the same set of speech and TORC responses withheld from model estimation. The histograms of differences in TORC (blue) and Speech (red) response predictions are shown in the left panel. The empirical cumulative density function of these distributions is shown in the right panel. While no difference between the two was found in predicting TORC responses (*p* = 0.353, Wilcoxon signed rank test), a significant advantage in using the CortRF model to predict speech responses was observed (*p* = 0.018, Wilcoxon signed rank test).

The CortRFs and STRFs could both be used to describe the responses of PEG neurons to acoustic stimuli, but it remains unclear if CortRF analysis provided any advantage in modeling PEG activity. As the point process modeling framework produces generative models of neuronal responses, we expect that any advantages to using the CortRF over the STRF would manifest as improved predictions of responses to unseen stimuli. Hence, we computed the differences in cosine similarities involving CortRF-predicted CIFs and STRF-predicted CIFs for both TORC and speech stimuli, individually. The distributions of these differences (CortRF - STRF) are shown in Fig 5. The median difference for TORC response predictions was −0.0001 and was 0.0005 for speech response predictions. Recalling that the set of test responses when evaluating CortRF and STRF models were identical for each neuron, we used the Wilcoxon signed rank test to determine if the median differences in cosine similarity between matched CortRF and STRF pairs were significantly different from 0. The difference in speech response predictions was statistically significant (*p* = 0.018), while the difference in TORC response predictions was not (*p* = 0.353). This suggests that CortRF estimation provided a better characterization of PEG neurons’ feature selectivity because the primary-cortical features of acoustic stimuli, which provide a richer representation than the spectrogram, facilitated the prediction of responses to more complex stimuli.

### CortRFs provide no advantage in predicting A1 responses

The comparative analyses of estimated CortRF and STRF models of PEG neurons demonstrated the utility of the primary-cortical representation of acoustic stimuli. Next, we sought to determine whether the CortRF model was similarly beneficial in modeling the spiking responses of A1 neurons, or it exclusively facilitates the prediction of PEG neuronal responses. We repeated the CortRF and STRF analyses of spiking responses to 30 TORCs and 30 sentences from the TIMIT corpus for 31 A1 neurons from one animal. CortSTRFs and STRFs were estimated using the same data partitioning procedure and algorithm as for PEG neurons. Estimated models for each neuron were evaluated on test responses first for goodness-of-fit using the KS and ACF tests; and then for predictivity by inspecting the correlograms of predicted CIFs vs. observed spiking responses to test stimuli and computing lag-corrected cosine similarities.

**Fig 6:**
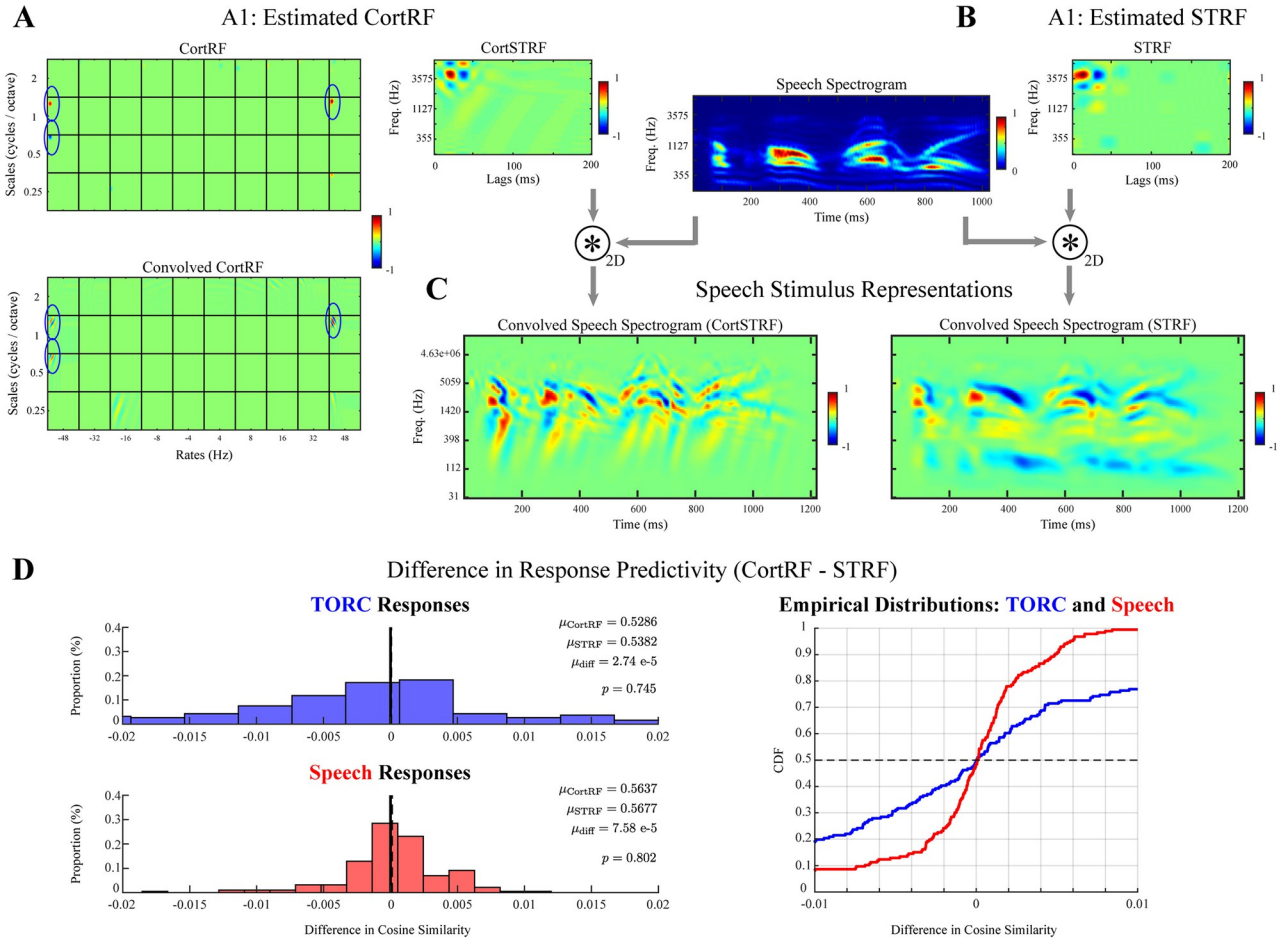
CortRF models of A1 neurons provided no advantage over STRFs in predicting spiking responses to speech or TORCs. **A–B.** CortRF analysis and STRF analysis of A1 neurons characterized their feature selectivity similarly more often than for PEG neurons in the sense that CortSTRFs and STRFs had similar frequency-tuning, latency, and receptive field shape. **C.** The representations of a speech spectrogram, obtained via 2-dimensional convolution with either the CortSTRF (left) or STRF (right) of the same A1 neuron reflected this similarity. The latency and frequencies represented in both convolved spectrograms were much more similar than in PEG neurons. **D.** In further contrast to PEG neurons, there was no significant difference between the predictive performance of CortRF models and STRF models of A1 neurons for either TORC (*p* = 0.750, Wilcoxon signed rank test) or speech stimuli (*p* = 0.802, Wilcoxon signed rank test). Spectrograms and receptive fields are normalized for visualization.

The estimated CortRF and STRF of one A1 neuron are shown in Fig 6A and 6B; the estimated CortRFs, estimated STRFs, and graphical goodness-of-fit tests of all A1 neurons may be found in S2 Fig. In contrast to PEG neurons, CortRF and STRF estimation from A1 neurons described neurons’ feature selectivity more similarly. That is, more A1 neurons had CortSTRFs and STRFs that resembled each other than PEG neurons. The neuron shown in Fig 6 was chosen as a representative example of this trend; the estimated CortSTRF and STRF exhibit similar frequency tunings, latencies, and receptive field shape. The convolution of each with an example speech spectrogram corroborates this observation (Fig 6C). We note that for some A1 neurons (S2 Fig), the estimated CortSTRF and STRF were almost identical, while for others still CortSTRFs and STRFs were highly dissimilar. However, for all A1 neurons, CortRF and STRF estimation produced descriptive models of spiking responses. The KS and ACF tests (S2 Fig), when applied both over the entire test set of responses and individually to each trial, indicated that both CortRF and STRF models of A1 neurons accounted for the spiking statistics of unseen responses with confidence.

Both CortRF and STRF models were also predictive of A1 spiking responses to unseen TORC and speech stimuli. We computed correlograms between predicted CIFs and observed spiking responses and observed dominant peaks at near-zero lags, similar to the correlograms computed for simulated and PEG neurons. Quantitatively, the lag-corrected cosine similarities between predicted CIFs and observed spiking corroborated this assessment. For TORC stimuli, CortRF-predicted CIFs had median cosine similarity of 0.5286 while for STRF-predicted STRFs the median cosine similarity was 0.5382. For speech stimuli, the median cosine similarities of CortRF-predicted CIFs and STRF-predicted CIFs were 0.5637 and 0.5677, respectively. However, for neither TORC nor speech stimuli were there significant differences in cosine similarity (Fig 6D). The median differences for TORC and speech responses were 2.74 × 10^−5^ and 7.58 × 10^−5^, respectively; using the Wilcoxon signed rank test, the differences between CortRF and STRF predictions for the same A1 neurons were determined not to significantly differ from 0 (*p* = 0.750 for TORC stimuli, and *p* = 0.802 for speech stimuli).

The absence of such a difference between CortRFs and STRFs in predicting the responses of A1 neurons suggests that the spectrogram is a sufficiently rich feature space with which to characterize their auditory feature selectivity. This contrasts with the advantage that CortRF estimation provided for PEG neurons, where the use of primary-cortical features facilitated the prediction of speech responses. Our analyses of response predictivity in A1 and PEG neurons thus suggest a transformation of stimulus representations between primary and non-primary auditory cortex, captured in part by the primary-cortical feature space, that facilitates encoding natural acoustic stimuli.

We examined this hypothesized role of the primary-cortical features by comparing the response predictivity of CortRFs and STRFs in both PEG and A1 trained exclusively on either TORC or speech stimuli (S2 Text). These results indicated that, while there are features in speech stimuli that can only be captured when training receptive fields on speech responses (irrespective of are or stimulus feature space), the primary-cortical feature space can represent more speech-like features than the spectrogram. In this sense, the functional role of the primary-cortical representation resembles that of nonlinearities such as synaptic depression and gain normalization that facilitate natural stimulus encoding [51, 52]. Differences in response predictivity by area were not apparent when training exclusively on TORC or speech stimuli. Such a restriction serves as a strong prior on the distribution of stimulus features; the effect of training stimulus likely dominated over differences between PEG and A1. Training receptive fields on both speech responses and TORC responses mitigated this bias, enabling the observation of differences between the two areas in Figs 5 and 6D.

### PEG neurons encode more complex features than A1 neurons

Through comparisons of the predictive performance of CortRF and STRF models for both PEG and A1 neurons, our results thus far suggest that feature selectivity in PEG neurons is better characterized with respect to primary-cortical features than spectrotemporal features directly. Additionally, we have speculated that PEG neurons benefit from a richer feature space because they are selective of more complex spectrotemporal features than A1 neurons. We next addressed this hypothesis formally. In particular, we considered two notions of complexity: concentration of energy and receptive fields shape. If PEG neurons do indeed encode more complex spectrotemporal features, we would expect that their receptive fields should have less concentrated energy and more complex shapes than A1 neurons. Moreover, these differences should occur irrespective of the stimulus representation; hence, we compared the complexities of both the CortSTRFs and STRFs.

**Fig 7:**
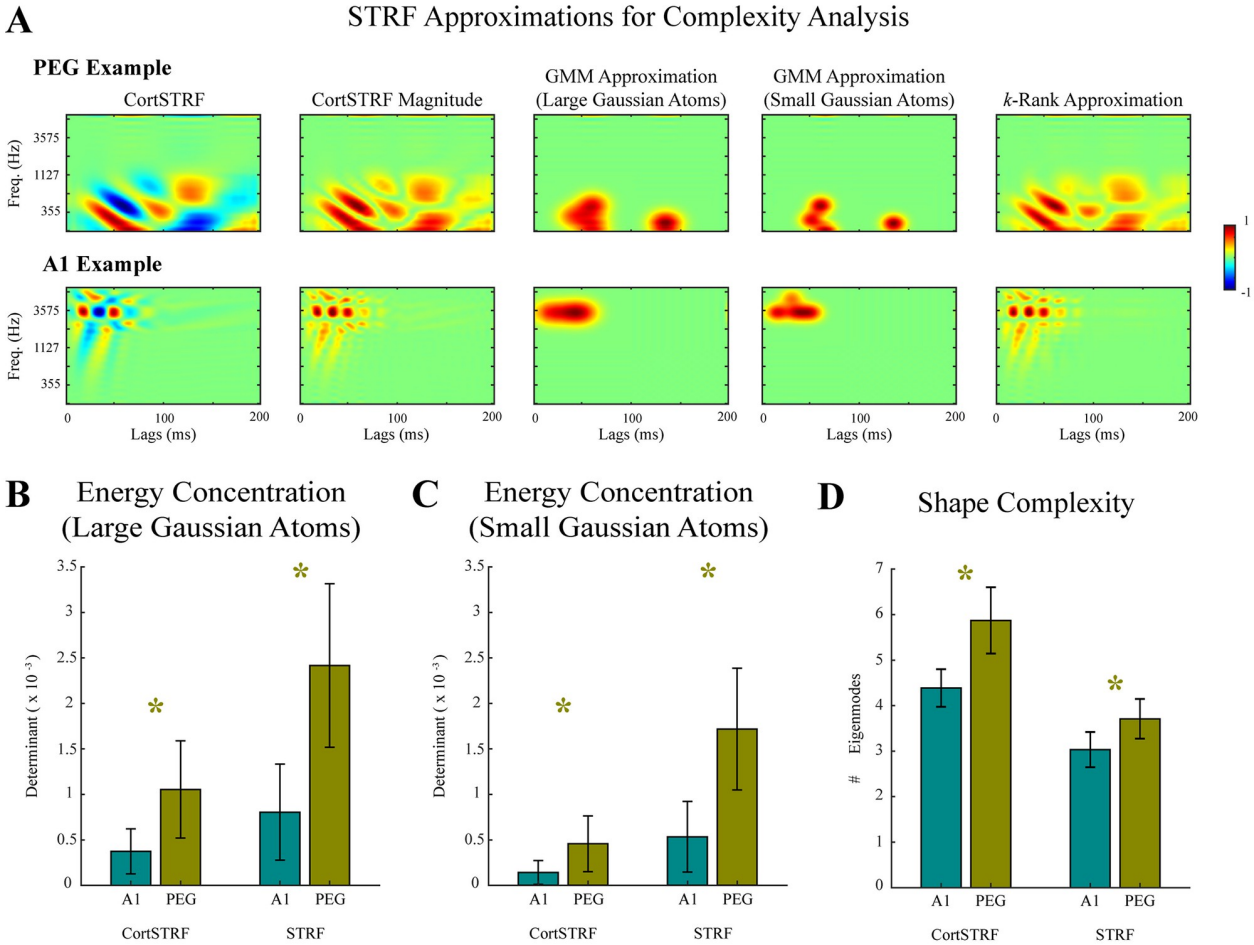
PEG neurons encoded more complex features than A1 neurons. **A.** The complexity of CortSTRFs and STRFs was quantified using two approaches to determine if PEG neurons (top row) were selective of more complex acoustic features than A1 neurons (bottom row). The magnitudes of STRFs were computed (second column) and approximated by a probability distribution function for a Gaussian mixture model (GMM) fit with a boosting algorithm with large- and small-covariance Gaussian weak learners (third and fourth columns, respectively) and by *k* components of its singular value decomposition (fifth column). Here, *k* was the smallest number of singular values that accounted for at least 75% of the spectral power, ensuring the mean-squared errors of all *k*-rank approximations were small. Receptive fields are normalized for visualization. **B.** The concentration of energy in STRFs was measured by the determinant of the covariance of the GMM likelihood; smaller values indicate more concentration of energy. GMMs were fit using a boosting algorithm with large-covariance weak learners. The energy in CortSTRFs and STRFs of PEG neurons was more dispersed than those of A1 neurons (CortSTRF: *p* < 0.001, STRF: *p* = 0.002; Wilcoxon rank sum test). **C.** The analysis of energy concentration in STRFs was repeated with small-covariance weak learners, demonstrating robustness to the choice of base learner and further indicating that the energy in CortSTRFs and STRFs of PEG neurons was more dispersed than those of A1 neurons (CortSTRF: *p* < 0.001, STRF: *p* = 0.002; Wilcoxon rank sum test). **D.** The receptive field shape complexity, quantified by the number of eigenmodes, was higher for both the CortSTRFs (*p* = 0.002, Wilcoxon rank sum test) and STRFs (*p* = 0.023, Wilcoxon rank sum test) of PEG neurons than of A1 neurons.

First, we described the concentration of energy in receptive fields to characterize differences in the ranges of frequencies and latencies to which PEG and A1 neurons were most sensitive. The normalized magnitudes of CortSTRFs and STRFs were first approximated by Gaussian mixture density functions obtained via a boosting algorithm with Gaussian weak learners whose covariances were varied (see Methods). The approximation procedure for the CortSTRF of an example PEG neuron using both large-covariance and small-covariance weak learners is visualized in Fig 7A (top row, third and fourth columns respectively); similarly, an example A1 neuron is also visualized in Fig 7A (bottom row, third and fourth columns). The determinant of an approximate distribution’s covariance was used as a measure of concentration; smaller determinants indicate that the energy in the receptive field is more concentrated. As shown in Fig 7B, using large-covariance weak learners we found that determinants were larger for PEG CortSTRFs than for A1 CortSTRFs (PEG: 1.10 × 10^−3^ ± 1.50 × 10^−3^ vs. A1: 3.74 × 10^−4^ ± 6.87 × 10^−4^; *p* < 0.001, Wilcoxon rank sum test) and for PEG STRFs than for A1 STRFs (PEG: 2.40 × 10^−3^ ± 2.50 × 10^−3^ vs. A1: 8.05 × 10^−4^ ± 1.50 × 10^−3^; *p* = 0.002, Wilcoxon rank sum test). Moreover, we found that determinants were larger for PEG CortSTRFs than for A1 CortSTRFs in approximations using small-covariance weak learners as shown in Fig 7C, (PEG: 6.15 × 10^−4^ ± 4.14 × 10^−4^ vs. A1: 1.90 × 10^−4^ ± 1.77 × 10^−4^; *p* < 0.001, Wilcoxon rank sum test) and for PEG STRFs than for A1 STRFs (PEG: 2.32 × 10^−3^ ± 9.03 × 10^−4^ vs. A1: 7.18 × 10^−4^ ± 5.24 × 10^−4^; *p* = 0.001, Wilcoxon rank sum test). These results suggests that A1 neurons are most sensitive to narrower ranges of frequencies and latencies than PEG neurons. However, measuring the concentration of energy in receptive fields fails to capture the spectrotemporal resolution of features encoded by PEG and A1 neurons. Hence, we next considered the shape complexity of receptive fields.

We computed singular value decompositions of the normalized magnitudes of CortSTRFs and STRFs, and identified the number of eigenvalues that accounted for 75% of the spectral power (i.e. sum of all singular values). This threshold was the smallest proportion for which all *k*-rank approximations of CortSTRFs and STRFs had small mean squared errors (at most 0.001). The *k*-rank approximation of an example PEG CortSTRF is shown in Fig 7A (top row, fifth column), as well as an example A1 CortSTRF in Fig 7A (bottom row, fifth column). A larger number of singular values indicates the linear combination of more eigenmodes are required to approximate the receptive field and hence a more complex shape. As shown in Fig 7C, we found that the necessary number of singular values was significantly larger for PEG CortSTRFs than for A1 CortSTRFs (PEG: 5.87 ± 2.03 vs. A1: 4.39 ± 1.15; *p* = 0.002, Wilcoxon rank sum test); and that the same was true of their respective STRFs (PEG: 3.71 ± 1.22 vs. A1: 3.03 ± 1.08; *p* = 0.023, Wilcoxon rank sum test). These results indicate that not only do PEG neurons tend to selective of wider ranges of frequencies and latencies than A1 neurons, but that features encoded by PEG neurons have more complex shapes than those by A1 neurons.

### CortRF clusters reveal features that segregate by cortical area

By comparing PEG and A1 neurons based on two measures of receptive field complexity, we found that PEG neurons tended to encode more complex features than A1 neurons. Next, we further investigated the differences between features encoded in PEG and A1 through unsupervised clustering of their CortRFs. By clustering the CortRFs estimated from PEG and A1 neurons, we sought to identify groups of receptive fields that corresponded to distinct primary-cortical features and to determine how prevalent these features were in PEG and A1.

**Fig 8:**
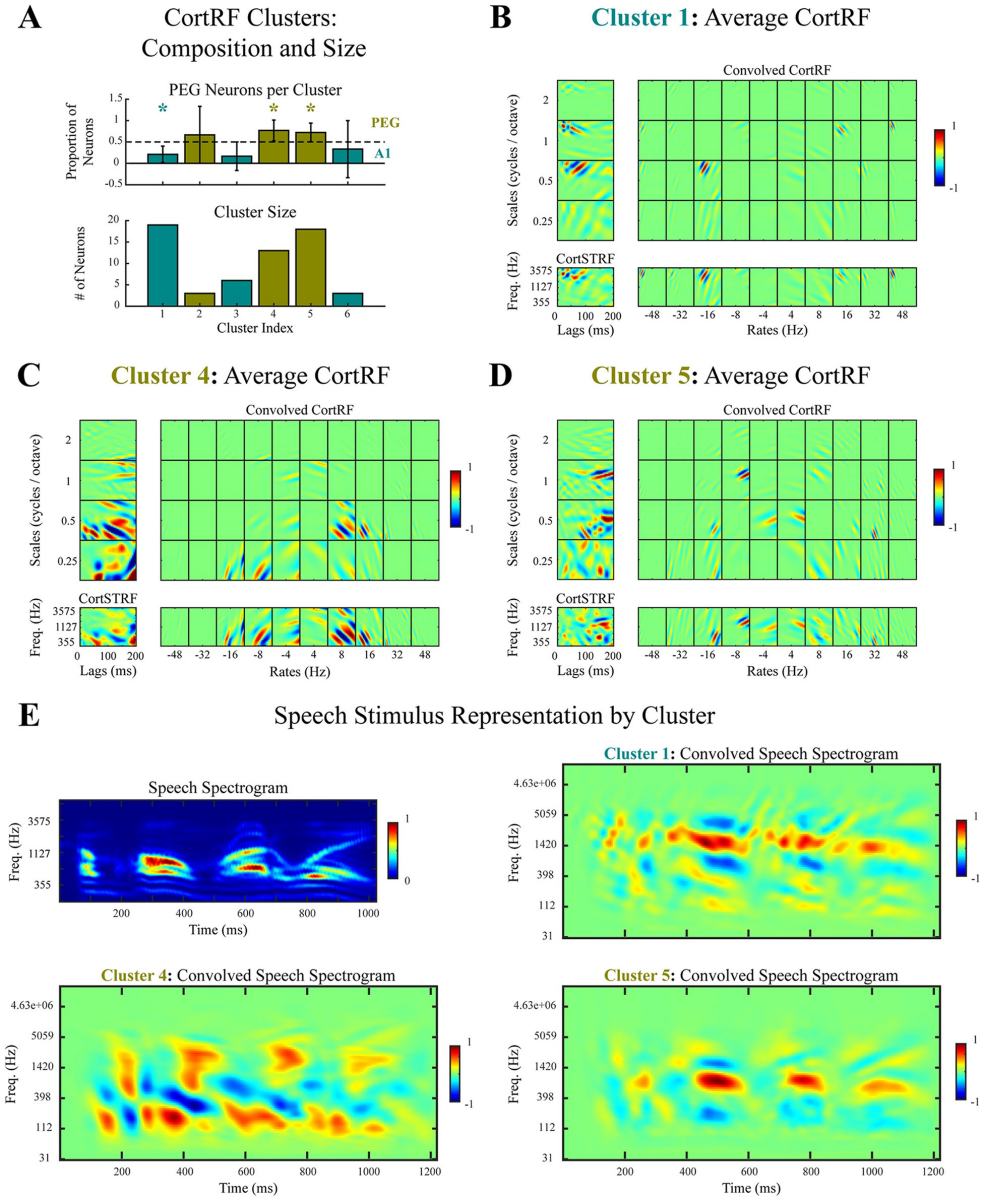
Unsupervised clustering of CortRFs segregated PEG and A1 receptive fields by cortical area. **A.** Spectral clustering, applied jointly to the CortRFs estimated for PEG and A1 neurons, yielded 6 clusters. The proportion of PEG neurons in each cluster ±2 SEM was computed (top), and clusters were designated as representative of distinct PEG features if at least half its members were from PEG (green). A1 clusters were designated similarly (teal). The three clusters with at least ten members (bottom) were inspected further. One was an A1 cluster (Cluster 1), and the other two were PEG clusters (Clusters 4 and 5). Each of these three clusters were confirmed to deviate significantly from chance-level proportion of PEG neurons (*t*-test, *p* < 0.05). Moreover, a Fisher exact test confirms a significant interaction between neurons’ cluster labels (PEG or A1) and cortical area (*p* < 0.05). **B–D.** The average CortRF convolved by the primary-cortical basis functions are displayed for each cluster. The convolved CortRF was marginalized over scales (below), over rates (left), and over both to compute the average CortSTRF. Cluster 1, the A1 cluster, had more energy in fast than slow rate channels (**B**). In contrast, Cluster 4 (**C**) had more energy in slow rate channels; Cluster 5 (**D**) intermediated these two, but did have more energy in slow rate channels. Clusters 4 and 5, the PEG clusters, predominantly had energy in wide-bandwidth scale channels, while Cluster 1 had more narrow bandwidth components. **E.** The representations of a speech spectrogram by each of these clusters were obtained by computing the 2-dimensional convolution with the cluster-average CortSTRFs. The convolved spectrogram of Cluster 1 (top right) had shorter latency and narrower bandwidth than either Cluster 4 or 5. Corroborating observations about the rate-scale composition of Cluster 5, its convolved speech spectrogram had narrower bandwidth than Cluster 4 but longer latency than Cluster 1. Spectrograms and receptive fields are normalized for visualization.

Constructing a similarity matrix by computing the absolute cosine similarities between all pairs of CortRFs, we employed spectral clustering to obtain 6 receptive field clusters in an unsupervised fashion (see Methods). The number of clusters was chosen in relation to the number of small eigenvalues of the graph Laplacian associated with the similarity matrix. For each cluster, we computed the size (i.e. number of neurons in that cluster), the proportion of PEG neurons, and the average convolved CortRFs. Three large clusters consisting of at least 10 neurons were found (clusters 1, 4, and 5, as indexed in Fig 8A); the remaining clusters (S3 Fig) had at most 6 members, and were subsequently excluded in order to avoid biased inferences based on cluster properties. The three large clusters segregated CortRFs by cortical area, as 80% of neurons in cluster 1 were from A1 while 80% and 75% of neurons in clusters 4 and 5, respectively, were from PEG. As indicated in Fig 8A, these proportions deviated significantly from chance level (Cluster 1: *p* = 0.004, Cluster 4: *p* = 0.024, Cluster 5: *p* = 0.028, *t*-test). Moreover, there was a significant interaction between the cluster labels (PEG or A1) and the true cortical areas to which neurons belonged as determined by a Fisher exact test (*p* < 0.001).

The variability of discovered clusters was quantified by comparing intra-cluster similarities to inter-cluster similarities. For all 6 clusters, intra-cluster similarity was significantly larger than inter-cluster similarity (Wilcoxon rank sum test, *p* < 0.05), where the former are an order of magnitude larger than the latter (Table 1).

**Table 1:** Cluster variability.

	Cluster 1	Cluster 2	Cluster 3	Cluster 4	Cluster 5	Cluster 6
**Intra-Cluster**	0.0309 ± 0.0135	0.2255 ± 0.0758	0.0892 ± 0.0588	0.0195 ± 0.0141	0.0073 ± 0.0041	0.0773 ± 0.0981
**Inter-Cluster**	0.0014 ± 0.0005	0.0014 ± 0.0010	0.0025 ± 0.0012	0.0006 ± 0.0003	0.0008 ± 0.0004	0.0005 ± 0.0004

The average intra-cluster and inter-cluster cosine similarities (± 2 SEM) for each of the six discovered clusters were computed and compared. For every cluster, there was a significant difference between intra- and inter-cluster similarity (Wilcoxon rank sum test, *p* < 0.05).

The clusters were visualized (Fig 8B–8D) by computing average convolved CortRFs and their marginalizations over scale and rate channels, both individually and jointly; note that marginalizing over both scales and rates together yields the average CortSTRF of each cluster. When marginalizing across scale channels, we observed that in the A1 cluster, CortRFs tended to have more energy in fast than slow rate channels. In contrast, the Cluster 4 (a PEG cluster) had most of its energy in slow rate channels; Cluster 5 intermediated the A1 cluster and Cluster 4, with energy dispersed across most rate channels. A similar contrast between the A1 and PEG clusters was seen when marginalizing across rate channels. In both PEG clusters, the energy was predominantly restricted to wide bandwidth scale channels, whereas in the A1 cluster, the average CortRF also included narrow bandwidth components. Consequently, the average CortSTRF of the A1 cluster was sensitive to a narrow range of frequencies and time lags, consistent with the analysis of receptive field complexity. These trends were highlighted by visualizing how each cluster would represent an acoustic stimulus. The 2-dimensional convolution of a speech spectrogram with each cluster’s average CortSTRF was computed; the convolved spectrograms are shown in Fig 8E. The convolved spectrogram of Cluster 1 had shorter latency and narrower bandwidth than either Cluster 4 or 5. Corroborating observations about the rate-scale composition of Cluster 5, its convolved speech spectrogram had narrower bandwidth than Cluster 4 but longer latency than Cluster 1.

In summary, unsupervised clustering of estimated PEG and A1 CortRFs corroborated differences in feature selectivity between the two areas quantified through receptive field complexity analyses. Stimulus representations by PEG and A1 clusters exhibited longer latencies and narrower bandwidth, respectively. However, two distinct PEG clusters were found, possibly indicating that there are different classes of secondary stimulus representations that combine primary-cortical features differently.

## Discussion

In this study, we demonstrated that sparsely estimated receptive fields of neurons in ferret PEG with respect to primary-cortical features of acoustic signals provides a hierarchical model that decomposes the complex spectrotemporal selectivity of secondary auditory neurons in a biologically interpretable manner. We found that estimated CortRFs provided a significant advantage over estimated STRFs in predicting the responses of PEG neurons to speech, an advantage that was absent when comparing CortRF- and STRF-based predictions of A1 spiking responses. Quantitative comparisons between the complexity of PEG and A1 CortSTRFs indicated that the improved predictions of speech responses in PEG were due to more complex spectrotemporal selectivity, a trend corroborated when comparing STRFs from the two areas. Moreover, unsupervised clustering of PEG and A1 CortRFs revealed that the sparse combinations of primary-cortical features into which complex spectrotemporal features were decomposed had distinct patterns that segregated by cortical area. Our findings thus suggest that the proposed hierarchical model of spectrotemporal selectivity in PEG captures a transformation in neural representations of acoustic signals between A1 and PEG that serves to encode complex features of natural stimuli.

### CortRF analysis and related approaches

CortRF analysis utilizes two central assumptions: receptive field sparsity and choice of feature space. Sparse priors of various forms for receptive field estimation have previously been explored. In the following, we speculate about their trade-offs. For point process GLMs and linear-Gaussian models of auditory neurons, biasing receptive field estimates towards zero using regularized maximum likelihood estimation or boosting has been demonstrated to improve predictions of responses to novel acoustic stimuli [17, 24]. The greedy estimation algorithm used in this work, OMP, is closely related to these methods [48, 49], and its usage to encourage sparsity was motivated in part by model generalizability. Empirical Bayes estimators have also been demonstrated to capture the spatiotemporal localization of receptive fields in early visual areas [18, 19]. However, similar spectrotemporal localization assumptions could not be made for PEG neurons; in fact, PEG receptive fields were differentiated from those in A1 by their comparative lack of localization. Both regularized and Bayesian estimators scale poorly with feature dimensionality. However, since OMP iteratively selects a subset of features over which the receptive field is estimated, it is more tractable to implement when features are high-dimensional.

The tractability of receptive field estimation has previously been addressed through assumptions about the stimulus feature space with respect to which models are estimated. Parameterizing receptive fields with basis functions is one such assumption that yields a potentially low-dimensional representation [8]. Gabor functions, closely related to the primary-cortical basis functions used in this work [46], have been established as useful idealized receptive field models of both auditory and visual neurons [8, 53, 54], and applied in sparse coding models [55–57]. CortRF analysis essentially estimates a sparse representation of an STRF using an overcomplete dictionary of Gabor-like spectrotemporal functions where the weights of dictionary atoms comprise the CortRF. Recently, the use of spline functions has been proposed to parsimoniously capture the localization and smoothness of visual receptive fields [20], though, as noted previously, similar assumptions for PEG neurons are somewhat tenuous. Low-rank factorized receptive field models also present a scalable alternative and have been utilized to obtain low-dimensional characterizations of auditory and visual receptive fields [9, 25]. Crucially, factorized models and basis functions still parameterize receptive fields with respect to early-stage stimulus features (i.e. images or spectrograms) and hence, when applied to non-primary sensory neurons, would characterize complex feature selectivity without accounting for intermediate neural representations. In contrast, utilizing primary-cortical features of acoustic stimuli enabled us to describe a hierarchical transformation in stimulus representations between A1 and PEG.

In this sense, CortRF analysis imposes a similar modeling assumption as convolutional neural network (CNN) models of higher-order auditory and visual areas, though in a more restricted manner. Namely, the architecture of CNNs require the stimulus representation at a given network layer to be based on the composition of hierarchical convolutions over the preceding layers. In our proposed model, a PEG neuron can analogously be thought of as a single unit in the second layer of a CNN, while a CortRF are layer weights connecting the preceding layer (A1, in this analogy). Post hoc comparisons with neural data from auditory and visual cortex have indicated that task-optimized CNNs produce models whose intermediate and deeper layers correspond to neural responses (recorded via fMRI and electrophysiologically in separate instances) in low- to mid-level and downstream areas, respectively [32, 37, 40, 44, 45]. However, the correspondence is ambiguous; for example, several shallower CNN layers may be seen to correlate with primary sensory cortical responses. Hence, while existing CNN models indeed describe a hierarchical scheme by which stimuli are encoded for higher-order sensory processing, it is difficult to ascertain a detailed correspondence with sensory cortical organization. CortRF analysis in the present study, while comparatively limited in its scope to single neurons in a secondary auditory area, does suggest a hypothesis about secondary representations of acoustic stimuli. Our results suggest that secondary stimulus representations in PEG arise through sparse combinations of primary-cortical features. Though PEG neurons individually pooled few primary-cortical features, they collectively spanned a large portion of the primary-cortical feature space. Moreover, improved speech response predictions in PEG neurons by CortRF models suggest that hierarchical representations in auditory cortex facilitate encoding natural stimuli.

Recently, a theoretical study proposed a similar hypothesis about non-primary auditory cortical stimulus representations [58]. The study sought to explain how low-level spectrotemporal features may be combined downstream using spectrotemporal kernels learned to approximate natural stimuli as an analog of the primary-cortical model. Secondary features were modeled as sparse combinations of these learned spectrotemporal kernels, which consisted of a diverse range of rates and scales. Secondary features were combinations of positively or negatively weighted kernels, reminiscent of the sparse CortRFs estimated in the present study. While we did not explore if primary-cortical feature combinations characterized by CortRFs matched features of natural stimuli, it is notable that a sparse hierarchical encoding scheme can emerge from statistical learning over the wide range of sounds encountered in natural environments.

### Feature selectivity in primary versus secondary auditory cortex

Although studies comparing the tuning properties of primary and secondary auditory cortical neurons recorded passively in ferrets (or other animals) are limited, our results comparing the receptive field properties of PEG and A1 neurons were consistent with previous findings. The complexity and clustering analyses in the present study showed that A1 receptive fields had more compact spectrotemporal tuning (i.e. narrower bandwidth and integration window) than PEG neurons while also having less complex receptive field shapes and were hence selective of simpler features. These results corroborate previous studies that showed ferret PEG neurons had broader tuning properties than A1 [59, 60]. Additionally, the STRFs of PEG neurons have been shown to be less sparse than STRFs of A1 neurons [60], mirroring the greater dispersion of energy in PEG receptive fields described in this study.

Clustering CortRFs highlighted differences in feature selectivity between PEG and A1, but also within PEG. Two CortRF clusters consisting mostly of PEG neurons were found, differentiated by the presence or absence of high-rate and high-scale components observed in the cluster of mostly A1 neurons. This separation within PEG might be reflective of different schemes for combining primary-cortical features to form secondary representations. Interestingly, the aforementioned theoretical study of hierarchical auditory processing noted different classes of secondary features as well [58]. Secondary units either pooled spectrotemporal kernels that were frequently coactive or pooled positively and negatively weighted kernels that were unlikely to occur concurrently. Additionally, a recent neural network approach to STRF characterization in ECoG data suggests the emergence of different classes of spectrotemporal features [42].

Comparable receptive field studies in animals are sparse, but STRF analyses in ECoG studies of human speech perception do suggest different classes of feature transformations in superior temporal gyrus (STG), a non-primary area in human auditory cortex. Speech responses in STG have been shown to be selective of various speech-specific features that endure over differing timescales [5, 61, 62]. In particular, spatially organized differences in receptive fields estimated using speech stimuli have been observed within STG, quantified by modulation transfer functions that summarize changes in scale and rate components of STRFs [61]. Namely, receptive fields varied from high-rate low-scale selectivity to low-rate high-scale selectivity, tracking either fast temporal dynamics of speech across frequencies or slow temporal dynamics of speech with high spectral variation. Despite the substantial divergence between the higher-order acoustic representations of ferrets and humans [63], these results are consistent in suggesting that secondary auditory cortical areas have classes of hierarchical features that combine low-level representations distinctly in order to support higher-order processing, though the nature of such classification is likely species-specific. The crucial contrast with previous studies is that our results indicate that the hierarchical auditory cortical organization is based on single-unit responses. Thus, the proposed CortRF model and related analyses provide a framework that, when applied at scale, could describe the emergence of non-primary features that support higher-order auditory processing.

## Methods

### Experiments and stimuli

Receptive field analysis of ferret PEG and A1 neurons was performed on data recorded at the Neural System Laboratory at the Institute for Systems Research at University of Maryland, College Park. Data from PEG neurons were recorded from three animals, and include recordings previously published in [64]. Data from A1 neurons were recorded from one animal and include recordings previously published in [65]. We refer the reader to each work for a complete description of experimental procedures.

Ferrets were presented with two types of acoustic stimuli: speech samples arbitrarily selected from the TIMIT corpus; and temporally orthogonal ripple combinations (TORCs). TORCs are broadband spectrotemporally modulated noise signals [16]. Sets of 30 distinct speech and TORC stimuli were presented, each repeated between 4 − 6 times in randomized order. Stimulus presentation lasted for 1.5 or 3 seconds; the duration varied across recording sessions but not within a set of speech or TORC samples. The first 0.5 seconds after stimulus onset are omitted from all spiking responses in order to exclude transient effects in all analyses. Spiking responses, initially sampled at 1 kHz, were binned using 5 ms long bins.

We utilized TORCs consisting of a dynamic spectrotemporal profile characterized by the combinations of either 6 temporally orthogonal ripples (4 − 24Hz TORCs) or 12 temporally orthogonal ripples (4 − 48Hz TORCs). Ripples composing the TORCs had linear sinusoidal spectral profiles; peaks were equally spaced from between 0.25 − 1.2 cycles per octave. The ripple envelopes drift temporally either up or down a logarithmic frequency axis spanning 0.125 − 4 kHz, 0.25 − 8 kHz, or 0.5 − 16 kHz at a constant velocity; TORC frequencies ranges were chosen based on the best frequencies of the recorded population. All speech and TORC acoustic signals were initially sampled at 40 kHz; if the TORCs used had a maximum frequency of at most 8 kHz, both stimuli were downsampled to 16 kHz.

### Primary-cortical representation of auditory stimuli

Responses in primary auditory cortex are modeled by the cortical representation of auditory stimuli [46]. The cortical analysis consists of two stages: the extraction of the auditory spectrogram, modeling the highly non-linear transformation of the inner ear [66]; and its multi-scale multi-rate decomposition via a wavelet-like analysis operating along both the spectral and temporal axes [46]. We refer the reader to the respective references for a more detailed description, but provide an overview for completeness.

First, an affine wavelet transform modeling the basilar membrane is applied to the acoustic signal. Nonlinear rectification and compression, which model the effects of the hair-cell stage, are employed next, after which a lateral inhibitory network is used to sharpen features. The rectified output is then integrated over short time windows to produce the auditory spectrogram. The cortical transformation of the auditory spectrogram is, in essence, a 2-dimensional wavelet transformation whose spectrotemporal basis functions perform multi-resolution analysis along both the spectral and temporal axes separably. Scale (i.e. bandwidth) channels represent the spectrogram with different degrees of spectral smoothing; each of *S* complex-valued spectral filters are parameterized by their spectral tuning (cycles/octave). Multi-rate analysis is performed by applying *R* antiphasic pairs of modulation-selective filters parameterized by their center rates (Hz). Example basis functions of the cortical analysis is shown in Fig 1.

We generate spectrograms with *F* = 32 frequency channels that log-uniformly span the frequency range of the TORC stimuli presented to the neuron, and whose highest frequency corresponds to the Nyquist frequency. Namely, for TORCs whose frequencies range between 0.125 − 4 or 0.25 − 8 kHz (and were downsampled to 16 kHz), the spectrogram frequency channels span 0 − 8 kHz; and for TORCs with frequencies spanning 0.5 − 16 kHz (whose sampling rates remained at 40 kHz), the spectrogram frequency channels span 0 − 20 kHz. Spectrograms were generated with 5ms long frames, matching the bin sizes of spiking observations. The cortical basis filters consist of *S* = 4 scale channels and *R* = 5 rate channels. The spectral tuning parameters of the scale filters, in octave intervals, spanned 0.25 − 2 cycles per octave; the rate filters were centered at ±{4, 8, 16, 32, 48} Hz, covering the range of rates used to generate TORC stimuli. The cortical representation of stimuli thus consisted of *F* ⋅ *S* ⋅ 2*R* = 1280 frequency-scale-rate channels, and like the spectogram, had 5ms long time frames.

We note the following regarding the use of two frequency axes for different groups of TORC stimuli. Though different frequency ranges were used, the spectrogram channels’ critical frequencies were spaced using the same intervals, log-uniformly; hence, they differ simply by translation along the log-frequency axis. Thus, group-level comparative analyses of receptive fields describe properties indifferent to absolute frequency.

### Point process model-based cortical receptive field estimation

In order to estimate the cortical receptive fields (CortRF) of PEG and A1 neurons, we treated their spiking responses as observations of discretized point processes used to fit generalized linear models (GLM) to the conditional intensity function (CIF) [21, 23]. That is, the probability of a spike event in a time bin is equated to the CIF weighted by the bin size, where the CIF is non-linear function of the linear combination of spiking history and stimulus history regressors. This generative approach is used for neural encoding models and has been shown to be more predictive of neural spiking responses than linear models [22–24]. The parameters of a GLM can be estimated by maximizing the likelihood of the observed spiking data, and the goodness-of-fit of the estimated model can be evaluated using results from point process theory. In the following, we first formulate CortRF estimation as a maximum likelihood problem, then describe our estimation procedures and measures for model goodness-of-fit.

#### Problem formulation

Let $\{n_{t,j}^{\left(k\right)}{\}}_{t=1}^{T}$, a sequence of *T* Bernoulli observations, denote the spiking response binned with bin size Δ = 5ms to the *j*^th^ repetition of the *k*^th^ stimulus, where trial indices range from *j* = 1, …, *J*_*k*_ and the stimulus indices from *k* = 1, …, *K*. The success probabilities of the spiking process, conditioned on the stimulus and recent spiking history, are given by the conditional intensity function (CIF) $\{\lambda_{t,j}^{\left(k\right)}\Delta {\}}_{t=1}^{T}$. The CIF is modeled as a GLM with logistic link function:

$$\begin{array}{c} \lambda_{t,j}^{\left(k\right)}\Delta =\frac{e^{\mu {+h_{t,j}^{\left(k\right)}}^{⊤}\omega {+s_{t}^{\left(k\right)}}^{⊤}\theta }}{1+e^{\mu {+h_{t,j}^{\left(k\right)}}^{⊤}\omega {+s_{t}^{\left(k\right)}}^{⊤}\theta }}. \end{array}$$ (1)

The model is parameterized by the baseline firing rate parameter, *μ*, the history modulation vector, ***ω***, and the stimulus modulation vector (i.e. the receptive field), ***θ***. The recent spiking history, $h_{t,j}^{\left(k\right)}$, and the stimulus, $s_{t}^{\left(k\right)}$ collectively constitute the model regressors.

The modulation of the CIF due to recent spiking history is assumed to depend on a short integration window. The integration window consists of *M* = 5 subdivisions of non-overlapping windows with lengths $\{W_{m}{\}}_{m=1}^{M}=\{2^{m-1}{\}}_{m=1}^{M}$. Accordingly, the spiking history vector has elements

$$\begin{array}{c} h_{t,j}^{\left(k\right)}={\left[h_{t,j,1}^{\left(k\right)},\ldots ,h_{t,j,m}^{\left(k\right)},\ldots h_{t,j,M}^{\left(k\right)}\right]}^{⊤}. \end{array}$$

These are defined as

$$\begin{array}{c} h_{t,j,m}^{\left(k\right)}≔\sum_{i=t-1-b_{m}}^{t-1-b_{m-1}}n_{i,j}^{\left(k\right)}, \end{array}$$ (2)

where $b_{m}=\sum_{l=1}^{m}\;W_{l}$ with *b*_0_ = 0. Thus, the history integration window covers *L* = 31 bins (equivalently, 155ms) with *M* = 5 parameters.

We assume the stimulus history dependence of spiking responses is limited to the preceding 200ms, i.e. *P* = 40 bins. The stimulus vector $s_{t}^{\left(k\right)}$ is the flattened cortical representation with *F* ⋅ *S* ⋅ 2*R* ⋅ *P* = 51200 elements. However, for tractability, we assume the receptive field is sparsely comprised of elements of an overcomplete dictionary of truncated Gaussian atoms. That is, for each rate-scale channel, truncated Gaussian kernels spanning 5 frequency channels and 5 bins cover the *F* × *P* spectro-temporal receptive field with stride 3. Two such atoms are visualized in the cortical receptive field shown in Fig 1, circled in blue. Thus, the receptive field is equivalently represented by ***θ*** = **Ψ*ξ***, where ***ξ*** is a sparse lower-dimensional vector and the **Ψ** the dictionary of Gaussian atoms. The stimulus modulation term has the following equivalency: $\theta^{⊤}s_{t}^{\left(k\right)}=(\Psi \xi {)}^{⊤}s_{t}^{\left(k\right)}=\xi^{⊤}{\tilde{s}}_{t}^{\left(k\right)}$, where ${\tilde{s}}_{t}^{\left(k\right)}=\Psi^{⊤}s_{t}^{\left(k\right)}$. The receptive field is thus tractably estimated by optimizing over ***ξ***.

The Bernoulli log-likelihood of the spiking statistics of the *j*^th^ repetition of the *s*^th^ stimulus is given by

$$\begin{array}{c} \begin{array}{cc} \ell_{j}^{\left(k\right)}\left(w\right) & =\sum_{t=1}^{T}[n_{t,j}^{\left(k\right)}\text{log}(\lambda_{t,j}^{\left(k\right)}\Delta )+(1-n_{t,j}^{\left(k\right)})\text{log}(1-\lambda_{t,j}^{\left(k\right)}\Delta )]\\ & =\sum_{t=1}^{T}[n_{t,j}^{\left(k\right)}{x_{t,j}^{\left(k\right)}}^{⊤}w-\text{log}(1+e^{{x_{t,j}^{\left(k\right)}}^{⊤}w})]\\ & ={n_{j}^{\left(k\right)}}^{⊤}X_{j}^{\left(k\right)}w-1^{⊤}\text{log}(1+e^{X_{j}^{\left(k\right)}w}), \end{array} \end{array}$$ (3)

where the log(⋅) and *e*^(⋅)^ operations in the third line are element-wise. In the compact notation utilized in Eq (3), the parameter vector ***w*** = [*μ*, ***ω***^⊤^, ***ξ***^⊤^]^⊤^, and the augmented model regressors $x_{t,j}^{\left(k\right)}={\left[1{,h_{t,j}^{\left(k\right)}}^{⊤}{,{\tilde{s}}_{t}^{\left(k\right)}}^{⊤}\right]}^{⊤}$. Additionally, the covariate matrix is defined as $X_{j}^{\left(k\right)}≔{\left[x_{1,j}^{\left(k\right)},\ldots x_{T,j}^{\left(k\right)}\right]}^{⊤}$ and the spiking observations are denoted as the vector $n_{j}^{\left(k\right)}≔{\left[n_{1,j}^{\left(k\right)},\ldots ,n_{T,j}^{\left(k\right)}\right]}^{⊤}$; the CIF in vector form is expressed similarly as $\lambda_{j}^{\left(k\right)}≔{\left[\lambda_{1,j}^{\left(k\right)}\Delta ,\ldots ,\lambda_{T,j}^{\left(k\right)}\Delta \right]}^{⊤}$.

The estimated parameters $\hat{w}$ are obtained by maximizing the log-likelihood of all spiking observations across the repeated presentations of *K* stimuli, i.e. by maximizing $\ell (w)=\sum_{k=1}^{K}\sum_{j=1}^{J_{k}}\ell_{j}^{\left(k\right)}(w)$. With the total covariate matrix $X≔{\left[{X_{1}^{\left(1\right)}}^{⊤},\ldots ,{X_{J_{1}}^{\left(1\right)}}^{⊤},\ldots ,{X_{j}^{\left(k\right)}}^{⊤},\ldots ,{X_{J_{K}}^{\left(K\right)}}^{⊤}\right]}^{⊤}$, and similarly defined total spiking observation vector $n≔{\left[{n_{1}^{\left(1\right)}}^{⊤},\ldots ,{n_{j}^{\left(k\right)}}^{⊤},\ldots ,{n_{J_{K}}^{\left(K\right)}}^{⊤}\right]}^{⊤}$, the total data log-likelihood may be expressed as

$$\begin{array}{c} \ell \left(w\right)≔n^{⊤}Xw-1^{⊤}\text{log}(1+e^{Xw}), \end{array}$$ (4)

and estimated parameters obtained by solving the maximum likelihood problem

$$\begin{array}{c} \hat{w}≔\underset{w}{\text{arg}\;\text{max}}\;\ell \left(w\right). \end{array}$$ (5)

#### Sparse cortical receptive field estimation

Though utilizing an overcomplete dictionary to represent the primary-cortical feature space reduces the number of parameters, the problem in Eq (5) remains high-dimensional. As such, direct solutions to Eq (5) would be prone to overfitting limited data. To mitigate overfitting, a sparsity constraint on $\hat{w}$ is imposed by using the Orthogonal Matching Pursuit (OMP) to estimate the model [48, 49], as detailed in Algorithm 1. The use of sparse priors to estimate receptive fields from responses to natural stimuli has been demonstrated to improve the their ability to predict responses to unseen stimuli [17, 24].

**Algorithm 1** Point Process Orthogonal Matching Pursuit (**OMP**)

**Input:** ***n***, ***X***, s*

**Output:** $\hat{w}$

1: *S*^(0)^ = ∅

2: ${\hat{w}}_{\left(0\right)}=0$

3: **for** *r* = 1 to s* **do**

4:  $\lambda ({\hat{w}}_{\left(r-1\right)})=e^{{\hat{w}}_{\left(r-1\right)}^{⊤}X}/\left(1+e^{{\hat{w}}_{\left(r-1\right)}^{⊤}X}\right)$

5:  $\nabla ℒ({\hat{w}}_{\left(r-1\right)})=X\left(n-\lambda ({\hat{w}}_{\left(r-1\right)})\right)$

6:  $j=\text{arg}\;{\text{max}}_{i\notin S^{\left(r-1\right)}}{\left|\nabla L({\hat{w}}_{\left(r-1\right)})\right|}_{i}$

7:  *S*^(*r*)^ = *S*^(*r*−1)^ ∪ {*j*}

8:  ${\hat{w}}_{\left(r\right)}=\text{arg}\;{\text{max}}_{\text{supp}(w)\subseteq S^{\left(r\right)}}L(w)$

9: **end for**

10: **return** $\hat{w}={\hat{w}}_{\left(s^{*}\right)}$

OMP iteratively selects the model support set: at each iteration, the out-of-support parameter that maximizes the magnitude of the partial gradient with respect to it is added to the model support; the log-likelihood is then maximized over the updated support set. The sparsity level s*, i.e. the maximum size of the support set, is a hyperparameter that restricts the number of non-zero elements in the sparse estimate $\hat{w}$.

CortRFs were estimated by applying the following procedure to each neuron. First, two-fold cross-validation was used to determine the optimal sparsity level s* over the range of values *s* = 1, …, 100. Spiking response data were partitioned into a training set consisting of the responses to 24 TORCs and 24 sentences, and a testing set consisting of the remaining 6 TORC and 6 speech responses. The training set was used for model estimation as well as cross-validation, while the testing set was used exclusively to evaluate the model’s goodness-of-fit and its predictivity of responses to data unseen during training. The range of rate-scale channels covered in the TORC and speech testing sets were verified to be redundant with the range of rate-scale channels covered in the training sets. To cross-validate for s*, the training set was divided evenly in two, with each subset consisting of 12 TORC and speech responses; a model was fit to the first subset and the log-likelihood computed with respect to the second, and vice versa. The sparsity level s* was chosen to maximize the sum of the log-likelihoods over each training subset. Then, the s*-sparse greedy estimate of the GLM parameters were then obtained by solving the maximum likelihood problem in Eq (5) over the training set using OMP.

#### Evaluating model goodness-of-fit

We validate the OMP-estimated point process GLMs for neuronal spiking responses by inspecting both the statistical fit and predictive capability of estimated models on the test set of spiking responses. The statistical goodness-of-fit of point process models can be evaluated using the time-rescaling theorem [50], which establishes that the time-rescaled interspike intervals should be independent and distributed uniformly on the interval (0, 1). Two graphical tests are employed to validate these properties [21, 49, 50]: the autocorrelation function (ACF) test is used to determine if the interspike intervals are uncorrelated; and the Kolmogorov-Smirnov test is used to determine if the time-rescaled interspike intervals are uniformly distributed. The ACF and KS tests were applied both individually and collectively to each repetition of every stimulus in the test set. Following [50], 95% confidence intervals for the KS test are computed as $\pm 1.96/\sqrt{N}$ around the 45° line; similarly, the 95% confidence interval for the ACF test is computed as $\pm 1.96/\sqrt{N}$. Here, *N* denotes the total number of spikes.

The predictive capability of an estimated model was evaluated by comparing the estimated CIF with the observed spiking response for each repetition of every stimulus in the test set. The cross-correlation between the estimated CIF and the observed spiking response visually indicates their alignment. The peak cross-correlation occurred at non-zero lags (differing between but constant for each neuron), reflecting the group delay of the history-dependence filter. The alignment between the estimated CIF and observed spiking response was quantified using the cosine similarity between the average estimated (and group delay-corrected) CIF and the PSTH of the observed spiking response.

### Spectrotemporal receptive field estimation

We compared the characterization of neurons’ stimulus tuning by CortRF estimation to spectrotemporal receptive field (STRF) estimation in order to examine the utility of the primary-cortical feature space over the spectrogram. We estimated STRFs using a point process GLM using training and testing data partitions identical to the partitions used for cortical receptive field estimation. The procedure was identical to that for estimating the cortical receptive fields, excepting the representation of the stimulus regressors. Stimulus history dependence was assumed to be limited to the preceding 200ms, i.e. 40 bins, so that the size of the STRF is consistent with each rate-scale channel of the CortRF. For consistency in maximum likelihood estimation, the STRF was assumed to be sparsely comprised of elements of an overcomplete dictionary of truncated Gaussian kernels; the weights of these atoms were estimated using OMP. A closely related approach to sparse STRF estimation was utilized in [24], but used *ℓ*_1_-regularization of the STRF rather than greedy estimation over a dictionary of features. Goodness-of-fit and predictivity were evaluated in the same manner as for the cortical receptive field estimation problem.

### Complexity analysis of STRFs

The complexity of auditory features for which a neuron was selective was characterized by the concentration of energy in and shape of its STRF. Measuring the concentration of energy in the STRF describes the range of frequencies and latencies to which neurons were most sensitive. This is complemented by a measure of complexity of features in that range. The distinction between these descriptors of complexity is highlighted when comparing a Gabor function to a Gaussian density, where the latter corresponds to the envelope of the former. The energy is concentrated similarly in both functions, but the Gabor function has a more complex shape.

To quantify the concentration of an STRF’s energy, we approximated the normalized magnitude of the STRF by the density function of a Gaussian mixture model and computed the determinant of the distribution’ covariance matrix. Smaller values of the determinant indicate that energy in the STRF is more concentrated. The Gaussian mixture density approximation was obtained iteratively by a boosting algorithm (Algorithm 2) in which the weak learners were Gaussian kernels with fixed and equal covariances whose means are determine at each iteration. Defining *y* to be the *F* × *P* normalized magnitude of the STRF, ${\hat{y}}^{\left(m\right)}$ to be its approximation after *m* iterations, and $r^{\left(m\right)}≔y-{\hat{y}}^{\left(m-1\right)}$, the algorithm was as follows.

**Algorithm 2** Gaussian Mixture Density Function Fitting

**Input:** $y\in {\mathbb{R}}_{+}^{F\times P}$, *M*, Σ

**Output:** $\hat{y},m^{*}$

1: ${\hat{y}}^{\left(0\right)}=0$

2: **for** *m* = 1 to *M* **do**

3:  $r^{\left(m\right)}≔y-{\hat{y}}^{\left(m-1\right)}$

4:  $\mu^{\left(m\right)}=[f_{\text{max}},p_{\text{max}}]≔\text{arg}\;{\text{max}}_{f,p}{\left\{{\left[r^{\left(m\right)}\right]}_{f,p}\right\}}_{f=1:F,p=1:P}$

5:  ${\left\{{\left[z^{\left(m\right)}\right]}_{f,p}\right\}}_{f=1:F,p=1:P}={\left\{\phi_{\left(\mu^{\left(m\right)},\Sigma \right)}(f,p)\right\}}_{f=1:F,p=1:P}$

6:  $\hat{\alpha }={\left[{\hat{\alpha }}^{\left(1\right)},\ldots ,{\hat{\alpha }}^{\left(m\right)}\right]}^{⊤}=\text{arg}\;{\text{min}}_{\alpha :\alpha >0,1^{⊤}\alpha =1}\|y-\sum_{l=1}^{m}\alpha^{\left(l\right)}z^{\left(l\right)}{\|}_{F}^{2}$

7:  ${\hat{y}}^{\left(m\right)}=\sum_{l=1}^{m}{\hat{\alpha }}^{\left(l\right)}z^{\left(l\right)}$

8:  $\varepsilon^{\left(m\right)}=\|y-{\hat{y}}^{\left(m\right)}{\|}_{F}^{2}$

9: **end for**

10: m* = min_*m*=1:*M*_*ε*^(*m*)^

11: **return** $\hat{y}={\hat{y}}^{\left(m^{*}\right)}$, m*

Here, *ϕ*_(***μ***, Σ)_(*f*, *p*) denotes the Gaussian probability density function with mean ***μ*** and covariance Σ evaluated at the frequency channel and time lag index (*f*, *p*). The covariance was first set to Σ=5ℐ to be greater than the covariances of the Gaussian atoms that comprised the overcomplete dictionary for primary-cortical features; these weak learners instantiated the large-covariance GMM approximation. For comparison, the covariance was set to Σ=2.5ℐ to instantiate the small-covariance GMM approximation. The covariance matrix used to summarize energy concentration of STRFs was that of the approximating distribution, $\hat{y}$, obtained from Algorithm 2.

To quantify the shape complexity of an STRF, we computed singular value decomposition of its normalized magnitude and determined how many singular values are required to account for 75% of the spectral power. This was the smallest that proportion ensured that the squared error of each *k*-rank approximation was less than 10^−3^. A larger number of singular values indicates the linear combination of more eigenmodes are required to produce the STRF, thus indicating a more complex shape.

### Spectral clustering analysis of cortical receptive fields

In order to determine whether patterns in CortRFs were unique to PEG neurons, we performed unsupervised clustering of all cortical receptive fields from both A1 and PEG neurons. A similarity matrix was constructed by computing the absolute cosine similarity between each pair of CortRFs; the diagonal components of the matrix were set to 0. The number of clusters, 6, was determined as twice the number of eigenvalues (of the normalized Laplacian associated with the similarity matrix) smaller than 0.1; this threshold corresponded approximately to the 5^th^ percentile in the distribution of the Laplacian matrix’s eigenvalues. We opted to use 6 rather than 3 clusters so that cluster representatives would be less susceptible to skew from outliers.

We performed spectral clustering using the MATLAB native function “spectralcluster”. For each cluster, we noted the number of member neurons, the proportion of PEG neurons, and computed the average cortical receptive field. Each cluster average was considered as a distinct auditory feature. If a cluster consisted of at least 50% PEG (respectively, A1) neurons, the corresponding auditory feature was interpreted to be characteristic of PEG (resp., A1). For subsequent normative comparisons, only the largest of these clusters were considered.

## Supporting information

S1 Text Contribution of spiking history. This file contains results showing that spiking history, while important, is not sufficient to obtain good statistical fits to observed responses to acoustic stimuli.
(PDF)

S2 Text Response predictivity by training stimulus and by stimulus feature space. This file contains results that compare the response predictivity of CortRFs and STRFs that were trained exclusively on either speech or TORCs.
(PDF)

S1 Fig CortRF and STRF analysis of PEG neurons. This file contains figures showing the CortRFs, STRFs, and goodness-of-fit measures of each for all PEG neurons.
(PDF)

S2 Fig CortRF and STRF analysis of A1 neurons. This file contains figures showing the CortRFs, STRFs, and goodness-of-fit measures of each for all A1 neurons.
(PDF)

S3 Fig CortRF cluster analysis. This figures show all 6 CortRF clusters.
(PDF)
