# Supplementary material for: Sparse high-dimensional decomposition of non-primary auditory cortical receptive fields
Source: PLoS Comput Biol. 2025 Jan 2;21(1):e1012721. doi: 10.1371/journal.pcbi.1012721 (PMC11774495; doi:10.1371/journal.pcbi.1012721)
Supplement: S1 Text — This file contains results showing that spiking history, while important, is not sufficient to obtain good statistical fits to observed responses to acoustic stimuli. (PDF) [file pcbi.1012721.s001.pdf]

Sparse high-dimensional decomposition of non-primary auditory cortical receptive fields

S1 Text: Contribution of spiking history

Shoutik Mukherjee[1,2], Behtash Babadi[1,2], Shihab Shamma[1,2,3] *

1 Department of Electrical and Computer Engineering, University of Maryland, College Park, Maryland, United States of America
2 Institute for Systems Research, University of Maryland, College Park, Maryland, United States of America
3 Laboratoire des Systèmes Perceptifs, Department des Études Cognitive, École Normale Supériure, Paris Sciences et Lettres University, Paris, France

*sas@umd.edu

Overview

The supporting information in this document consists of results that demonstrate the contribution of spiking-history regressors to the goodness-of-fit of estimated models of stimulus-encoding neurons. Namely, analyses of both a simulated neuron and a PEG neuron show that while important, spiking history is not sufficient to obtain good statistical fits to observed responses to acoustic stimuli.

Contribution of Spiking History

The statistical goodness-of-fit of point process models can be evaluated using the time-rescaling theorem [1], which establishes that the time-rescaled interspike intervals should be independent and distributed uniformly on the interval $(0, 1)$. Two graphical tests were employed to validate these properties [1–3]: the autocorrelation function (ACF) test is used to determine if the interspike intervals are uncorrelated; and the Kolmogorov-Smirnov test is used to determine if the time-rescaled interspike intervals are uniformly distributed. However, the time-rescaling of interspike intervals involves the estimated conditional intensity function (CIF) computed based on spiking history parameter and receptive field estimates that jointly maximized the likelihood of observed spiking; hence, distinguishing the effect of stimulus vs. spiking history requires estimating models that include only those respective regressors.

We estimated a full model including both spiking-history and stimulus dependency and one dependent only on spiking-history for a simulated neuron and a PEG neuron, comparing the goodness-of-fit of each model. The comparison for the simulated neuron is shown in Fig A, below. While the KS test of the full model indicates that the rescaled interspike intervals match a uniform distribution (Fig A–**A**), the KS test of the history-only model deviates outside of the 95% confidence interval as indicated by the blue circle (Fig A–**B**).

A similar comparison for a PEG neuron is shown in Fig B, below. As in the simulated example, the KS test of the full model indicates that the rescaled interspike

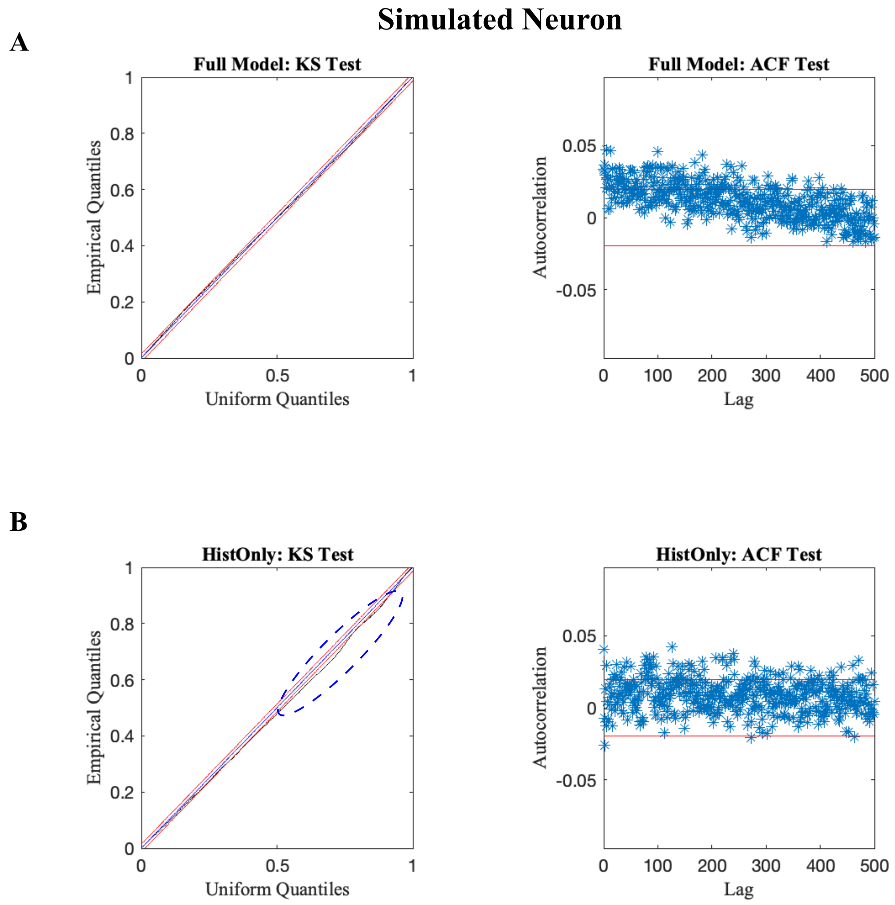

Fig A. Simulated Neuron: Full Model vs. Spiking-History Regressors. The estimated generalized linear model of a simulated neuron with spiking-history regressors only (**B**) was a poorer statistical fit to simulated data than an estimated model that also included receptive field estimation (**A**); this indicates that spiking-history regressors are useful, but not sufficient in modeling neuronal responses to stimuli. Deviations in the Kolmogorov-Smirnov tests outside the 95% confidence intervals are circled in blue.

intervals match a uniform distribution (Fig B–**A**) while the KS test of the history-only model deviates outside of the 95% confidence interval where indicated by the blue circle (Fig B–**B**).

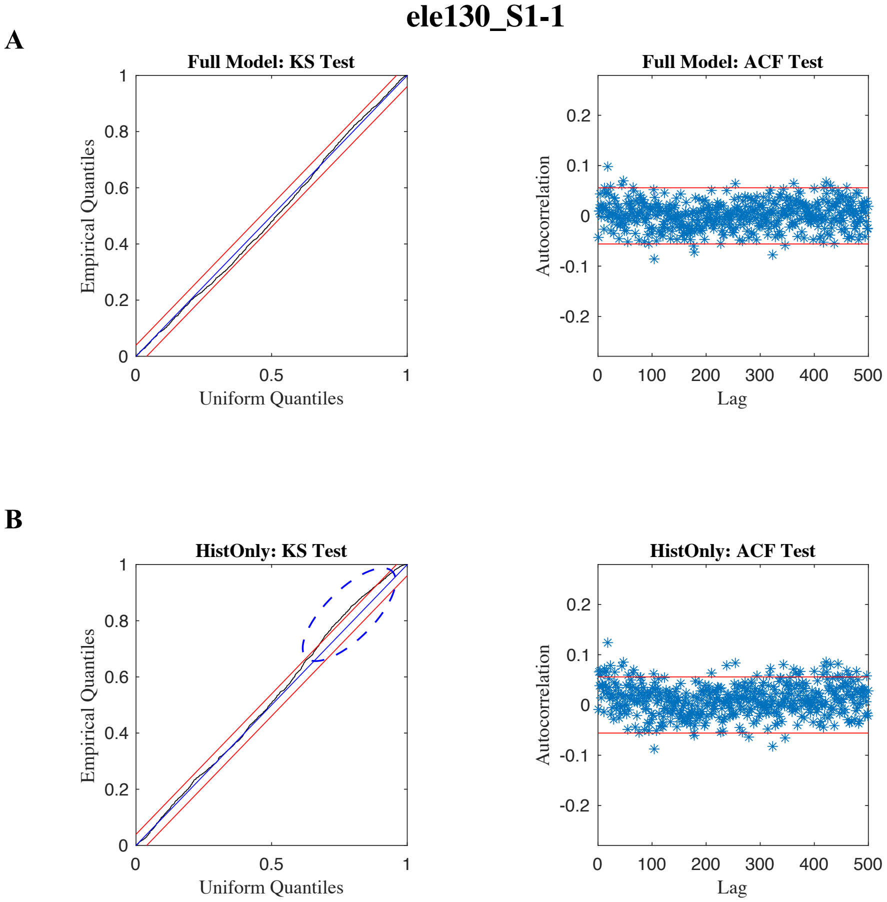

Fig B. PEG Neuron: Full Model vs. Spiking-History Regressors. The estimated generalized linear model of a PEG neuron with spiking-history regressors only (**B**) was a poorer statistical fit to observed data than an estimated model that also included receptive field estimation (**A**). Deviations in the Kolmogorov-Smirnov tests outside the 95% confidence intervals are circled in blue.

Thus, while the inclusion of spiking history regressors contributes to having statistically well-matched estimated models of responses to acoustic stimuli, full models that include receptive field estimation are better matched to observed responses, validating the relevance of receptive fields obtain by the proposed methods.

References

1. Brown EN, Barbieri R, Ventura V, Kass RE, Frank LM. The Time-Rescaling Theorem and Its Application to Neural Spike Train Data Analysis. Neural Computation. 2002;14(2):325–346. doi:10.1162/08997660252741149.

2. Truccolo W, Eden UT, Fellows MR, Donoghue JP, Brown EN. A Point Process Framework for Relating Neural Spiking Activity to Spiking History, Neural Ensemble, and Extrinsic Covariate Effects. Journal of Neurophysiology. 2005;93(2):1074–1089. doi:10.1152/jn.00697.2004.

3. Kazemipour A, Wu M, Babadi B. Robust Estimation of Self-Exciting Generalized Linear Models With Application to Neuronal Modeling. IEEE Transactions on Signal Processing. 2017;65(12):3733–3748. doi:10.1109/TSP.2017.2690385.
