# Supplementary material for: Sparse high-dimensional decomposition of non-primary auditory cortical receptive fields
Source: PLoS Comput Biol. 2025 Jan 2;21(1):e1012721. doi: 10.1371/journal.pcbi.1012721 (PMC11774495; doi:10.1371/journal.pcbi.1012721)
Supplement: S2 Text — This file contains results that compare the response predictivity of CortRFs and STRFs that were trained exclusively on either speech or TORCs. (PDF) [file pcbi.1012721.s002.pdf]

Sparse high-dimensional decomposition of non-primary auditory cortical receptive fields

S2 Text: Response predictivity by training stimulus and by stimulus feature space

Shoutik Mukherjee[1,2], Behtash Babadi[1,2], Shihab Shamma[1,2,3] *

1 Department of Electrical and Computer Engineering, University of Maryland, College Park, Maryland, United States of America
2 Institute for Systems Research, University of Maryland, College Park, Maryland, United States of America
3 Laboratoire des Systèmes Perceptifs, Department des Études Cognitive, École Normale Supériure, Paris Sciences et Lettres University, Paris, France

*sas@umd.edu

Overview

The supporting information in this document consists of results that summarize how the choice of training stimulus and choice of stimulus feature space affect response predictivity. The comparisons here further support our results indicating the primary-cortical model captured aspects of a feature transformation that specifically helped encode speech.

Recall from Fig 5 that in PEG neurons, CortRFs trained on both TORC and speech responses enabled better predictions of speech responses than similarly trained STRFs; while, from Fig 6**D**, no such difference was found in A1 neurons. This suggested a transformation of stimulus representations between A1 and PEG, captured in part by the primary-cortical feature space, that facilitates encoding natural acoustic stimuli. Here, we elaborate on this hypothesis by first investigating if there may be speech-specific features not captured by receptive fields unless speech responses are used in their estimation, then examining if the primary-cortical feature space was beneficial in capturing speech-specific features.

We estimated CortRFs and STRFs of both PEG and A1 neurons, using either TORC or speech responses exclusively, and compared the performance of these models in predicting responses to TORC and speech. Restricting the responses used for estimating receptive fields imposes a strong prior on the distribution of stimulus features. Hence, comparing the predictivity of TORC-trained vs. speech-trained receptive field models serves as a measure of difference between these priors. On the other hand, comparing the predictivity of CortRFs vs. STRFs trained on the same type of stimulus indicates how well the primary-cortical model accounts for these differences.

A summary of the findings detailed below are as follows: (1) Training with speech stimuli offered better predictions of speech responses in both A1 and PEG. No such advantage was gained when training with TORCs, suggesting that speech signals contain features that both STRFs and CortRFs can only attain when trained on speech. (2) When trained on the general TORC stimuli, the more complex CortRFs outperform

STRFs in predicting all responses in both A1 and PEG. This implies that including the multiscale primary-cortical ripple-decomposition endows the CortRF model with speech-specific features that are absent in the TORC stimuli alone. This finding is further confirmed by the loss of the CortRF model's advantage when training with speech, presumably because speech-specific features are then acquired by both STRFs and CortRFs and hence the advantage of the multiscale primary-cortical decomposition disappears.

TORC-trained vs. speech-trained receptive fields

We first compared how predictive of TORC and speech responses CortRFs and STRFs were when estimated from only TORC responses vs. on speech responses. Predictivity was quantified by the cosine similarity between observed spiking and the estimated CIF, just as in our main results (Fig 5, 6**D**). The differences in predictivity of TORC-trained vs. speech-trained receptive fields are illustrated in Fig A.

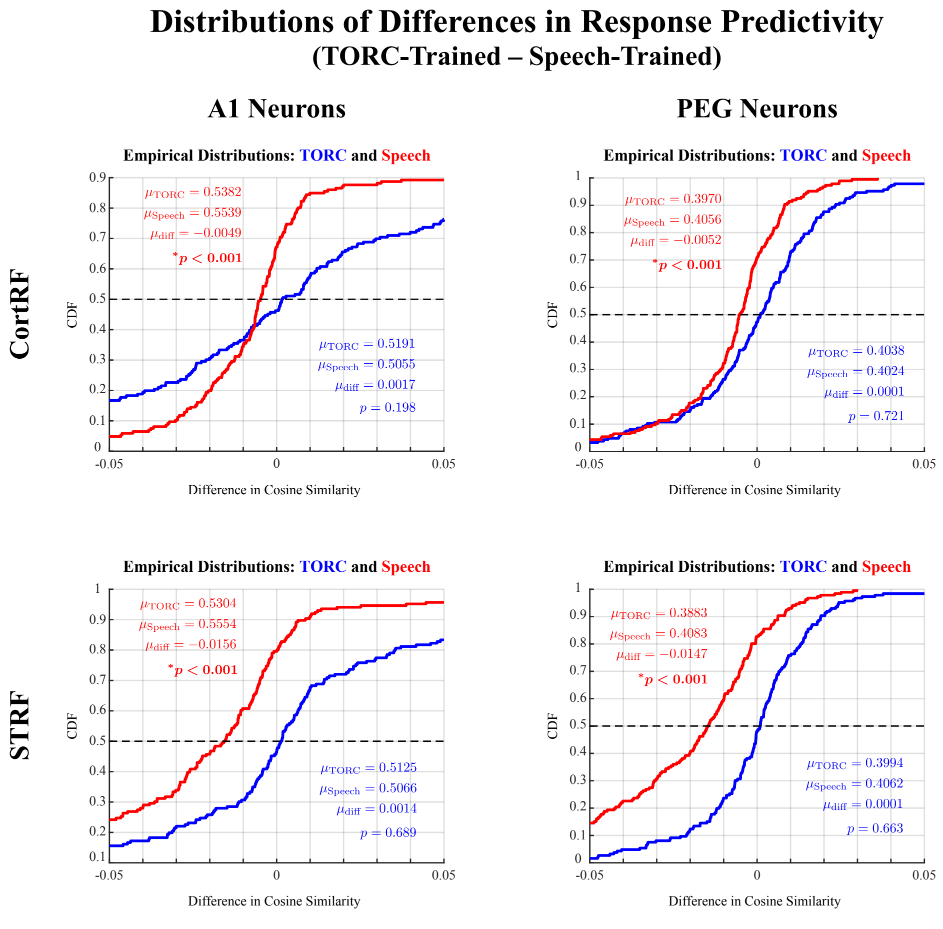

Fig A. Speech-Trained vs. TORC-Trained Receptive Fields. Empirical CDFs of the differences between TORC-trained and speech-trained receptive fields in predictivity of responses to TORC stimuli (blue) and speech stimuli (red) for A1 neurons (left column) and PEG neurons (right column). Distributions of differences were compiled for CortRF models (top row) and STRF models (bottom row). Significant non-zero median differences (μ_{diff}) were determined using the Wilcoxon signed rank test at with significance level $p < 0.05$.

Spiking responses to speech stimuli in both A1 and PEG were better predicted when receptive fields (both CortRFs and STRFs) are estimated from speech responses than when estimated from TORC responses, but no such differences were present in predicting TORC responses. This suggests there are features of speech that receptive fields can only capture when trained on responses to speech stimuli, irrespective of cortical area or stimulus feature space.

CortRF vs. STRF

We next compared CortRFs and STRFs, trained on the same type of stimulus, by their predictivity of TORC and speech responses, as illustrated in Fig B. For both PEG and A1 neurons, the predictive differences between CortRFs and STRFs trained on speech responses were statistically significant; STRFs trained on speech outperformed CortRFs trained on speech in predicting responses of PEG and A1 neurons to speech stimuli as well as responses to stimuli. Significant differences in predictivity were also found when CortRFs and STRFs were trained on TORC responses. In both PEG and A1, TORC-trained CortRFs outperformed STRFs in predicting responses to speech stimuli; however, in PEG neurons, STRFs were more predictive of TORC responses than CortRFs while being less predictive of TORC responses in A1 neurons. Notably, the difference is larger for speech response predictivity than TORC response predictivity.

The difference between TORC-trained CortRFs and STRFs in predicting speech responses, in conjunction with the differences observed in Fig A, suggests that speech-specific features can be at least partially accounted for in the primary-cortical feature space despite the absence of speech responses when estimating the CortRF. However, the differences shown in Fig A also suggest that some features of speech can only be capture by training on speech responses. This is corroborated by the observation in Fig B that STRFs trained on speech responses were sufficient to predict responses to both TORC and speech stimuli at least as well as CortRFs.

In fact, the STRFs trained on speech responses outperformed CortRFs. We speculate that this may reflect overfitting. CortRF estimation utilizes the primary-cortical feature space, which is more complex than the spectrogram representation and better captures speech-specific features. Hence, the CortSTRFs derived from speech-trained CortRFs may be excessively more complex than speech-trained STRFs. Consequently, they are less generalizable for predicting responses to TORCs or novel speech stimuli than STRFs. This emphasizes that diverse stimuli are required to estimate generablizable receptive fields, further justifying the use of CortRFs and STRFs estimated using both speech and TORC responses in our main results to compare the feature selectivity of PEG and A1 neurons.

This is further illustrated in Figure C, below. The neural representation of speech spectrogram (Fig C–**A**) was computed via 2-dimensional convolutions with the average CortRFs, trained either only on speech stimuli or on both TORC and speech, of PEG or A1 neurons. It is evident via inspection that when CortRFs were trained on both TORC and speech stimuli, the average speech representation in PEG (Fig C–**B**) is more complex than in A1 (Fig C–**C**), corroborating the results presented in the main text. In contrast, when trained on speech stimuli alone, the PEG representation and A1 representation are both simpler (Fig C–**D** vs. Fig C–**B** and Fig C–**E** vs. Fig C–**C**, respectively) and in fact comparable to one another (Fig C–**D** vs. Fig C–**E**).

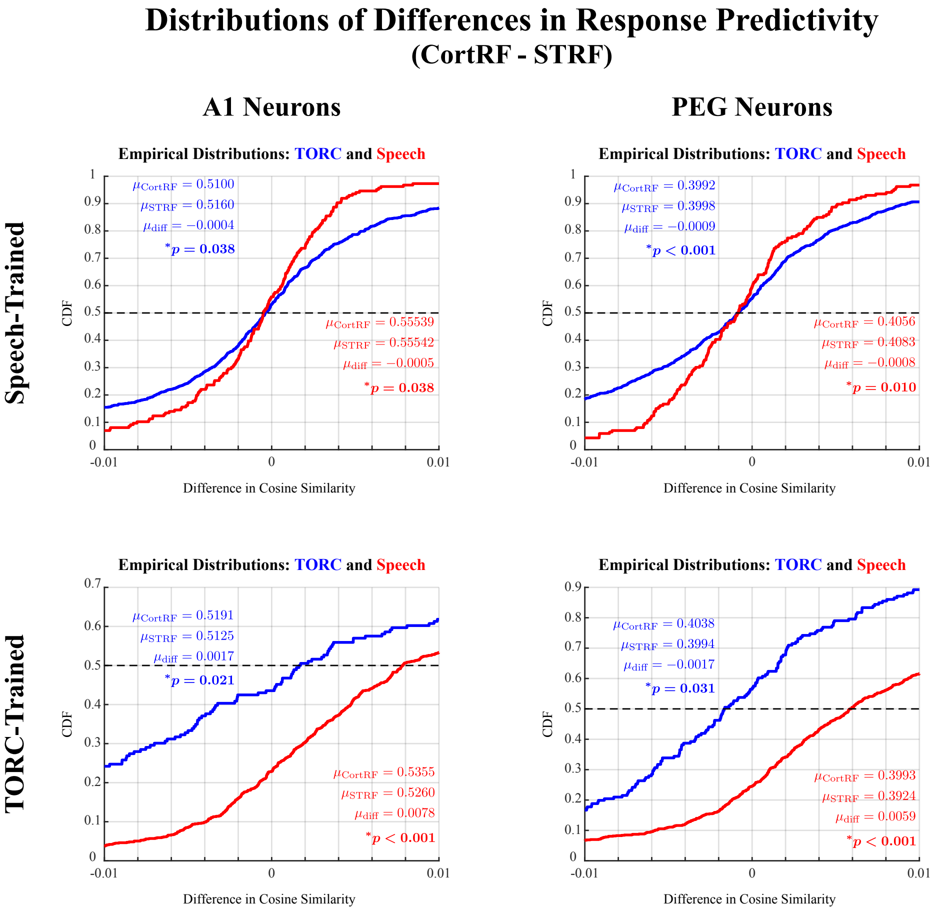

Fig B. CortRFs vs. STRFs. Empirical CDFs of the differences between CortRF and STRFs in predictivity of responses to TORC stimuli (blue) and speech stimuli (red) for A1 neurons (left column) and PEG neurons (right column). Distributions of differences were compiled for speech-trained models (top row) and TORC-trained models (bottom row). Significant non-zero median differences (μ_{diff}) were determined using the Wilcoxon signed rank test at with significance level $p < 0.05$.

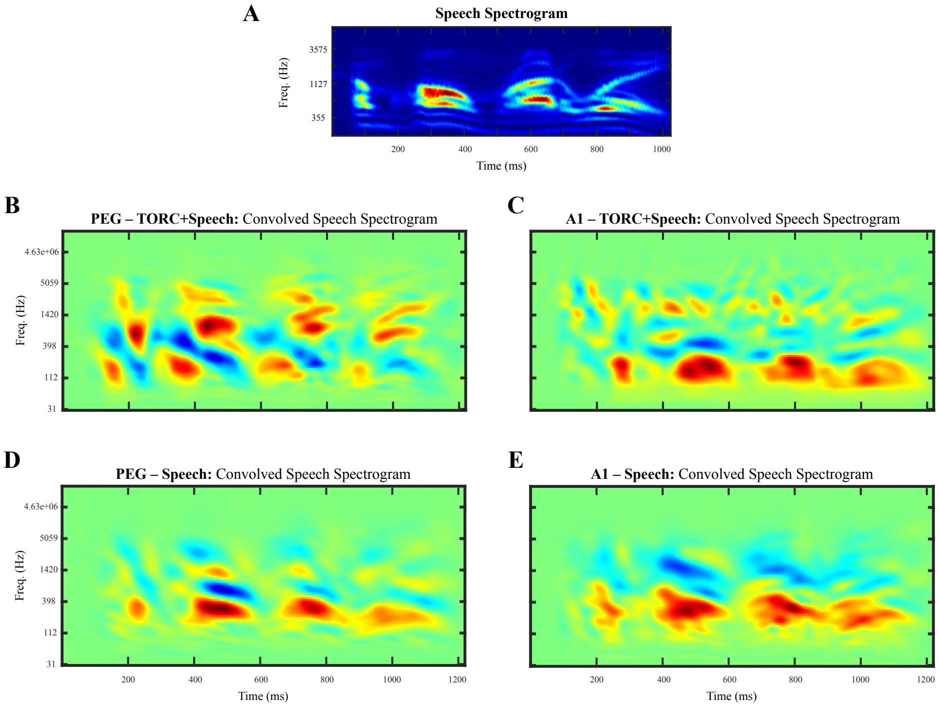

Fig C. Convolved Speech Spectrograms: PEG vs. A1. The neural representations of a speech spectrogram (**A**) computed via 2-dimensional convolutions with the average CortRF of PEG neurons trained on TORC and Speech (**B**); the average CortRF of A1 neurons trained on TORC and Speech (**C**); the average CortRF of PEG neurons trained only on Speech (**D**); and the average CortRF of A1 neurons trained only on Speech stimuli (**E**).
