# Supplementary material for: Sparse high-dimensional decomposition of non-primary auditory cortical receptive fields
Source: PLoS Comput Biol. 2025 Jan 2;21(1):e1012721. doi: 10.1371/journal.pcbi.1012721 (PMC11774495; doi:10.1371/journal.pcbi.1012721)
Supplement: S2 Fig — This file contains figures showing the CortRFs, STRFs, and goodness-of-fit measures of each for all A1 neurons. (PDF) [file pcbi.1012721.s004.pdf]

Sparse high-dimensional decomposition of non-primary auditory cortical receptive fields

S2 Figures: CortRF and STRF analysis of A1 neurons

Shoutik Mukherjee[1,2], Behtash Babadi[1,2], Shihab Shamma[1,2,3] *

1 Department of Electrical and Computer Engineering, University of Maryland, College Park, Maryland, United States of America
2 Institute for Systems Research, University of Maryland, College Park, Maryland, United States of America
3 Laboratoire des Systèmes Perceptifs, Department des Études Cognitive, École Normale Supériure, Paris Sciences et Lettres University, Paris, France

*sas@umd.edu

Overview

This supporting information file includes the estimated CortRF and STRF for all A1 neurons, and goodness-of-fit measures of each. The figure layout is shown in Fig. S2.1.

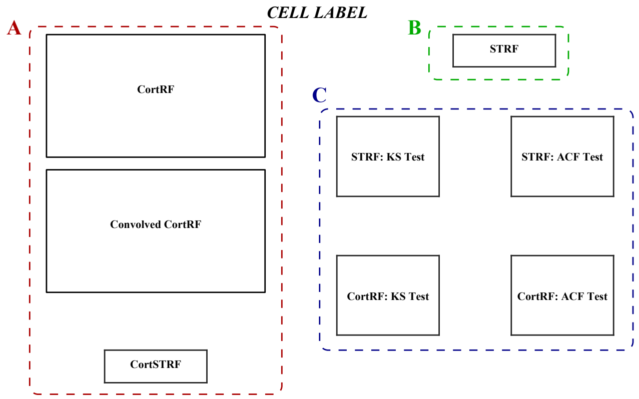

Fig S2.1. Estimated receptive fields and goodness-of-fit measures. **A.** The CortRF, estimated as a sparse combination of atoms from a dictionary of truncated Gaussian kernels (top). Its convolution with the primary-cortical basis functions (middle) shows the rates and scales to which the neuron is responsive. The CortSTRF marignalizes the convolved CortRF over rates and scales, describing the spectrotemporal features to which the neuron is responsive. **B.** The estimated STRF of the neuron. **C.** Goodness-of-fit measures showing both the STRF (top row) and CortRF (botton row) produce models that are statistically well-matched to observed spiking responses.

ele053_S1-1

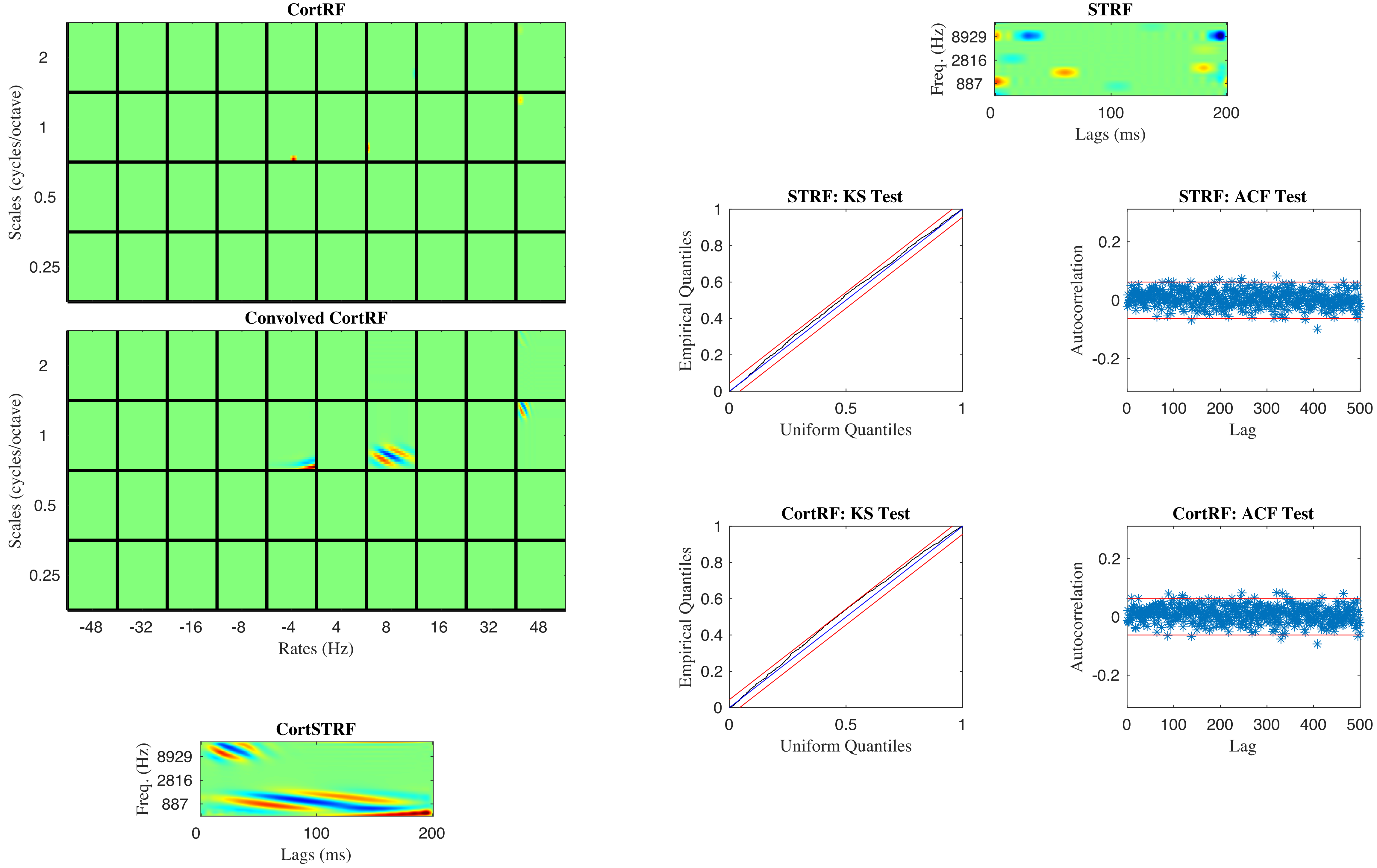
CortRF
Scales (cycles/octave)
2
1
0.5
0.25
Convolved CortRF
Scales (cycles/octave)
2
1
0.5
0.25
-48 -32 -16 -8 -4 4 8 16 32 48
Rates (Hz)
CortSTRF
Freq. (Hz)
8929
2816
887
0 100 200
Lags (ms)
STRF
Freq. (Hz)
8929
2816
887
0 100 200
Lags (ms)
STRF: KS Test
Empirical Quantiles
0 0.2 0.4 0.6 0.8 1
0 0.5 1
Uniform Quantiles
STRF: ACF Test
Autocorrelation
0.2 0 -0.2
0 100 200 300 400 500
Lag
CortRF: KS Test
Empirical Quantiles
0 0.2 0.4 0.6 0.8 1
0 0.5 1
Uniform Quantiles
CortRF: ACF Test
Autocorrelation
0.2 0 -0.2
0 100 200 300 400 500
Lag

ele053_S2-1

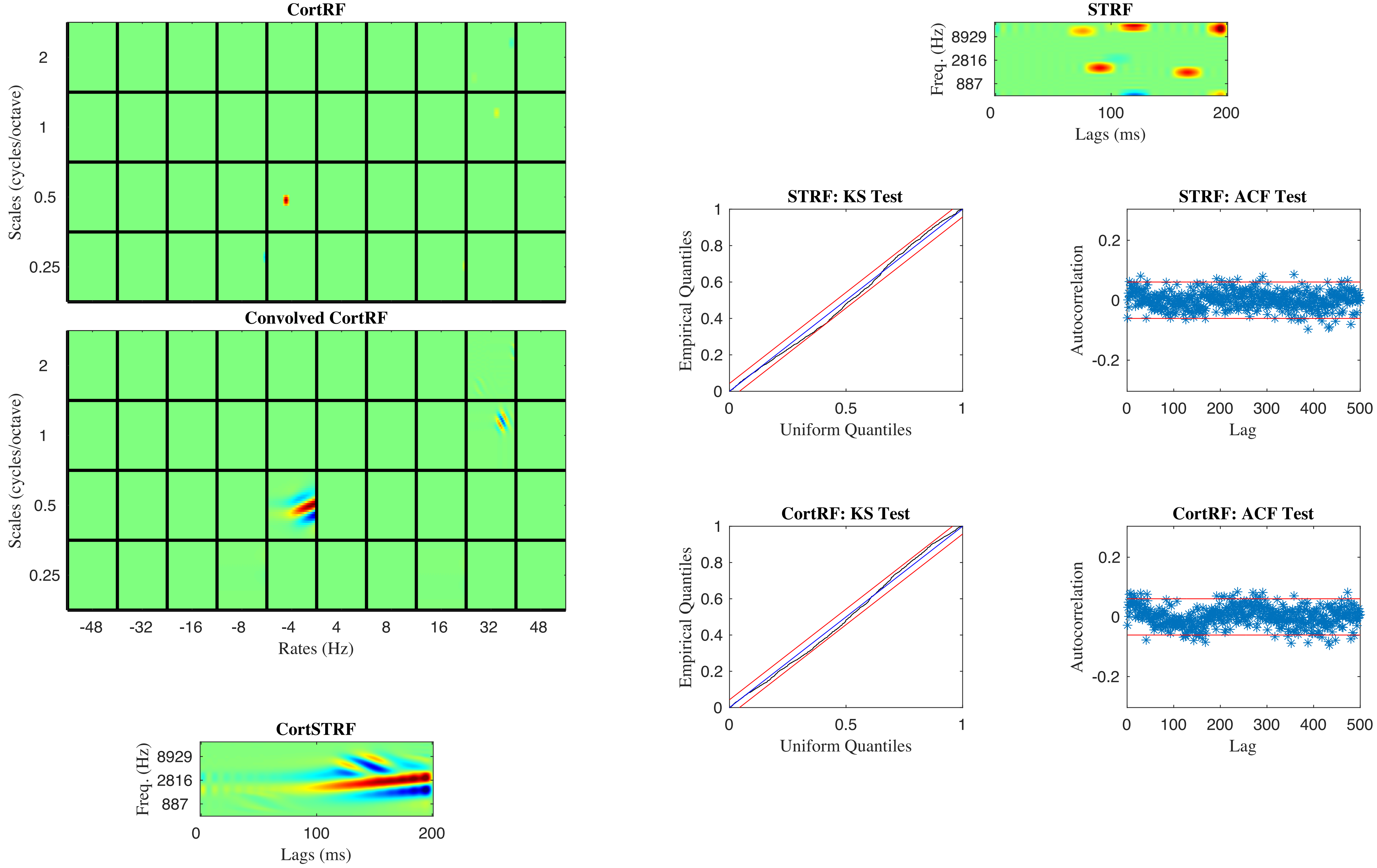

CortRF
Scales (cycles/octave)
2
1
0.5
0.25
Convolved CortRF
Scales (cycles/octave)
2
1
0.5
0.25
-48 -32 -16 -8 -4 4 8 16 32 48
Rates (Hz)
CortSTRF
Freq. (Hz)
8929
2816
887
0 100 200
Lags (ms)
STRF
Freq. (Hz)
8929
2816
887
0 100 200
Lags (ms)
STRF: KS Test
Empirical Quantiles
Uniform Quantiles
STRF: ACF Test
Autocorrelation
Lag
CortRF: KS Test
Empirical Quantiles
Uniform Quantiles
CortRF: ACF Test
Autocorrelation
Lag

ele055_S2-1

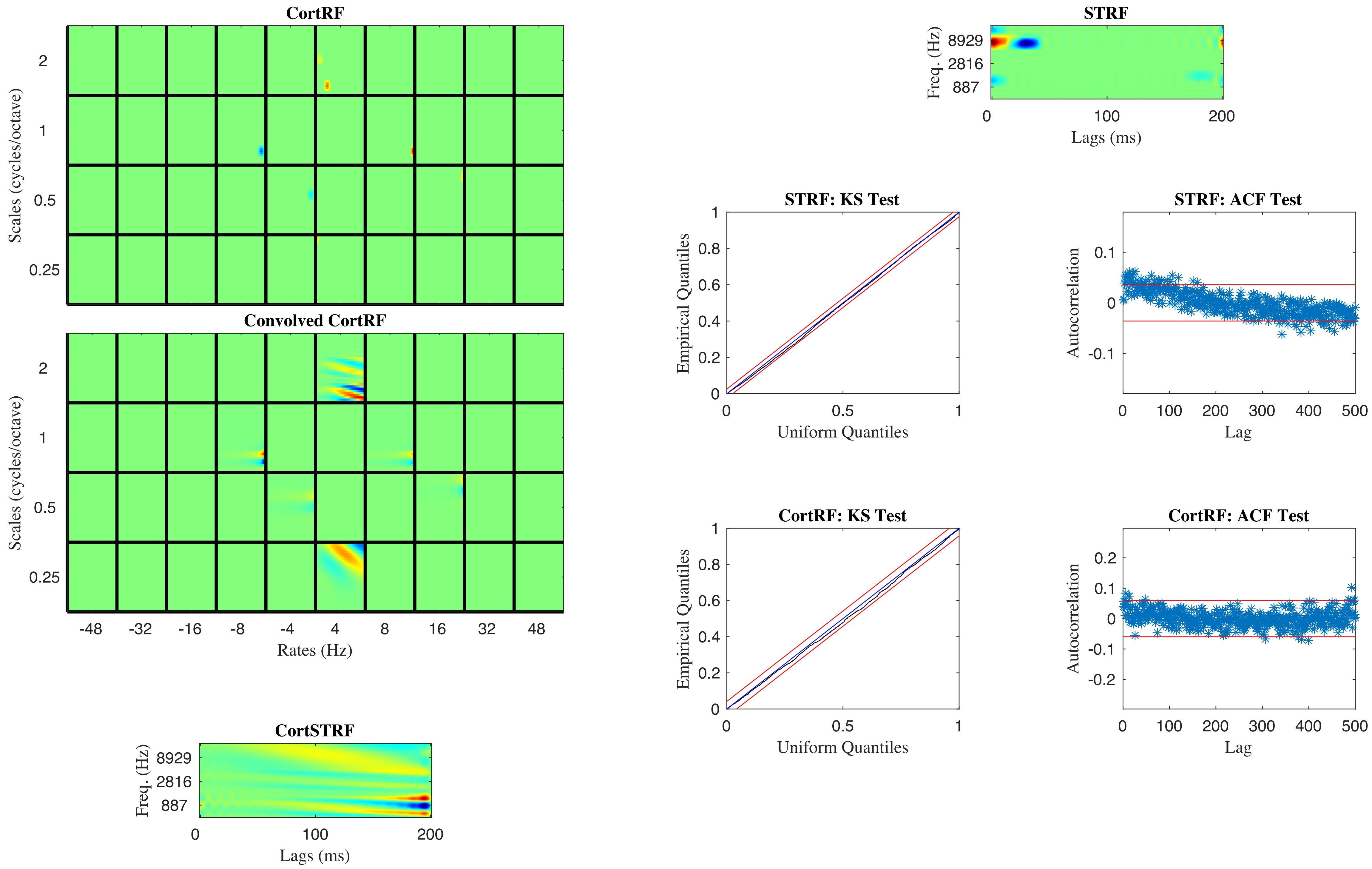
CortRF
Scales (cycles/octave)
2
1
0.5
0.25
Convolved CortRF
Scales (cycles/octave)
2
1
0.5
0.25
-48 -32 -16 -8 -4 4 8 16 32 48
Rates (Hz)
CortSTRF
Freq. (Hz)
8929
2816
887
0 100 200
Lags (ms)
STRF
Freq. (Hz)
8929
2816
887
0 100 200
Lags (ms)
STRF: KS Test
Empirical Quantiles
0 0.2 0.4 0.6 0.8 1
0 0.5 1
Uniform Quantiles
STRF: ACF Test
Autocorrelation
0.1 0 -0.1
0 100 200 300 400 500
Lag
CortRF: KS Test
Empirical Quantiles
0 0.2 0.4 0.6 0.8 1
0 0.5 1
Uniform Quantiles
CortRF: ACF Test
Autocorrelation
0.2 0.1 0 -0.1 -0.2
0 100 200 300 400 500
Lag

ele056_S1-2

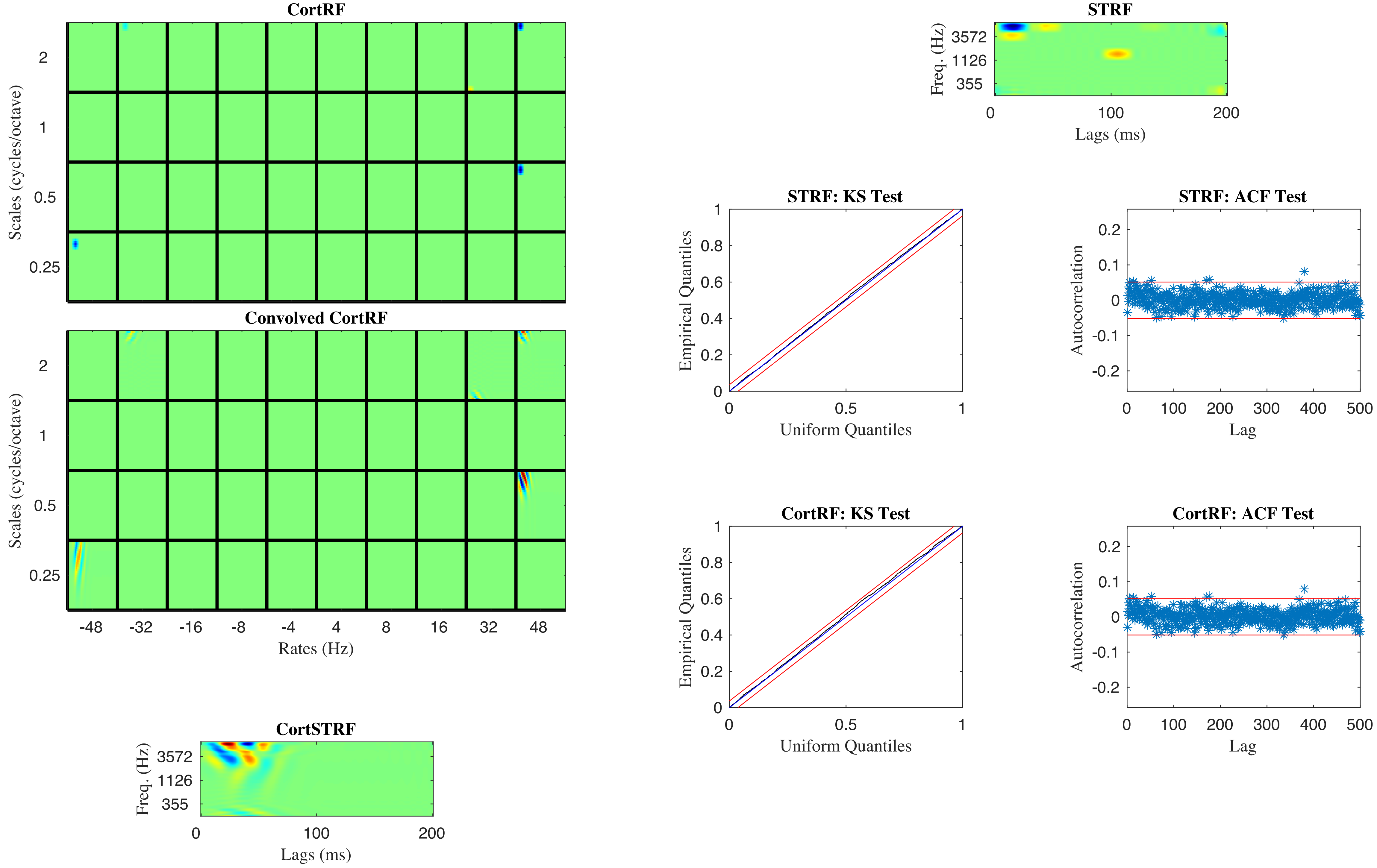

CortRF
Scales (cycles/octave)
2
1
0.5
0.25
Convolved CortRF
Scales (cycles/octave)
2
1
0.5
0.25
-48 -32 -16 -8 -4 4 8 16 32 48
Rates (Hz)
CortSTRF
Freq. (Hz)
3572
1126
355
0 100 200
Lags (ms)
STRF
Freq. (Hz)
3572
1126
355
0 100 200
Lags (ms)
STRF: KS Test
Empirical Quantiles
Uniform Quantiles
STRF: ACF Test
Autocorrelation
Lag
CortRF: KS Test
Empirical Quantiles
Uniform Quantiles
CortRF: ACF Test
Autocorrelation
Lag

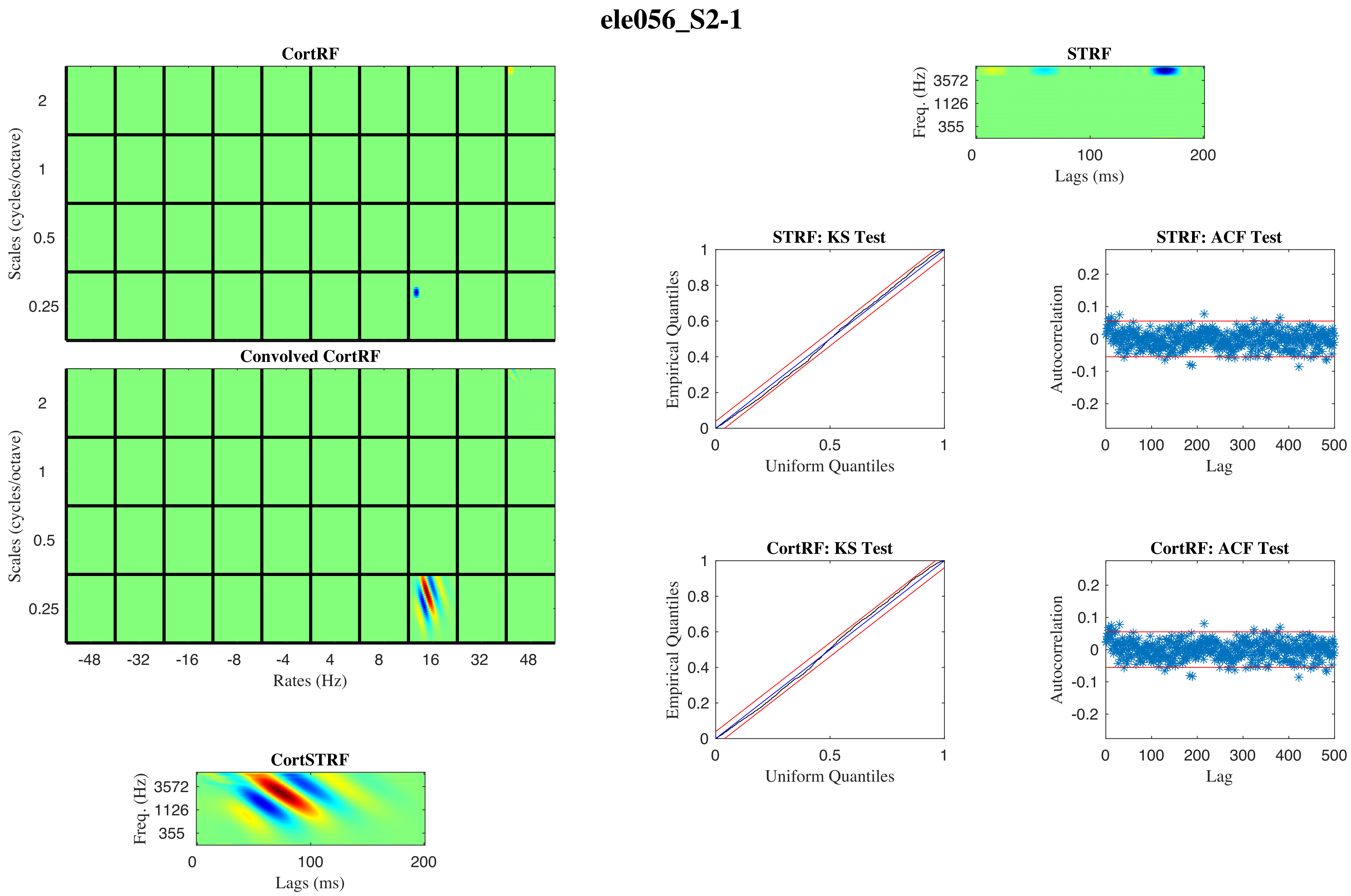

ele056_S2-1
CortRF
Scales (cycles/octave)
2
1
0.5
0.25
Convolved CortRF
-48
-32
-16
-8
-4
4
8
16
32
48
Rates (Hz)
CortSTRF
Freq. (Hz)
3572
1126
355
0
100
200
Lags (ms)
STRF
STRF: KS Test
Empirical Quantiles
Uniform Quantiles
STRF: ACF Test
Autocorrelation
Lag
CortRF: KS Test
CortRF: ACF Test

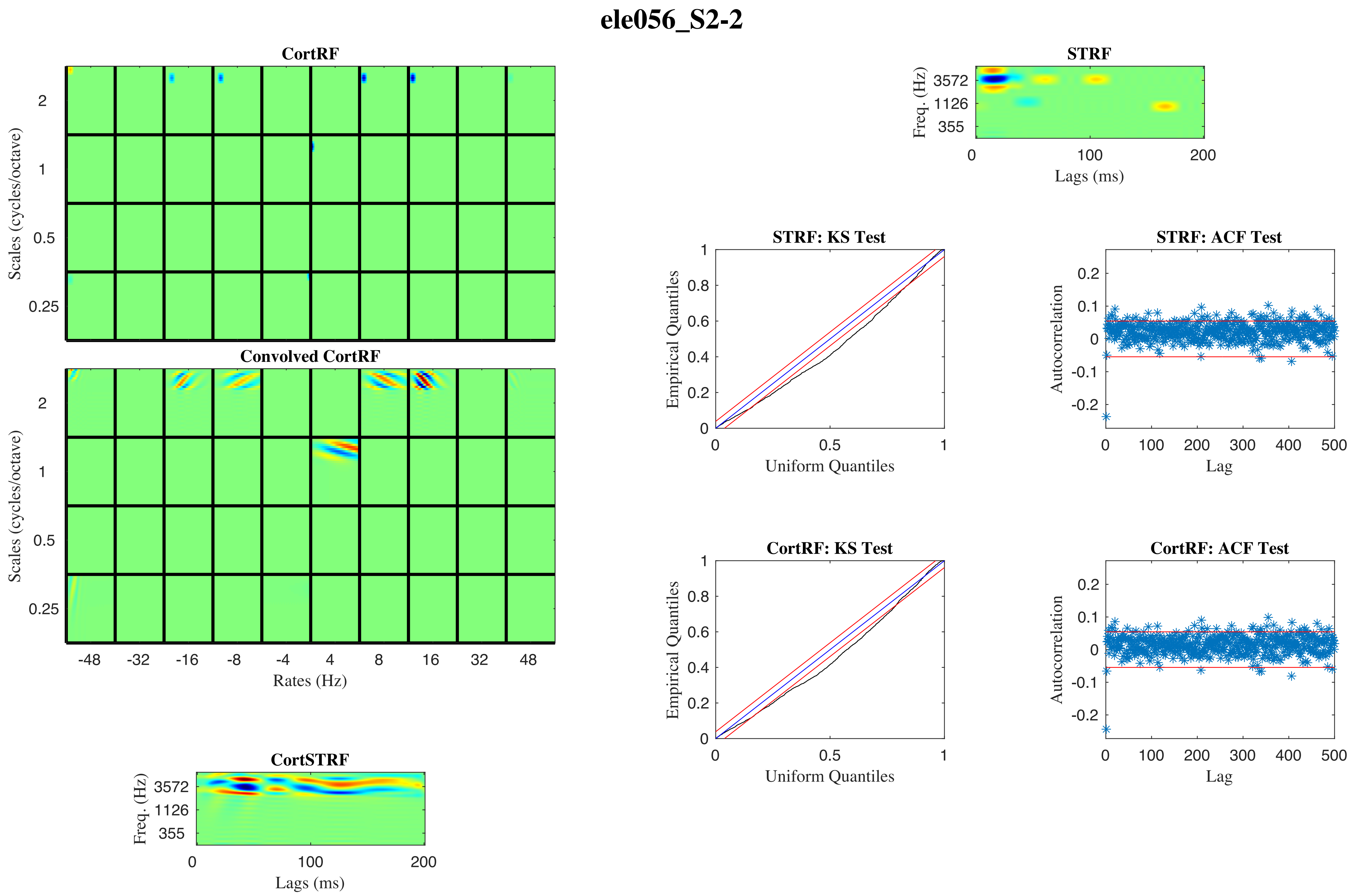

ele056_S2-2
CortRF
Scales (cycles/octave)
2
1
0.5
0.25
Convolved CortRF
Rates (Hz)
-48
-32
-16
-8
-4
4
8
16
32
48
CortSTRF
Freq. (Hz)
3572
1126
355
Lags (ms)
0
100
200
STRF
STRF: KS Test
Empirical Quantiles
Uniform Quantiles
STRF: ACF Test
Autocorrelation
Lag
CortRF: KS Test
CortRF: ACF Test

ele056_S3-1

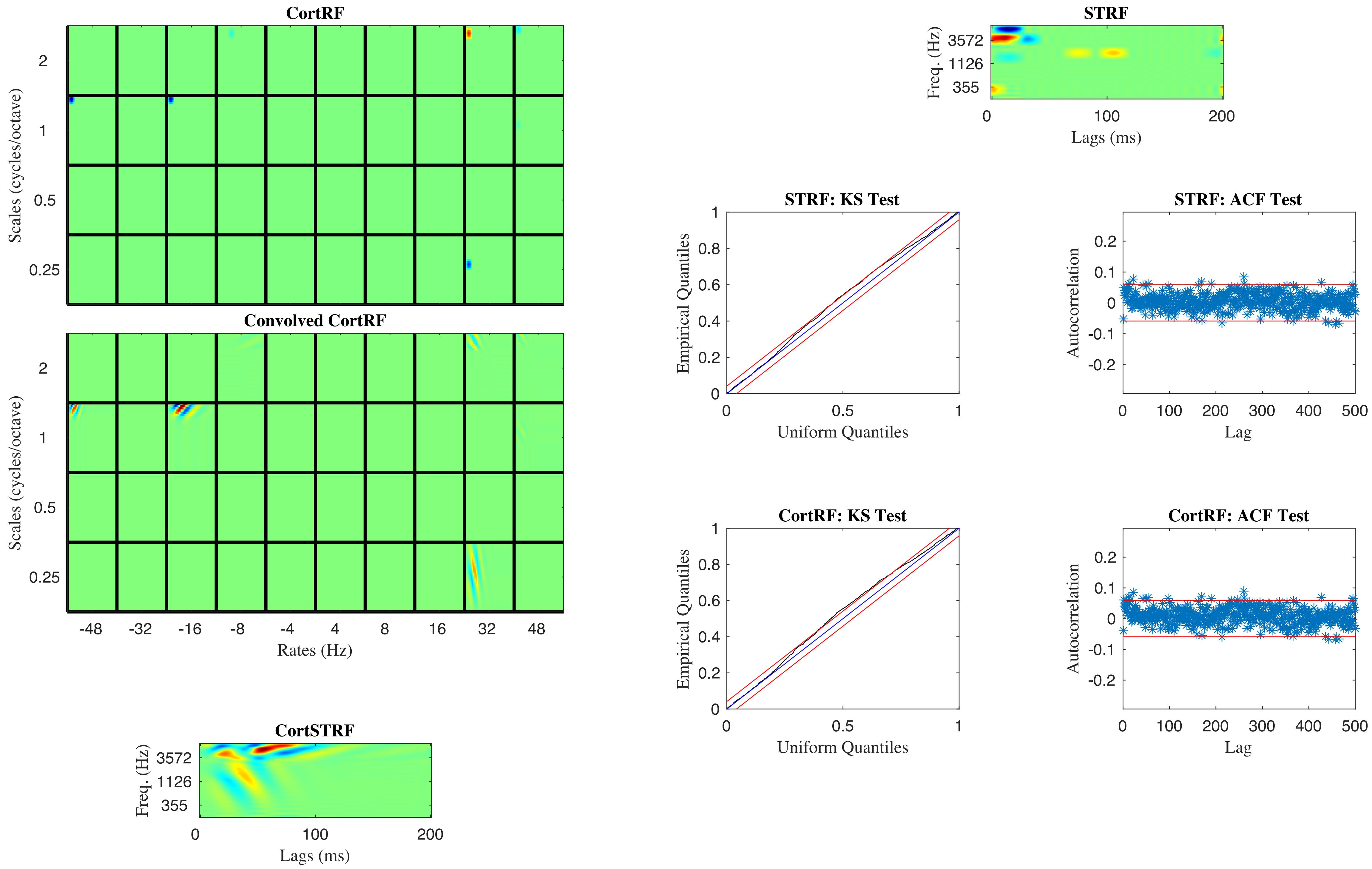

CortRF
Scales (cycles/octave)
2
1
0.5
0.25
Convolved CortRF
Scales (cycles/octave)
2
1
0.5
0.25
-48
-32
-16
-8
-4
4
8
16
32
48
Rates (Hz)
CortSTRF
Freq. (Hz)
3572
1126
355
0
100
200
Lags (ms)
STRF
Freq. (Hz)
3572
1126
355
0
100
200
Lags (ms)
STRF: KS Test
Empirical Quantiles
Uniform Quantiles
STRF: ACF Test
Autocorrelation
Lag
CortRF: KS Test
Empirical Quantiles
Uniform Quantiles
CortRF: ACF Test
Autocorrelation
Lag

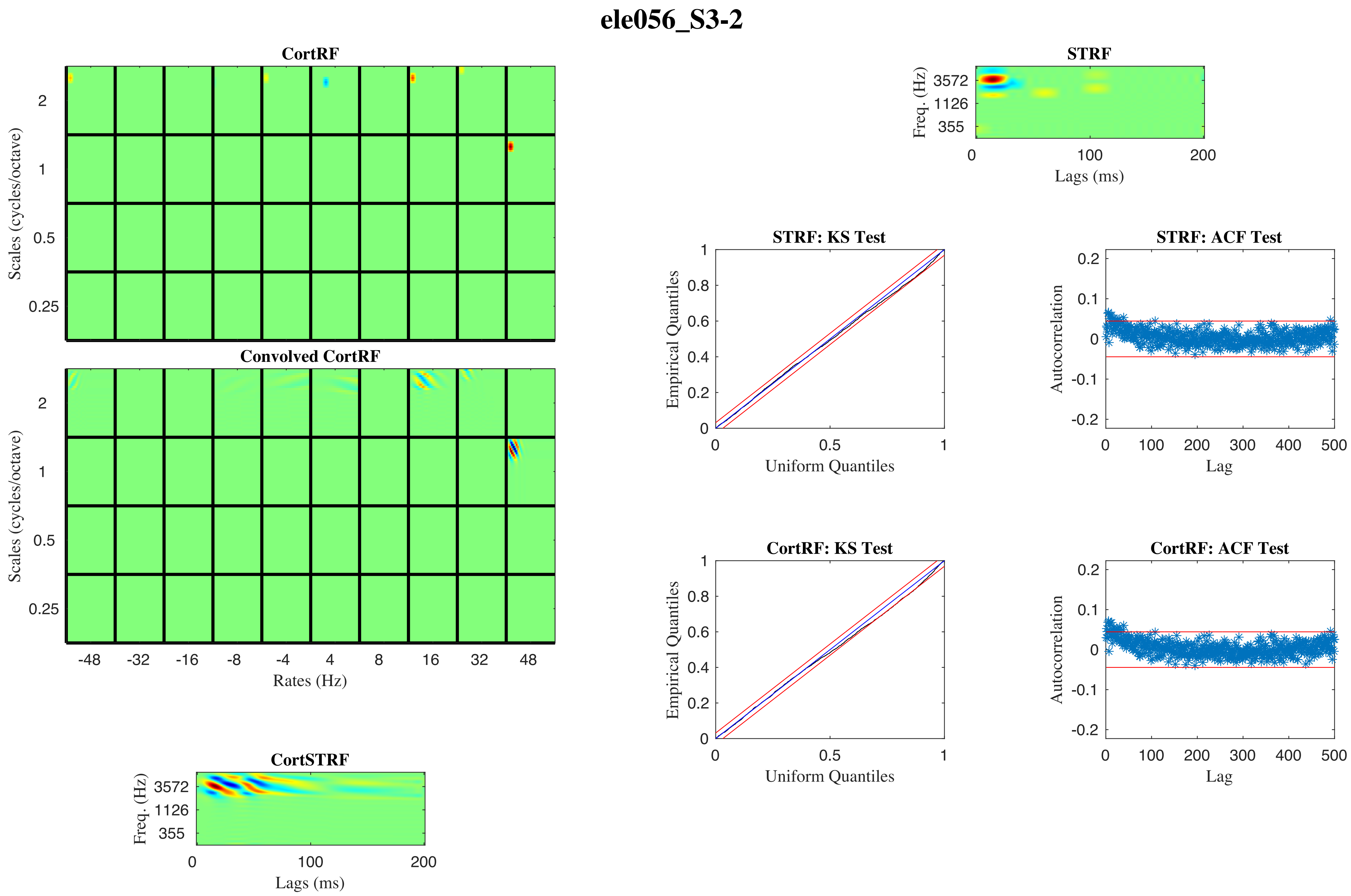
ele056_S3-2
CortRF
Scales (cycles/octave)
2
1
0.5
0.25
Convolved CortRF
Scales (cycles/octave)
2
1
0.5
0.25
-48 -32 -16 -8 -4 4 8 16 32 48
Rates (Hz)
CortSTRF
Freq. (Hz)
3572
1126
355
0 100 200
Lags (ms)
STRF
Freq. (Hz)
3572
1126
355
0 100 200
Lags (ms)
STRF: KS Test
Empirical Quantiles
0 0.2 0.4 0.6 0.8 1
0 0.5 1
Uniform Quantiles
STRF: ACF Test
Autocorrelation
0.2 0.1 0 -0.1 -0.2
0 100 200 300 400 500
Lag
CortRF: KS Test
Empirical Quantiles
0 0.2 0.4 0.6 0.8 1
0 0.5 1
Uniform Quantiles
CortRF: ACF Test
Autocorrelation
0.2 0.1 0 -0.1 -0.2
0 100 200 300 400 500
Lag

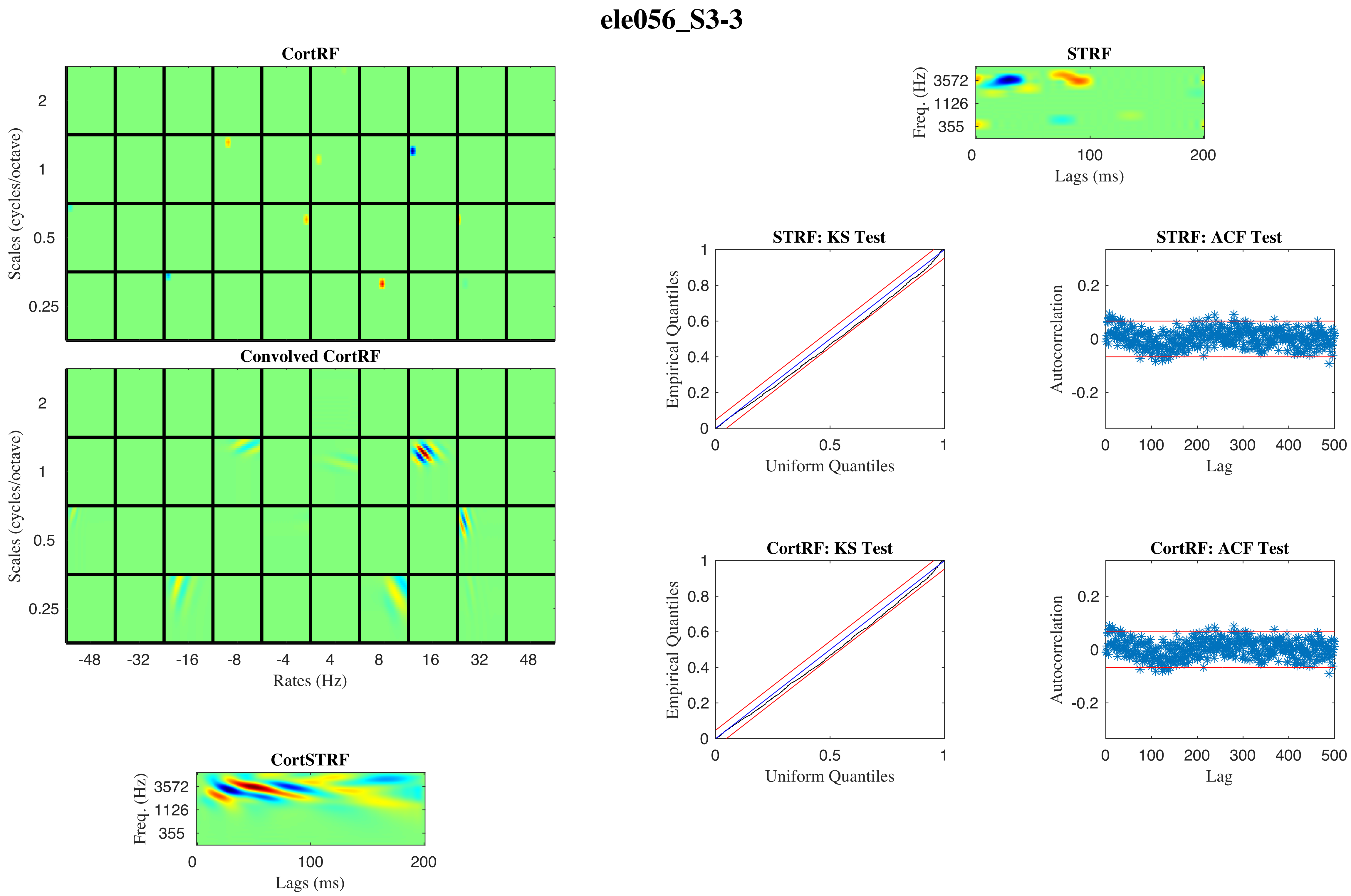
ele056_S3-3
CortRF
Scales (cycles/octave)
2
1
0.5
0.25
Convolved CortRF
Scales (cycles/octave)
2
1
0.5
0.25
-48 -32 -16 -8 -4 4 8 16 32 48
Rates (Hz)
CortSTRF
Freq. (Hz)
3572
1126
355
0 100 200
Lags (ms)
STRF
Freq. (Hz)
3572
1126
355
0 100 200
Lags (ms)
STRF: KS Test
Empirical Quantiles
0 0.2 0.4 0.6 0.8 1
0 0.5 1
Uniform Quantiles
STRF: ACF Test
Autocorrelation
0.2 0 -0.2
0 100 200 300 400 500
Lag
CortRF: KS Test
Empirical Quantiles
0 0.2 0.4 0.6 0.8 1
0 0.5 1
Uniform Quantiles
CortRF: ACF Test
Autocorrelation
0.2 0 -0.2
0 100 200 300 400 500
Lag

ele057_S1-1

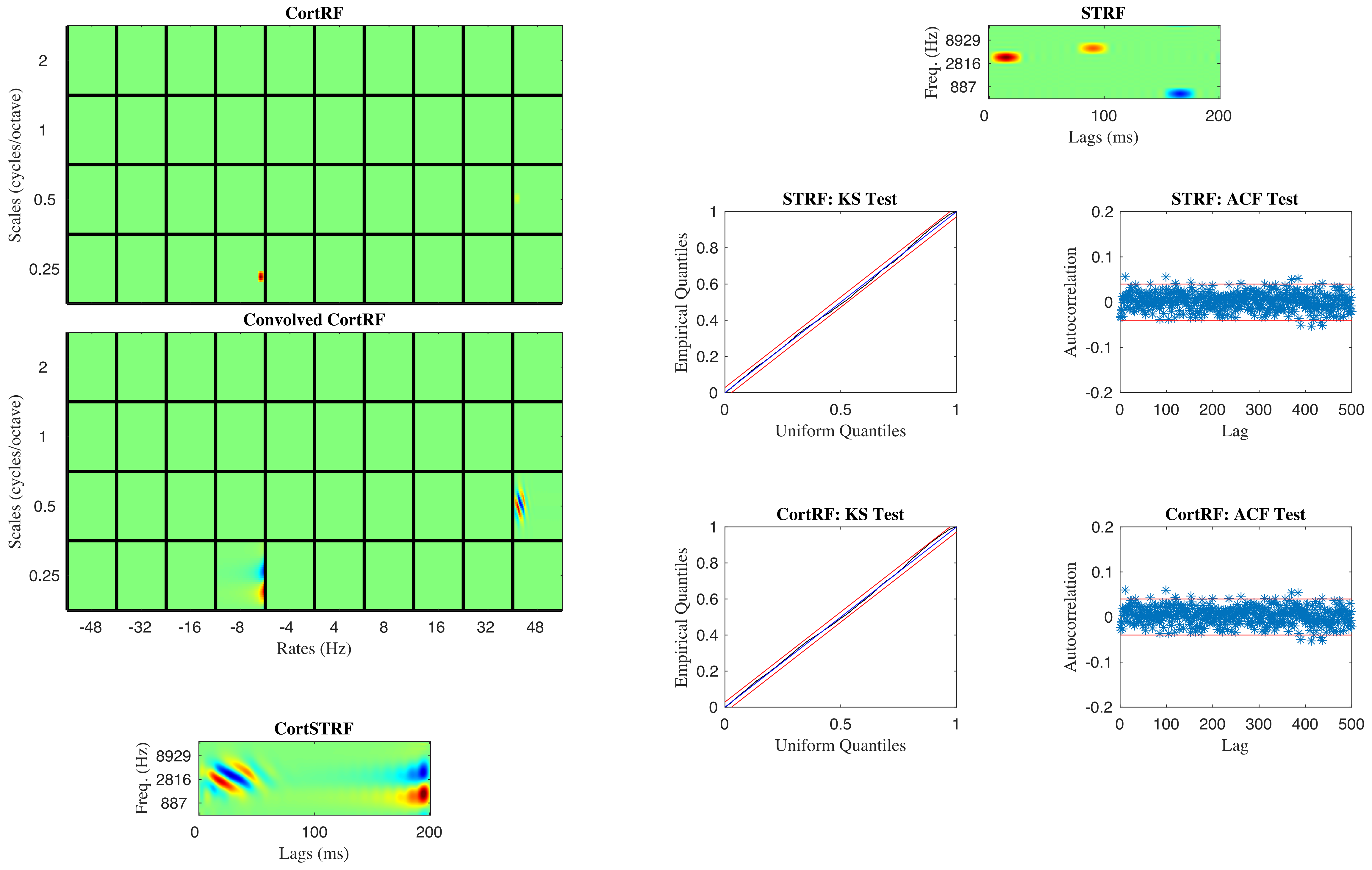
CortRF
Scales (cycles/octave)
2
1
0.5
0.25
Convolved CortRF
Scales (cycles/octave)
2
1
0.5
0.25
-48 -32 -16 -8 -4 4 8 16 32 48
Rates (Hz)
CortSTRF
Freq. (Hz)
8929
2816
887
0 100 200
Lags (ms)
STRF
Freq. (Hz)
8929
2816
887
0 100 200
Lags (ms)
STRF: KS Test
Empirical Quantiles
0 0.2 0.4 0.6 0.8 1
0 0.5 1
Uniform Quantiles
STRF: ACF Test
Autocorrelation
-0.2 -0.1 0 0.1 0.2
0 100 200 300 400 500
Lag
CortRF: KS Test
Empirical Quantiles
0 0.2 0.4 0.6 0.8 1
0 0.5 1
Uniform Quantiles
CortRF: ACF Test
Autocorrelation
-0.2 -0.1 0 0.1 0.2
0 100 200 300 400 500
Lag

ele057_S2-1

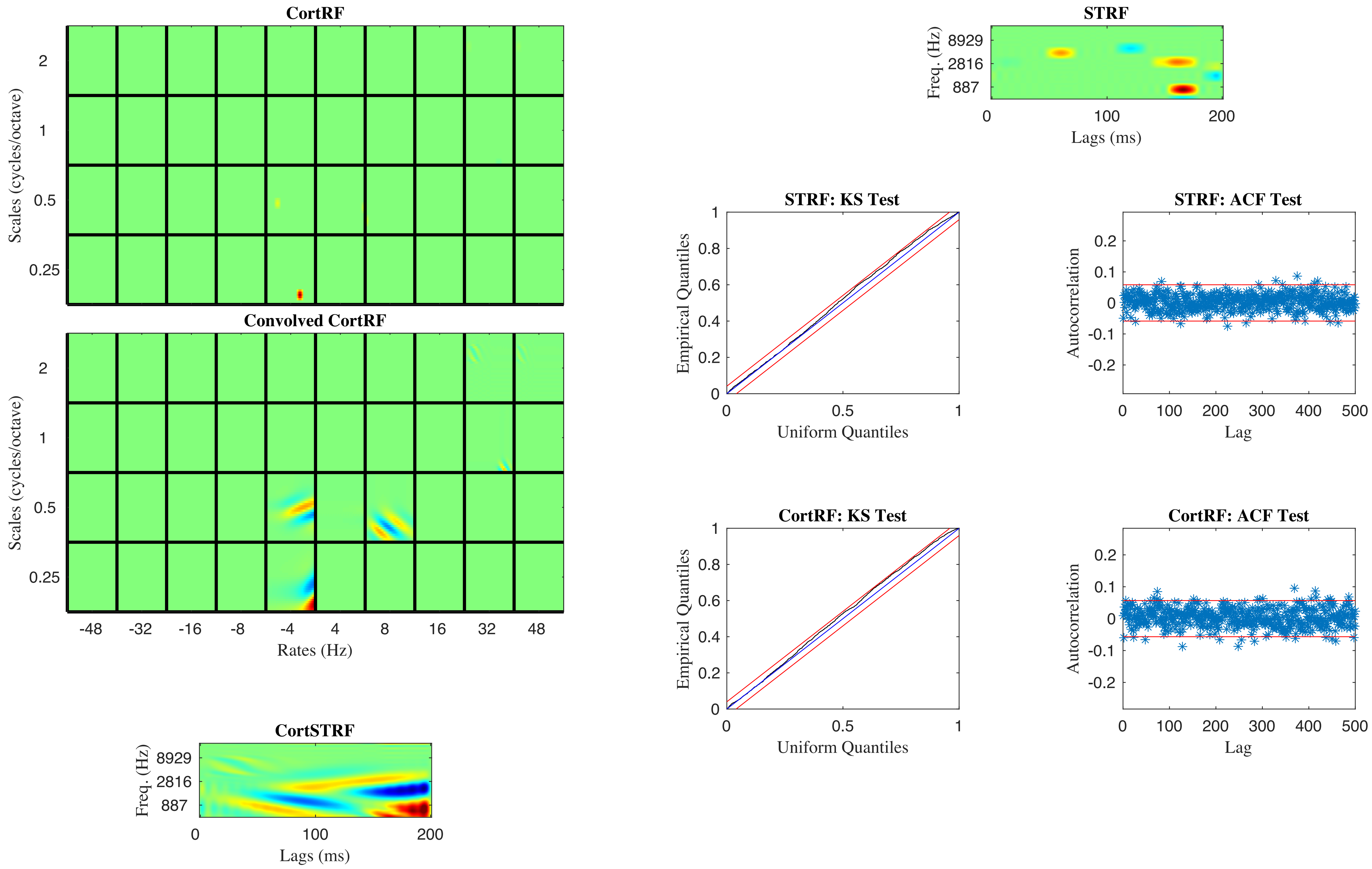
CortRF
Scales (cycles/octave)
2
1
0.5
0.25
Convolved CortRF
Scales (cycles/octave)
2
1
0.5
0.25
-48
-32
-16
-8
-4
4
8
16
32
48
Rates (Hz)
CortSTRF
Freq. (Hz)
8929
2816
887
0
100
200
Lags (ms)
STRF
Freq. (Hz)
8929
2816
887
0
100
200
Lags (ms)
STRF: KS Test
Empirical Quantiles
Uniform Quantiles
STRF: ACF Test
Autocorrelation
Lag
CortRF: KS Test
Empirical Quantiles
Uniform Quantiles
CortRF: ACF Test
Autocorrelation
Lag

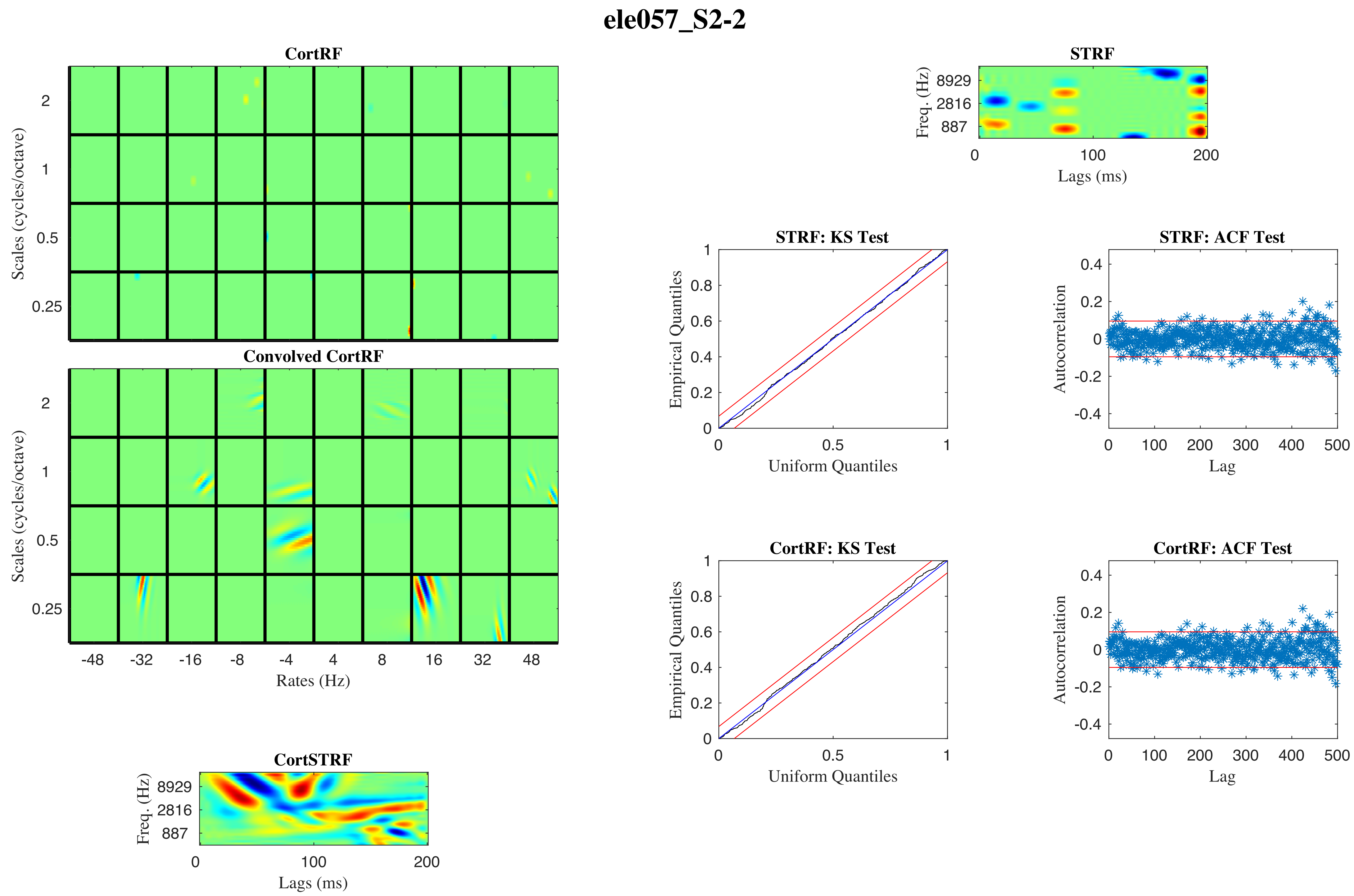

ele057_S2-2
CortRF
Scales (cycles/octave)
2
1
0.5
0.25
Convolved CortRF
Scales (cycles/octave)
2
1
0.5
0.25
-48 -32 -16 -8 -4 4 8 16 32 48
Rates (Hz)
CortSTRF
Freq. (Hz)
8929
2816
887
0 100 200
Lags (ms)
STRF
Freq. (Hz)
8929
2816
887
0 100 200
Lags (ms)
STRF: KS Test
Empirical Quantiles
Uniform Quantiles
STRF: ACF Test
Autocorrelation
Lag
CortRF: KS Test
Empirical Quantiles
Uniform Quantiles
CortRF: ACF Test
Autocorrelation
Lag

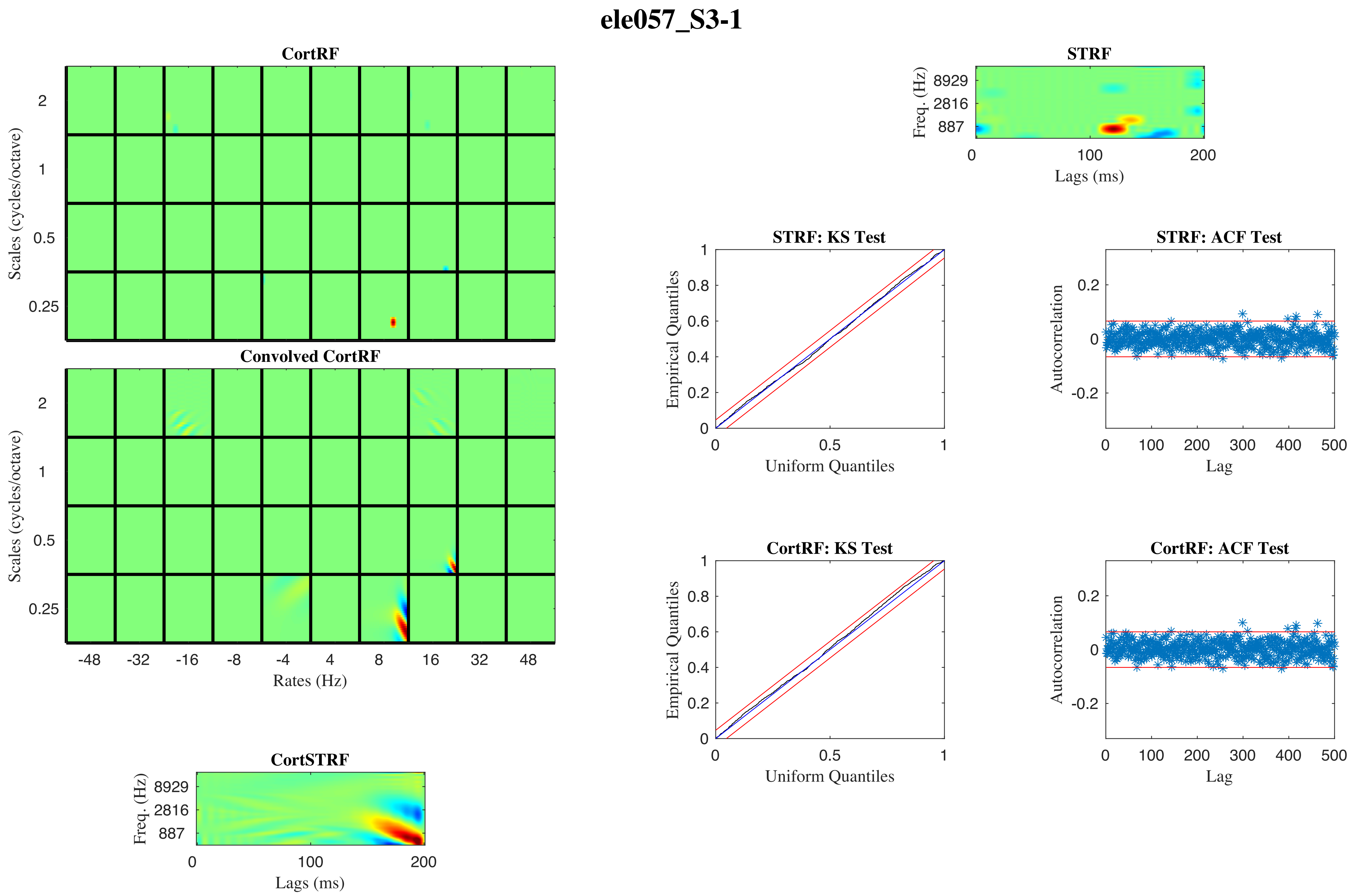
ele057_S3-1
CortRF
Scales (cycles/octave)
2
1
0.5
0.25
Convolved CortRF
-48
-32
-16
-8
-4
4
8
16
32
48
Rates (Hz)
CortSTRF
Freq. (Hz)
8929
2816
887
Lags (ms)
STRF
STRF: KS Test
Empirical Quantiles
Uniform Quantiles
STRF: ACF Test
Autocorrelation
Lag
CortRF: KS Test
CortRF: ACF Test

ele058_S1-2

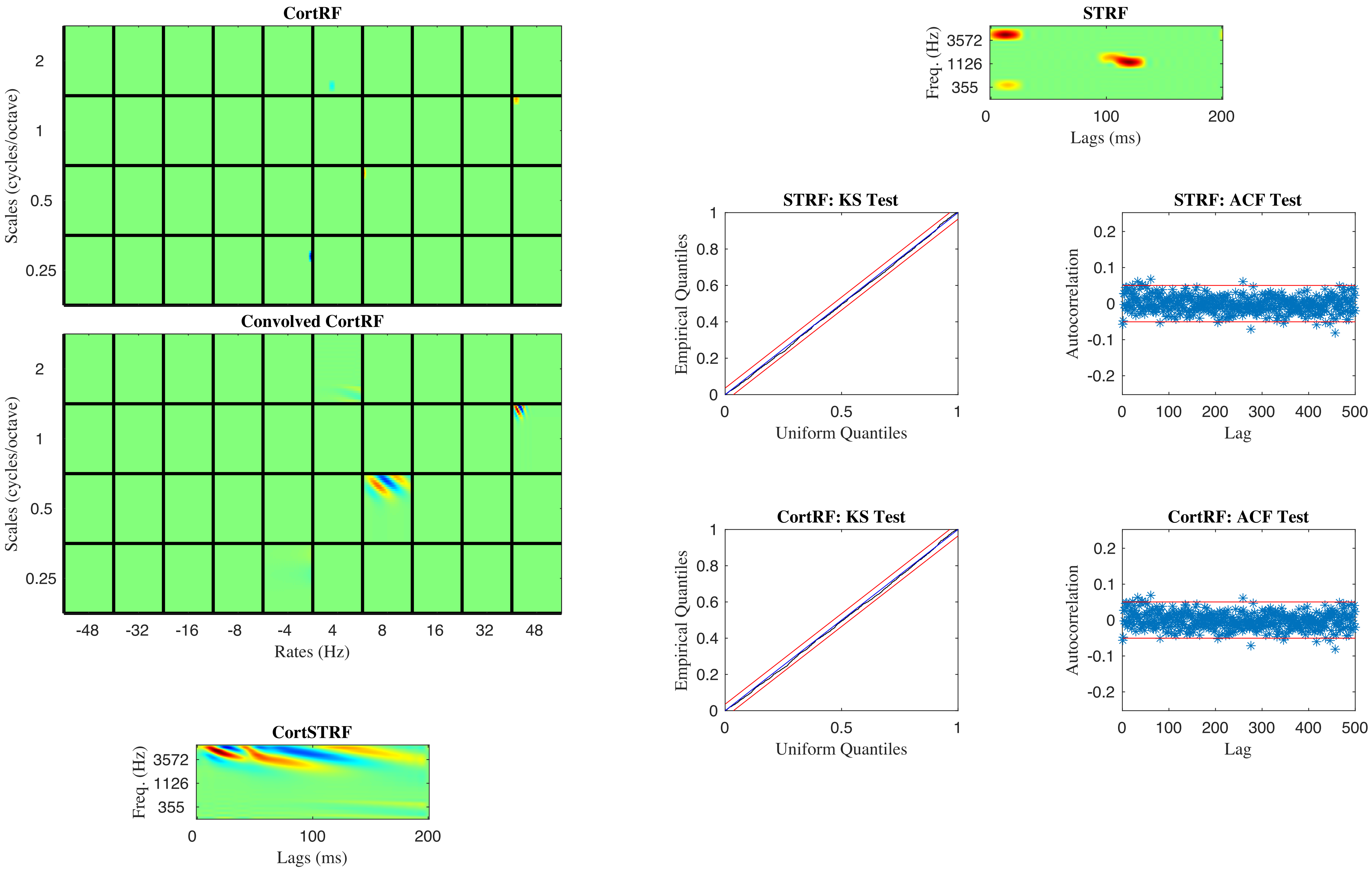
CortRF
Scales (cycles/octave)
2
1
0.5
0.25
Convolved CortRF
Rates (Hz)
-48
-32
-16
-8
-4
4
8
16
32
48
CortSTRF
Freq. (Hz)
3572
1126
355
Lags (ms)
0
100
200
STRF
STRF: KS Test
Empirical Quantiles
Uniform Quantiles
STRF: ACF Test
Autocorrelation
Lag
CortRF: KS Test
CortRF: ACF Test

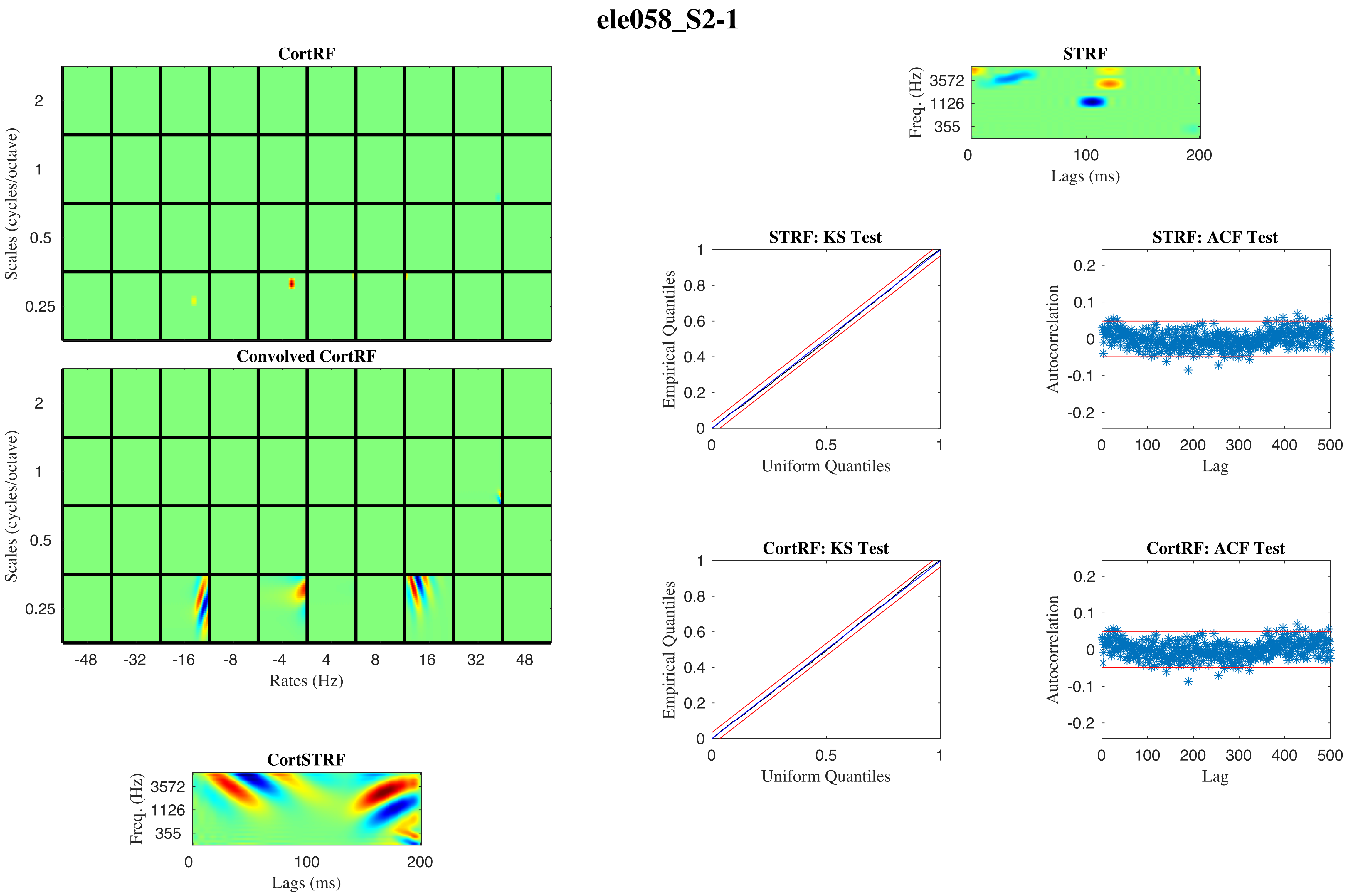
ele058_S2-1
CortRF
Scales (cycles/octave)
2
1
0.5
0.25
Convolved CortRF
Scales (cycles/octave)
2
1
0.5
0.25
-48 -32 -16 -8 -4 4 8 16 32 48
Rates (Hz)
CortSTRF
Freq. (Hz)
3572
1126
355
0 100 200
Lags (ms)
STRF
Freq. (Hz)
3572
1126
355
0 100 200
Lags (ms)
STRF: KS Test
Empirical Quantiles
Uniform Quantiles
STRF: ACF Test
Autocorrelation
Lag
CortRF: KS Test
Empirical Quantiles
Uniform Quantiles
CortRF: ACF Test
Autocorrelation
Lag

ele058_S2-2

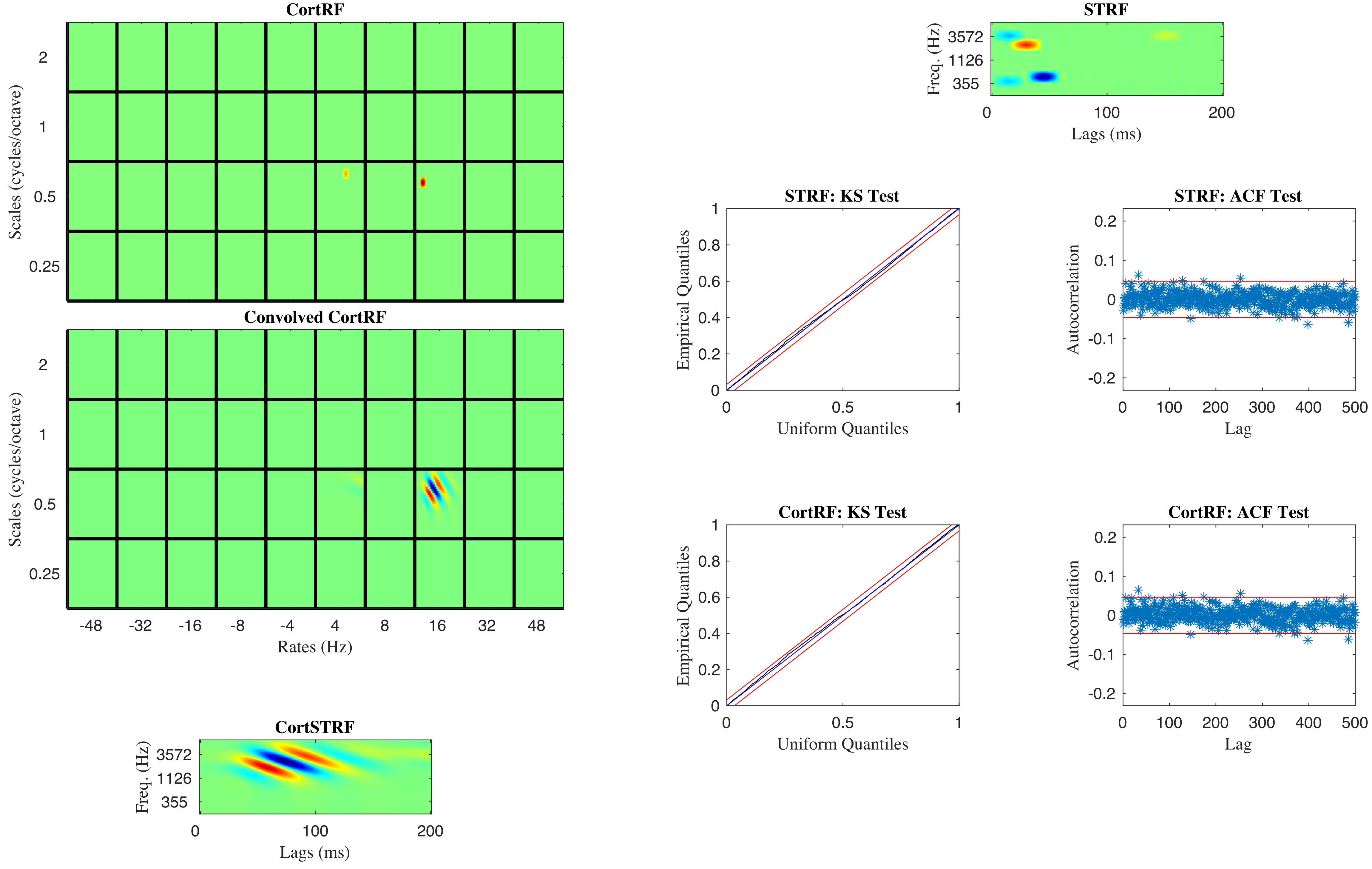
CortRF
Scales (cycles/octave)
2
1
0.5
0.25
Convolved CortRF
Scales (cycles/octave)
2
1
0.5
0.25
-48
-32
-16
-8
-4
4
8
16
32
48
Rates (Hz)
CortSTRF
Freq. (Hz)
3572
1126
355
0
100
200
Lags (ms)
STRF
Freq. (Hz)
3572
1126
355
0
100
200
Lags (ms)
STRF: KS Test
Empirical Quantiles
Uniform Quantiles
STRF: ACF Test
Autocorrelation
Lag
CortRF: KS Test
Empirical Quantiles
Uniform Quantiles
CortRF: ACF Test
Autocorrelation
Lag

ele058_S3-1

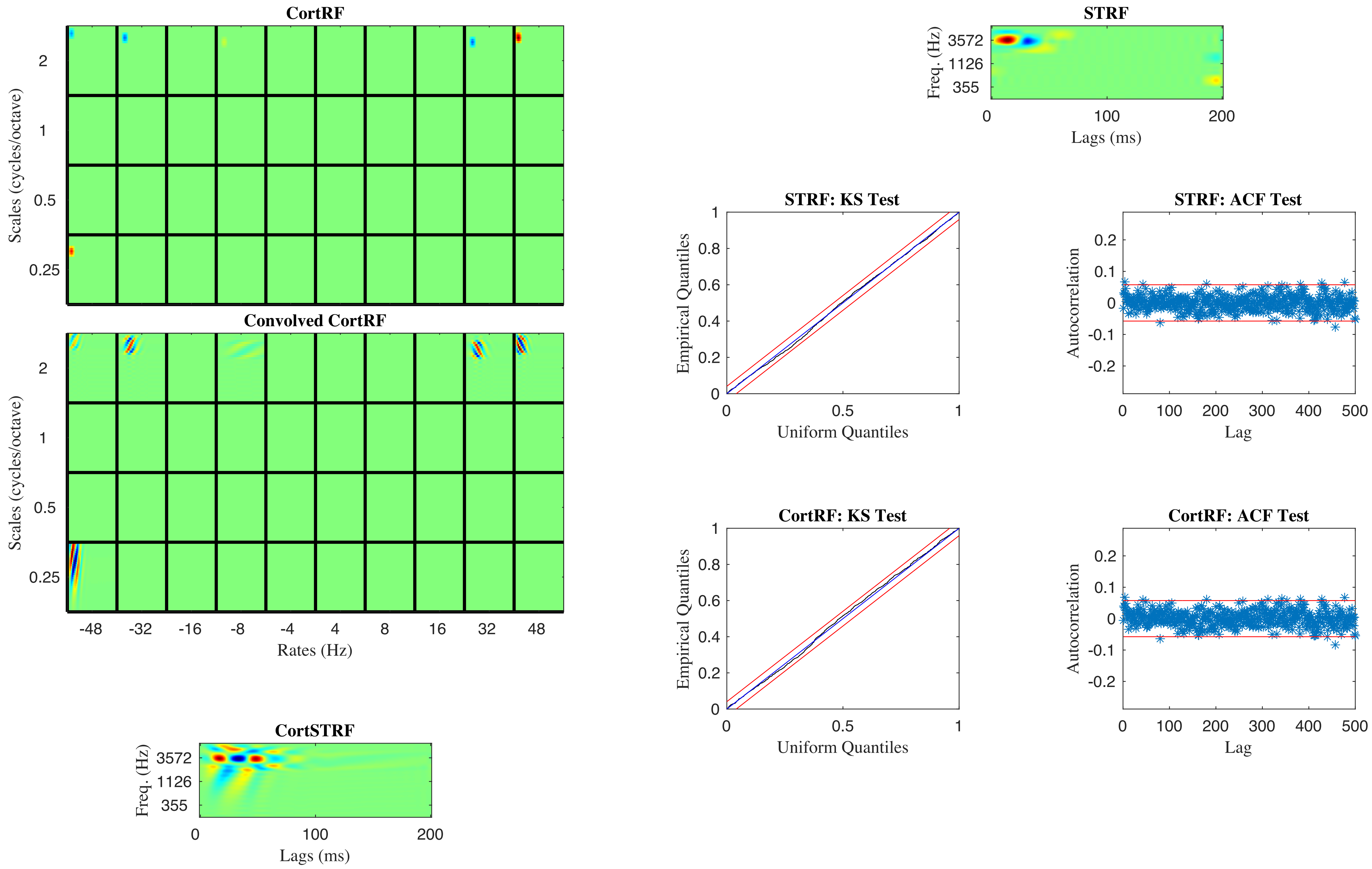
CortRF
Scales (cycles/octave)
2
1
0.5
0.25
Convolved CortRF
Scales (cycles/octave)
2
1
0.5
0.25
-48 -32 -16 -8 -4 4 8 16 32 48
Rates (Hz)
CortSTRF
Freq. (Hz)
3572
1126
355
0 100 200
Lags (ms)
STRF
Freq. (Hz)
3572
1126
355
0 100 200
Lags (ms)
STRF: KS Test
Empirical Quantiles
0 0.2 0.4 0.6 0.8 1
0 0.5 1
Uniform Quantiles
STRF: ACF Test
Autocorrelation
0.2 0.1 0 -0.1 -0.2
0 100 200 300 400 500
Lag
CortRF: KS Test
Empirical Quantiles
0 0.2 0.4 0.6 0.8 1
0 0.5 1
Uniform Quantiles
CortRF: ACF Test
Autocorrelation
0.2 0.1 0 -0.1 -0.2
0 100 200 300 400 500
Lag

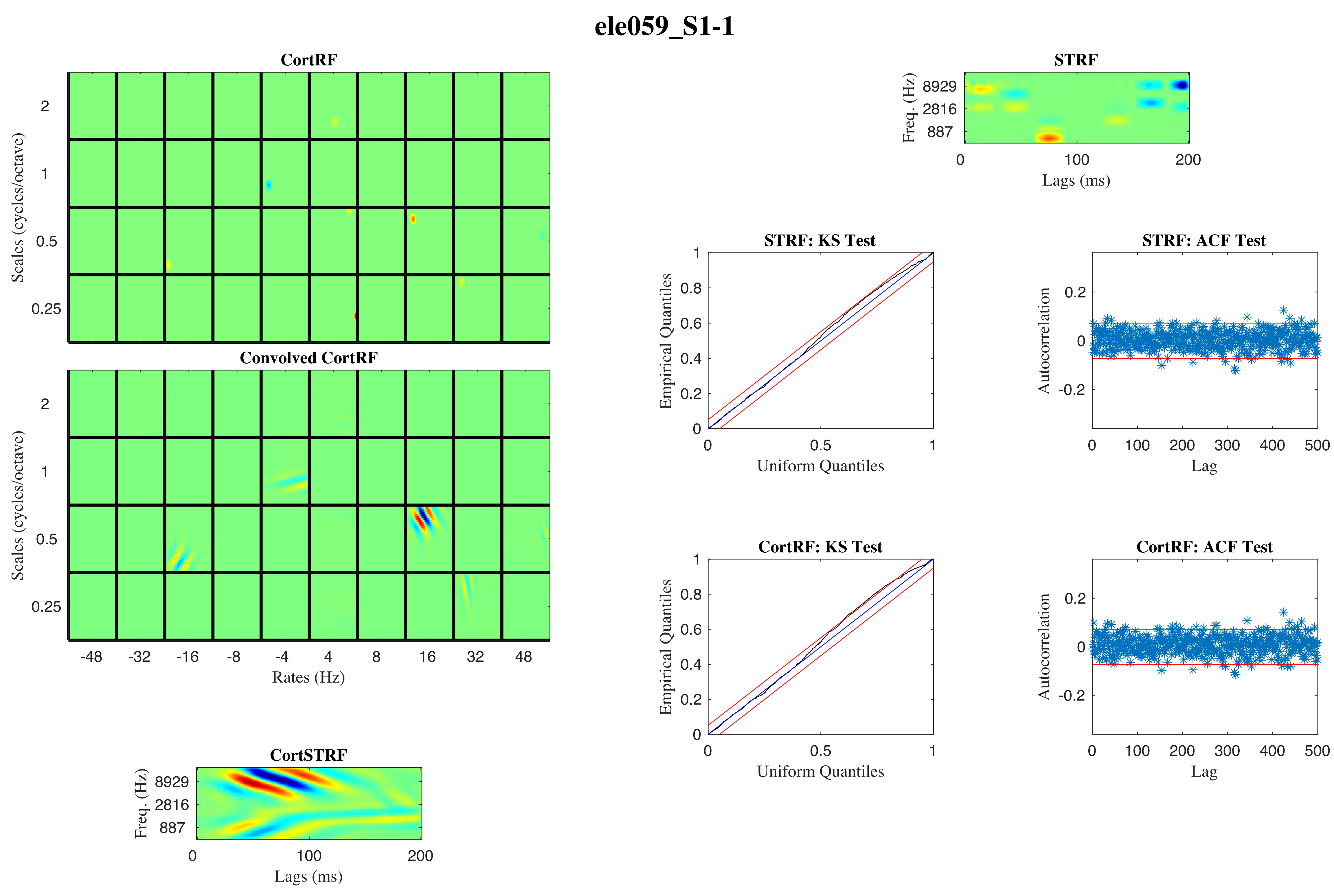
ele059_S1-1
CortRF
Scales (cycles/octave)
2
1
0.5
0.25
Convolved CortRF
-48
-32
-16
-8
-4
4
8
16
32
48
Rates (Hz)
CortSTRF
Freq. (Hz)
8929
2816
887
0
100
200
Lags (ms)
STRF
STRF: KS Test
Empirical Quantiles
Uniform Quantiles
STRF: ACF Test
Autocorrelation
Lag
CortRF: KS Test
CortRF: ACF Test
0
0.2
0.4
0.6
0.8
1
0.5
-0.2
300
400
500

ele059_S1-2

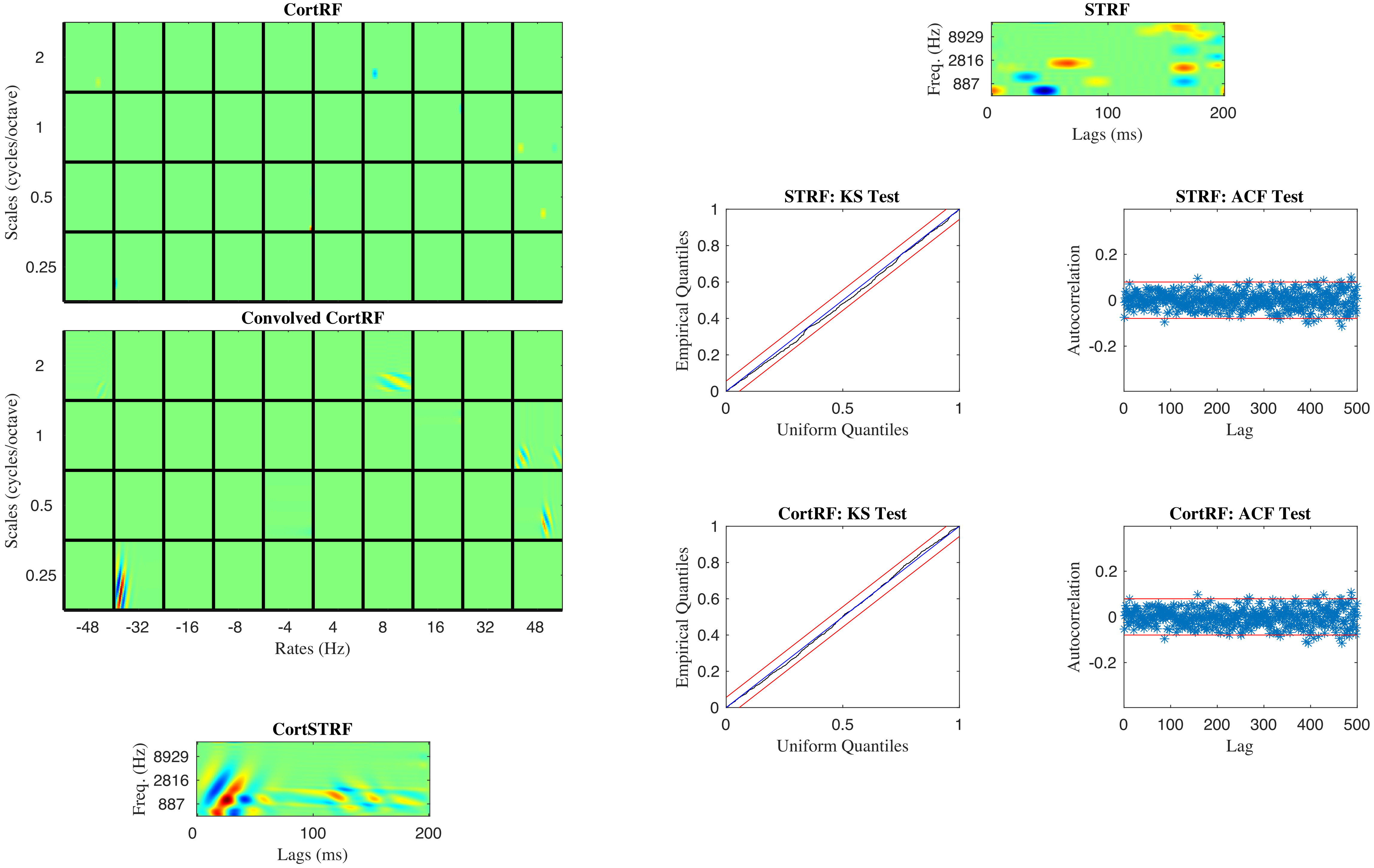

CortRF
Scales (cycles/octave)
2
1
0.5
0.25
Convolved CortRF
Scales (cycles/octave)
2
1
0.5
0.25
-48 -32 -16 -8 -4 4 8 16 32 48
Rates (Hz)
CortSTRF
Freq. (Hz)
8929
2816
887
0 100 200
Lags (ms)
STRF
Freq. (Hz)
8929
2816
887
0 100 200
Lags (ms)
STRF: KS Test
Empirical Quantiles
Uniform Quantiles
STRF: ACF Test
Autocorrelation
Lag
CortRF: KS Test
Empirical Quantiles
Uniform Quantiles
CortRF: ACF Test
Autocorrelation
Lag

ele059_S2-1

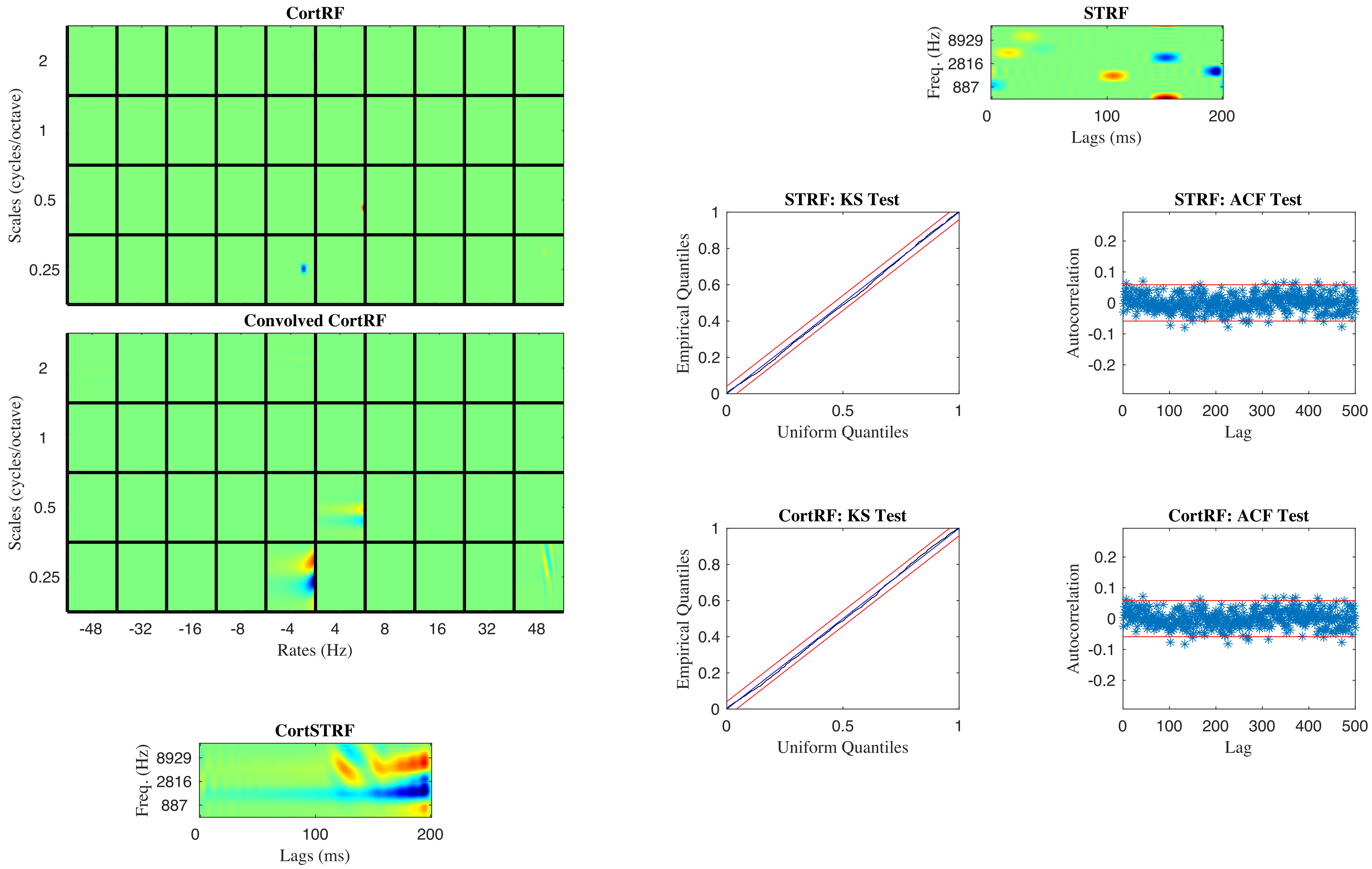

CortRF
Scales (cycles/octave)
2
1
0.5
0.25
Convolved CortRF
Scales (cycles/octave)
2
1
0.5
0.25
-48 -32 -16 -8 -4 4 8 16 32 48
Rates (Hz)
CortSTRF
Freq. (Hz)
8929
2816
887
0 100 200
Lags (ms)
STRF
Freq. (Hz)
8929
2816
887
0 100 200
Lags (ms)
STRF: KS Test
Empirical Quantiles
Uniform Quantiles
STRF: ACF Test
Autocorrelation
Lag
CortRF: KS Test
Empirical Quantiles
Uniform Quantiles
CortRF: ACF Test
Autocorrelation
Lag

ele059_S3-1

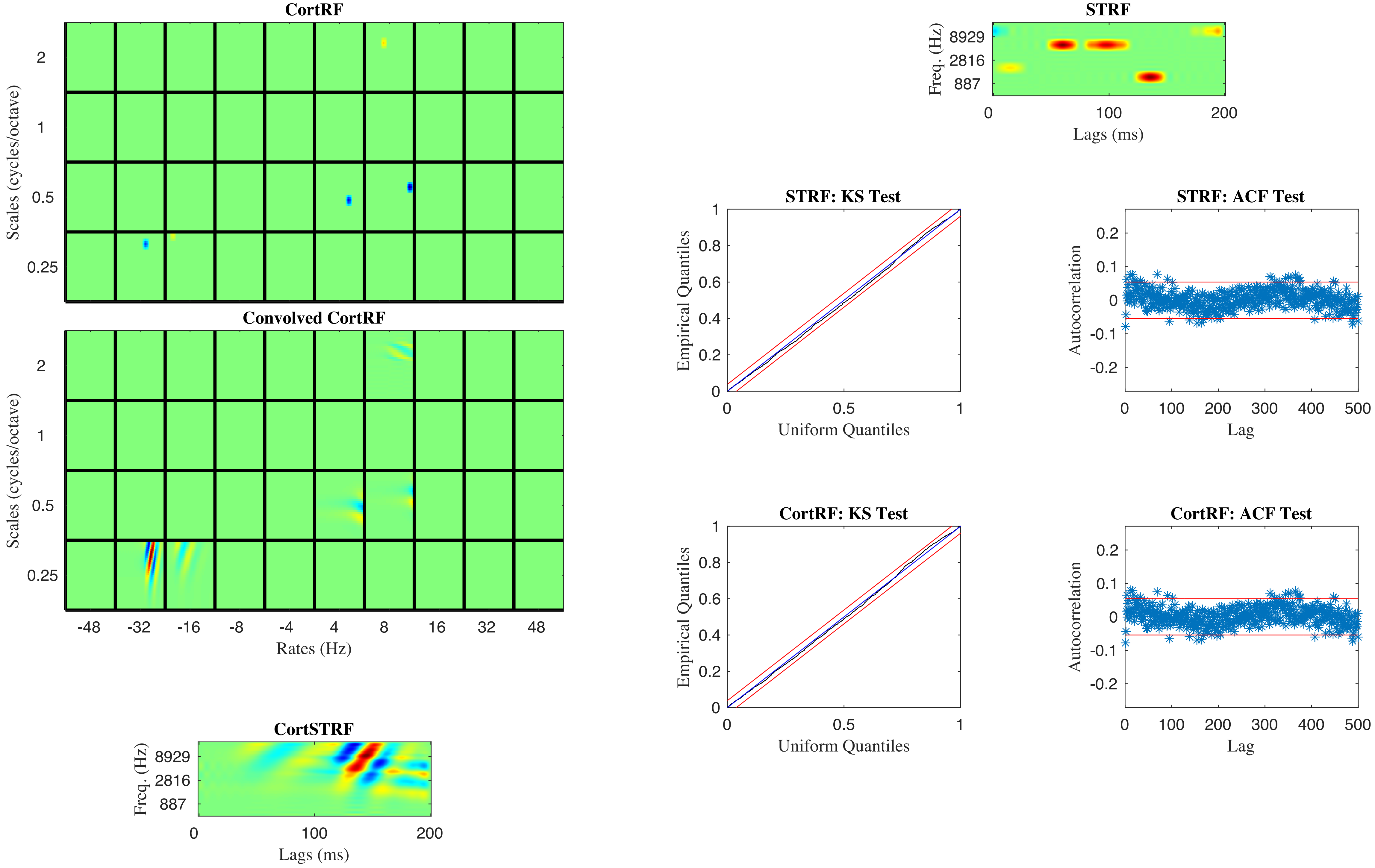
CortRF
Scales (cycles/octave)
2
1
0.5
0.25
Convolved CortRF
Scales (cycles/octave)
2
1
0.5
0.25
-48
-32
-16
-8
-4
4
8
16
32
48
Rates (Hz)
CortSTRF
Freq. (Hz)
8929
2816
887
0
100
200
Lags (ms)
STRF
Freq. (Hz)
8929
2816
887
0
100
200
Lags (ms)
STRF: KS Test
Empirical Quantiles
Uniform Quantiles
0
0.2
0.4
0.6
0.8
1
0.5
STRF: ACF Test
Autocorrelation
Lag
0.2
0.1
0
-0.1
-0.2
100
200
300
400
500
CortRF: KS Test
Empirical Quantiles
Uniform Quantiles
0
0.2
0.4
0.6
0.8
1
0.5
CortRF: ACF Test
Autocorrelation
Lag
0.2
0.1
0
-0.1
-0.2
100
200
300
400
500

ele060_S2-1

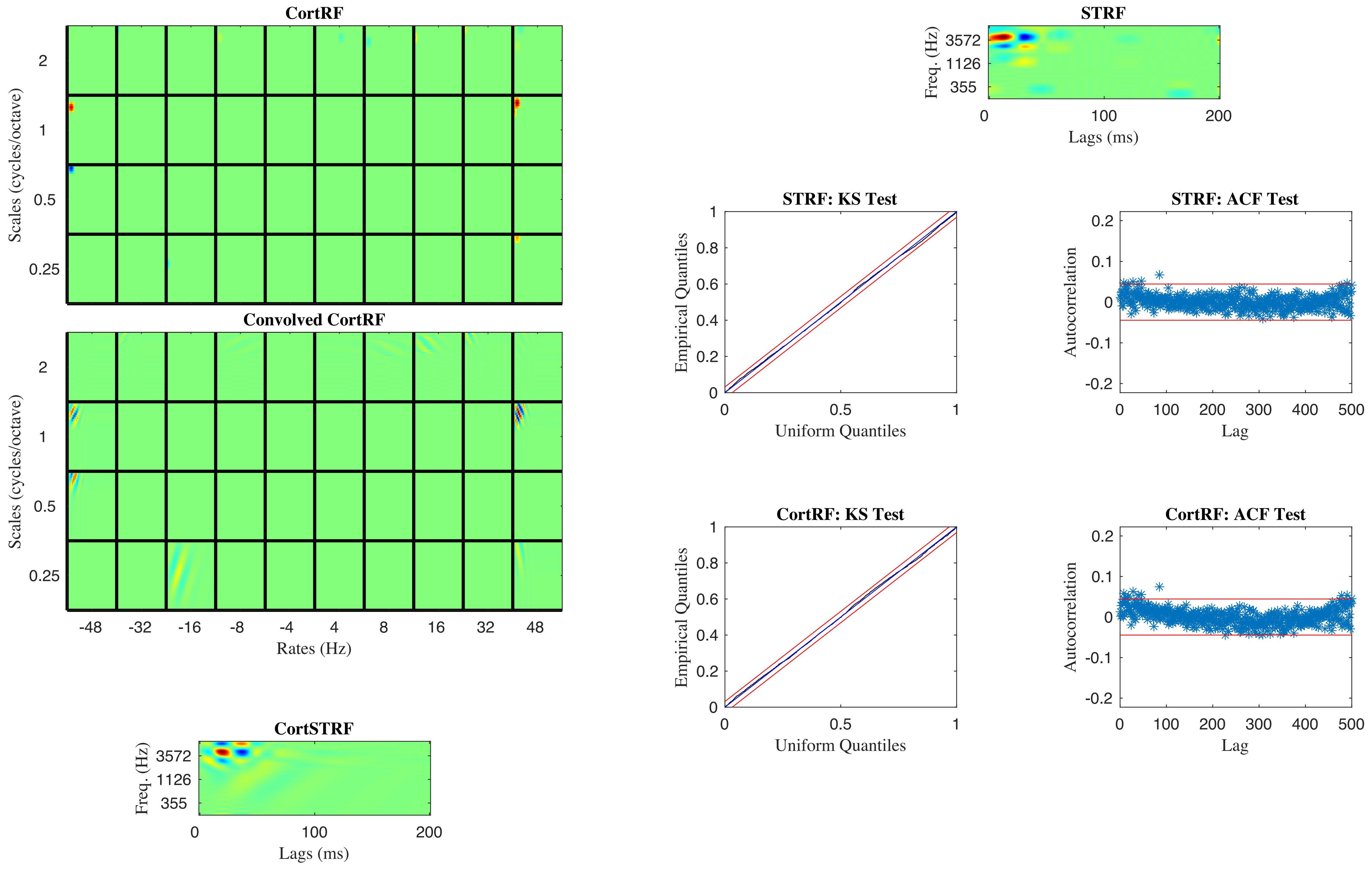
CortRF
Scales (cycles/octave)
2
1
0.5
0.25
Convolved CortRF
Scales (cycles/octave)
2
1
0.5
0.25
-48 -32 -16 -8 -4 4 8 16 32 48
Rates (Hz)
CortSTRF
Freq. (Hz)
3572
1126
355
0 100 200
Lags (ms)
STRF
Freq. (Hz)
3572
1126
355
0 100 200
Lags (ms)
STRF: KS Test
Empirical Quantiles
0 0.2 0.4 0.6 0.8 1
0 0.5 1
Uniform Quantiles
STRF: ACF Test
Autocorrelation
-0.2 -0.1 0 0.1 0.2
0 100 200 300 400 500
Lag
CortRF: KS Test
Empirical Quantiles
0 0.2 0.4 0.6 0.8 1
0 0.5 1
Uniform Quantiles
CortRF: ACF Test
Autocorrelation
-0.2 -0.1 0 0.1 0.2
0 100 200 300 400 500
Lag

ele060_S2-2

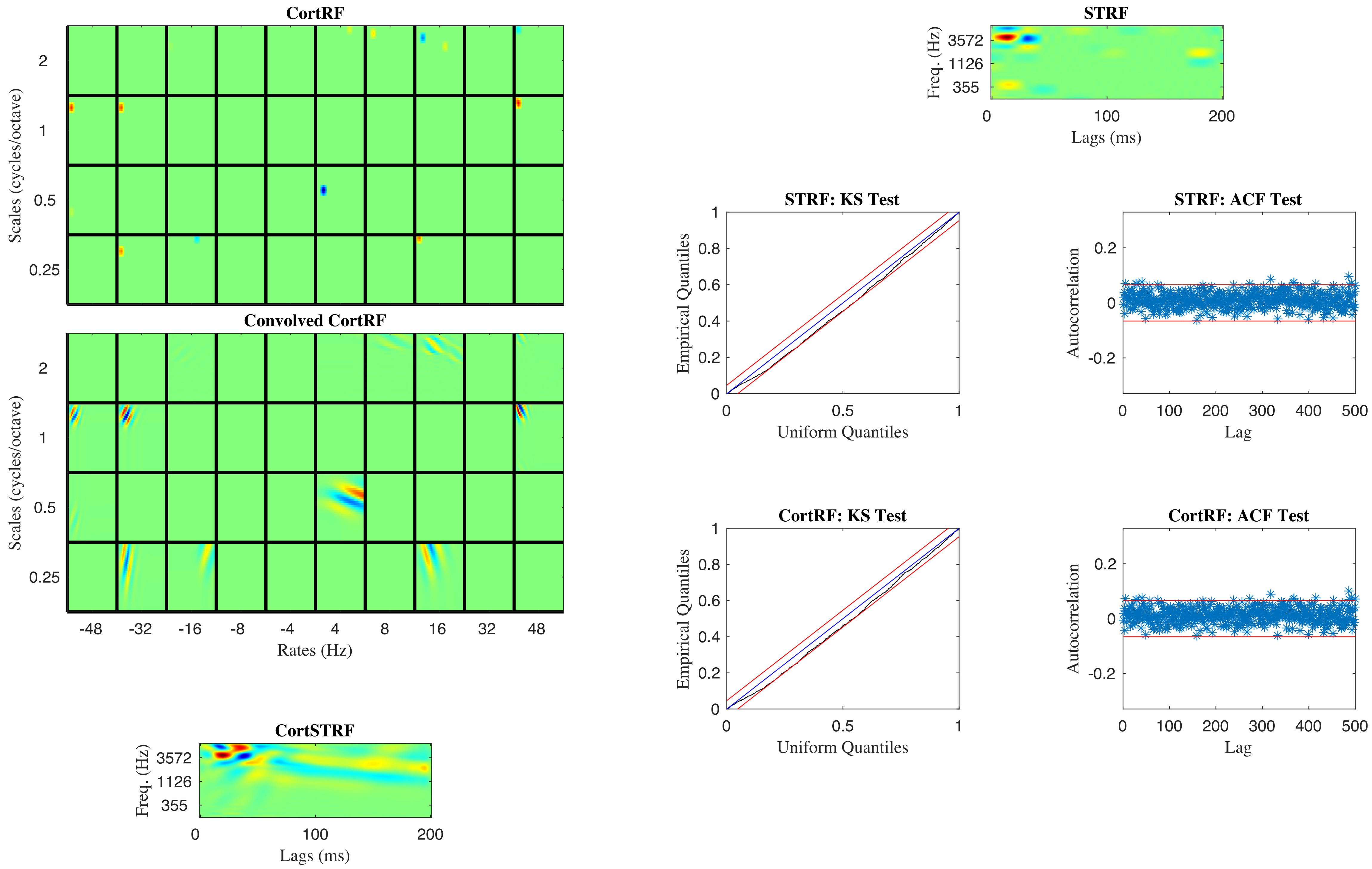

CortRF
Scales (cycles/octave)
2
1
0.5
0.25
Convolved CortRF
-48
-32
-16
-8
-4
4
8
16
32
48
Rates (Hz)
CortSTRF
Freq. (Hz)
3572
1126
355
0
100
200
Lags (ms)
STRF
STRF: KS Test
Empirical Quantiles
Uniform Quantiles
STRF: ACF Test
Autocorrelation
Lag
0.2
0
-0.2
0
100
200
300
400
500
CortRF: KS Test
CortRF: ACF Test
0
0.2
0.4
0.6
0.8
1
0.5

ele061_S2-1

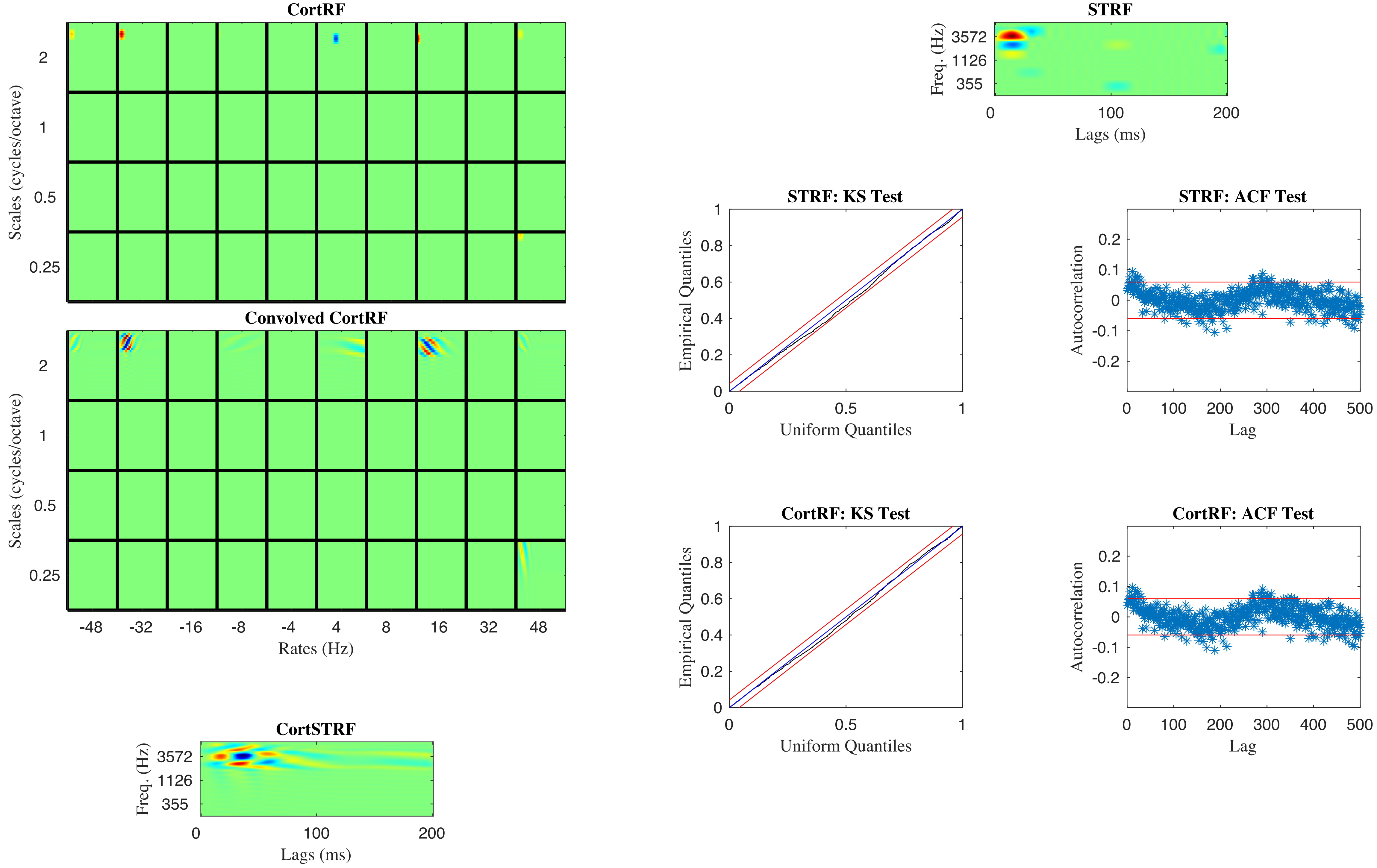
CortRF
Scales (cycles/octave)
2
1
0.5
0.25
Convolved CortRF
Scales (cycles/octave)
2
1
0.5
0.25
-48 -32 -16 -8 -4 4 8 16 32 48
Rates (Hz)
CortSTRF
Freq. (Hz)
3572
1126
355
0 100 200
Lags (ms)
STRF
Freq. (Hz)
3572
1126
355
0 100 200
Lags (ms)
STRF: KS Test
Empirical Quantiles
0 0.2 0.4 0.6 0.8 1
0 0.5 1
Uniform Quantiles
STRF: ACF Test
Autocorrelation
0.2 0.1 0 -0.1 -0.2
0 100 200 300 400 500
Lag
CortRF: KS Test
Empirical Quantiles
0 0.2 0.4 0.6 0.8 1
0 0.5 1
Uniform Quantiles
CortRF: ACF Test
Autocorrelation
0.2 0.1 0 -0.1 -0.2
0 100 200 300 400 500
Lag

ele061_S3-1

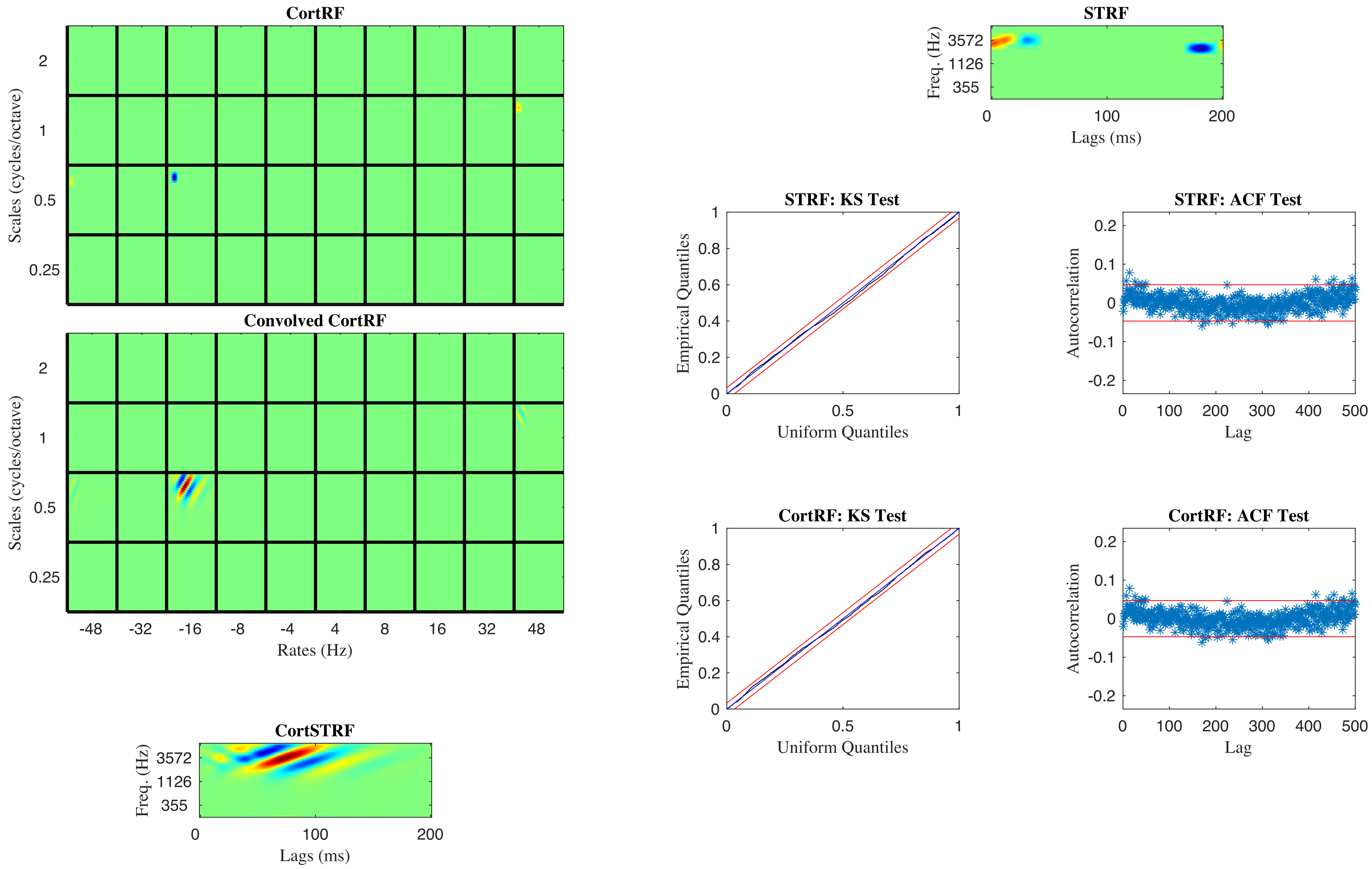
CortRF
Convolved CortRF
Scales (cycles/octave)
2
1
0.5
0.25
-48
-32
-16
-8
-4
4
8
16
32
48
Rates (Hz)
CortSTRF
STRF
Freq. (Hz)
3572
1126
355
0
100
200
Lags (ms)
STRF: KS Test
STRF: ACF Test
CortRF: KS Test
CortRF: ACF Test
Empirical Quantiles
Uniform Quantiles
Autocorrelation
Lag
0.2
0.1
0
-0.1
-0.2
1
0.8
0.6
0.4
0.5
300
400
500

ele061_S3-2

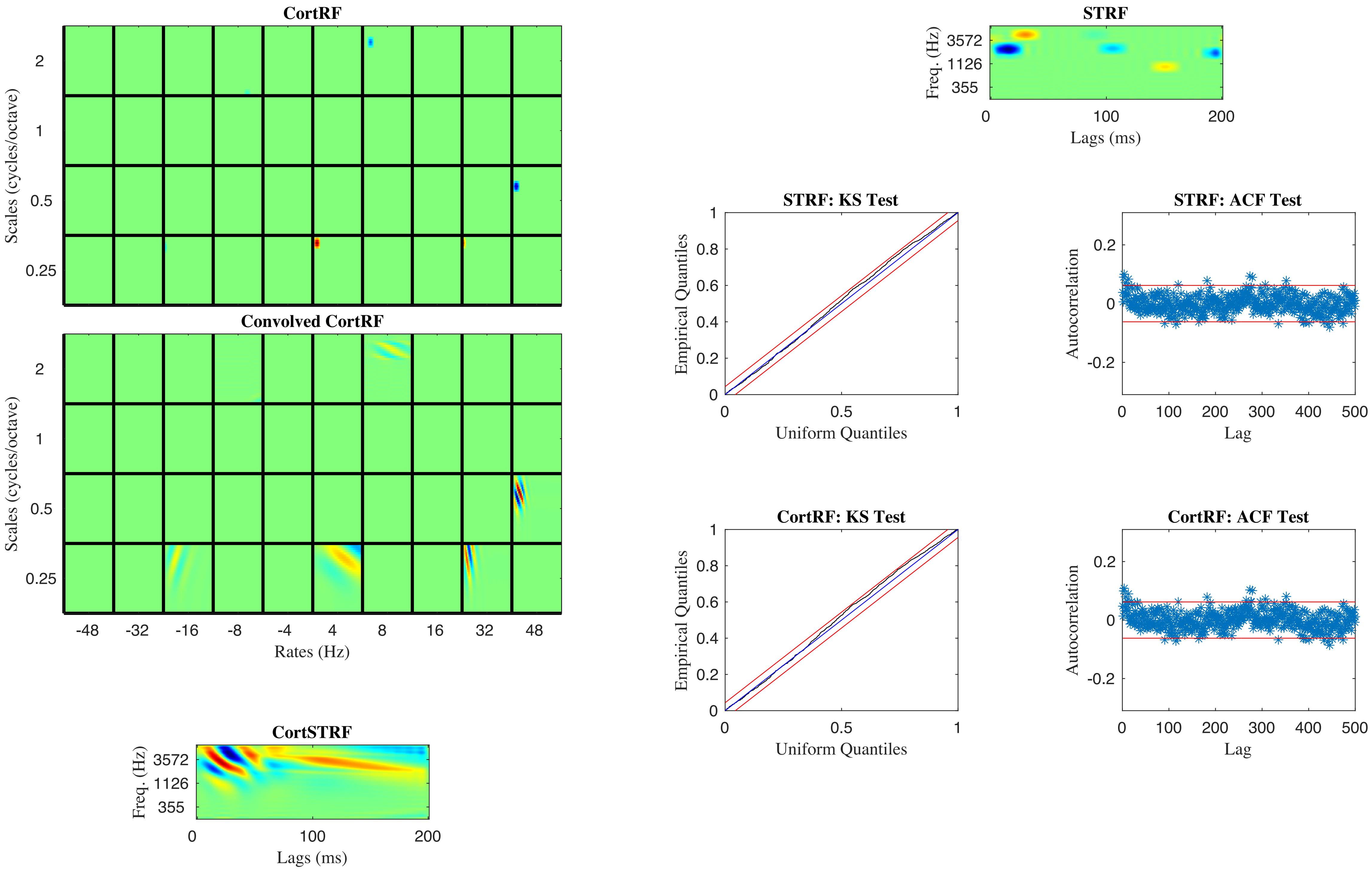

CortRF
Scales (cycles/octave)
2
1
0.5
0.25
Convolved CortRF
Scales (cycles/octave)
2
1
0.5
0.25
-48
-32
-16
-8
-4
4
8
16
32
48
Rates (Hz)
CortSTRF
Freq. (Hz)
3572
1126
355
0
100
200
Lags (ms)
STRF
Freq. (Hz)
3572
1126
355
0
100
200
Lags (ms)
STRF: KS Test
Empirical Quantiles
1
0.8
0.6
0.4
0.2
0
0
0.5
1
Uniform Quantiles
STRF: ACF Test
Autocorrelation
0.2
0
-0.2
0
100
200
300
400
500
Lag
CortRF: KS Test
Empirical Quantiles
1
0.8
0.6
0.4
0.2
0
0
0.5
1
Uniform Quantiles
CortRF: ACF Test
Autocorrelation
0.2
0
-0.2
0
100
200
300
400
500
Lag

ele061_S3-3

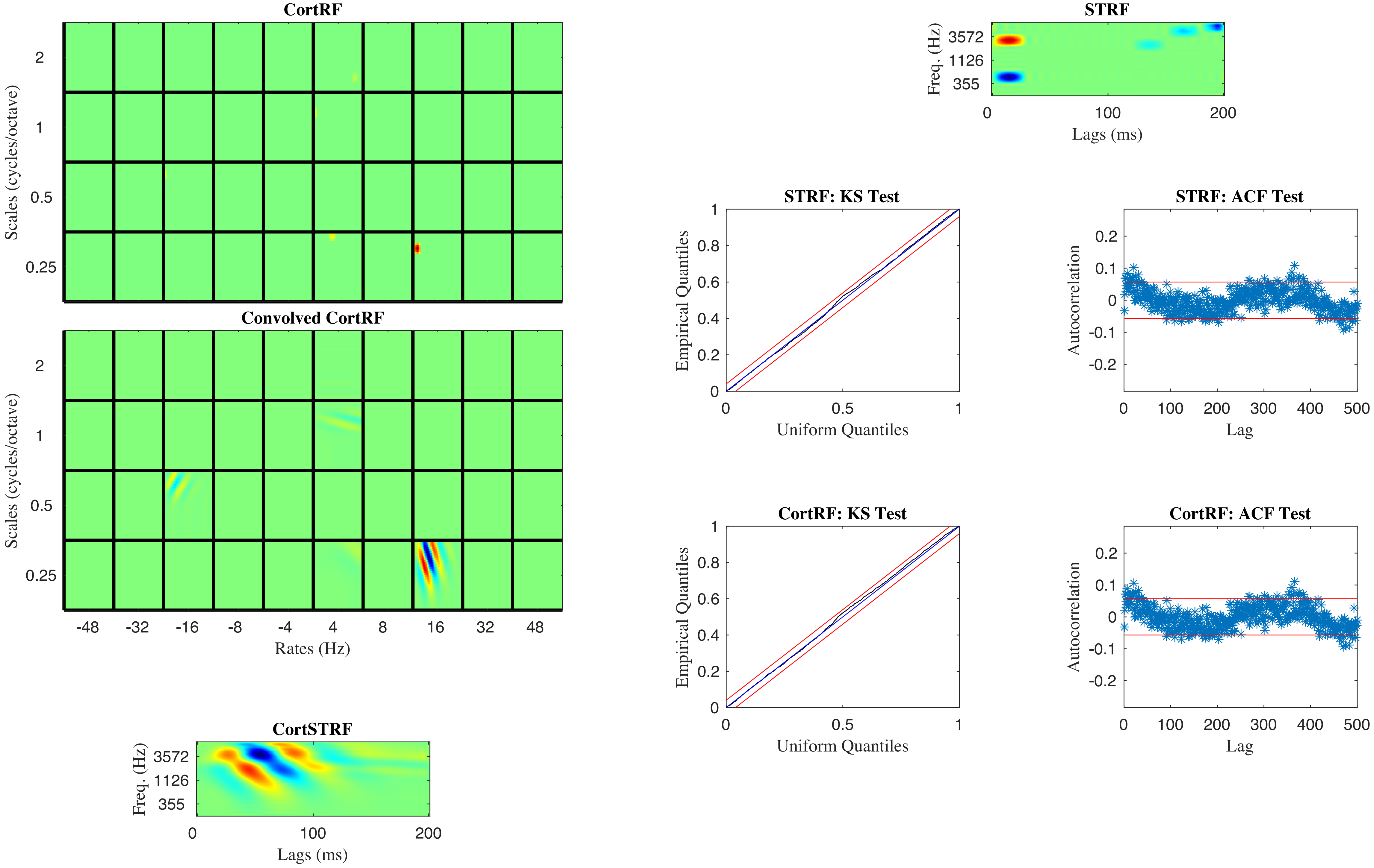
CortRF
Scales (cycles/octave)
2
1
0.5
0.25
Convolved CortRF
Scales (cycles/octave)
2
1
0.5
0.25
-48
-32
-16
-8
-4
4
8
16
32
48
Rates (Hz)
CortSTRF
Freq. (Hz)
3572
1126
355
0
100
200
Lags (ms)
STRF
Freq. (Hz)
3572
1126
355
0
100
200
Lags (ms)
STRF: KS Test
Empirical Quantiles
1
0.8
0.6
0.4
0.2
0
0
0.5
1
Uniform Quantiles
STRF: ACF Test
Autocorrelation
0.2
0.1
0
-0.1
-0.2
0
100
200
300
400
500
Lag
CortRF: KS Test
Empirical Quantiles
1
0.8
0.6
0.4
0.2
0
0
0.5
1
Uniform Quantiles
CortRF: ACF Test
Autocorrelation
0.2
0.1
0
-0.1
-0.2
0
100
200
300
400
500
Lag

ele062_S1-1

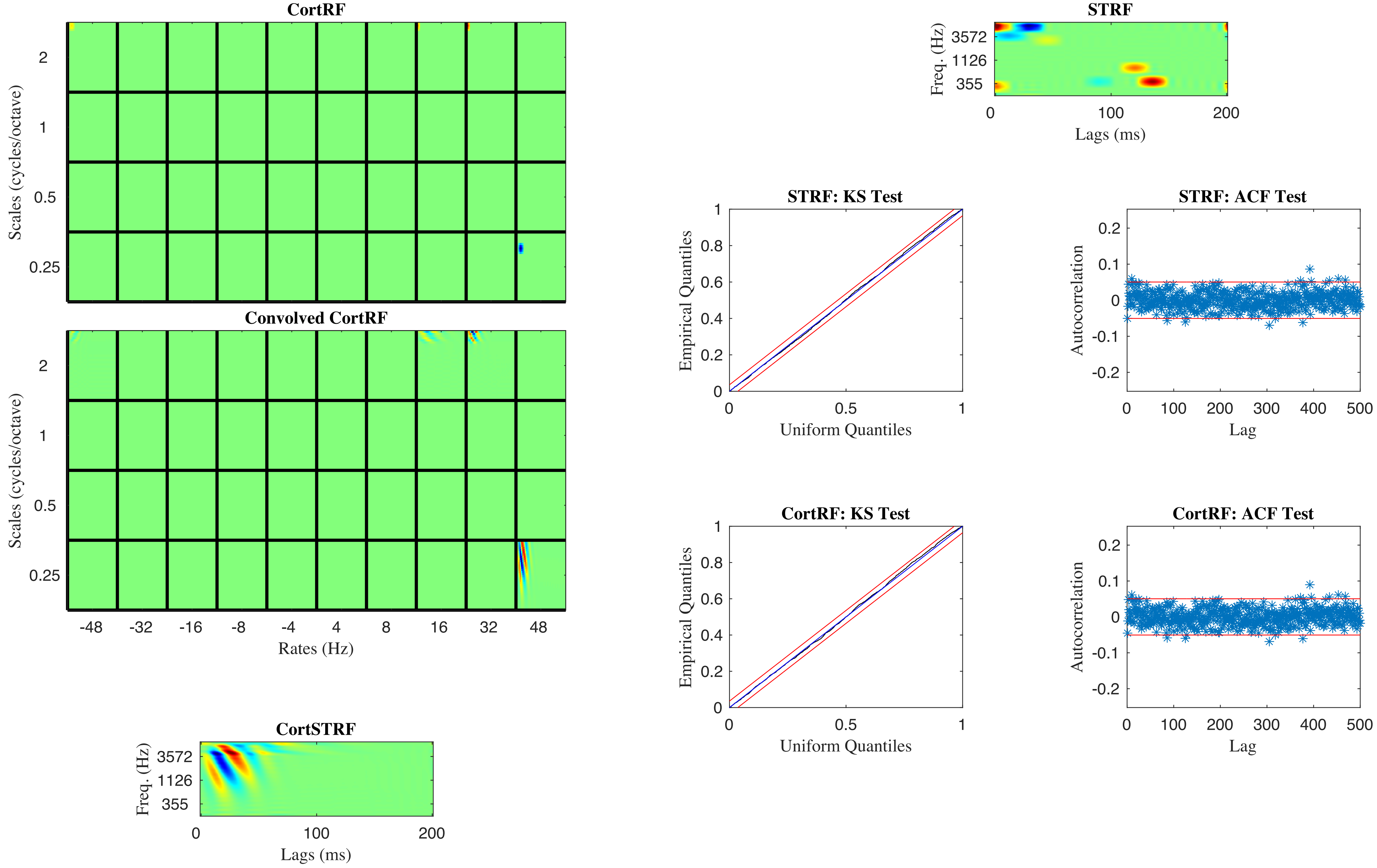

CortRF
Scales (cycles/octave)
2
1
0.5
0.25
Convolved CortRF
Scales (cycles/octave)
2
1
0.5
0.25
-48
-32
-16
-8
-4
4
8
16
32
48
Rates (Hz)
CortSTRF
Freq. (Hz)
3572
1126
355
0
100
200
Lags (ms)
STRF
Freq. (Hz)
3572
1126
355
0
100
200
Lags (ms)
STRF: KS Test
Empirical Quantiles
Uniform Quantiles
STRF: ACF Test
Autocorrelation
Lag
CortRF: KS Test
Empirical Quantiles
Uniform Quantiles
CortRF: ACF Test
Autocorrelation
Lag

ele062_S2-1

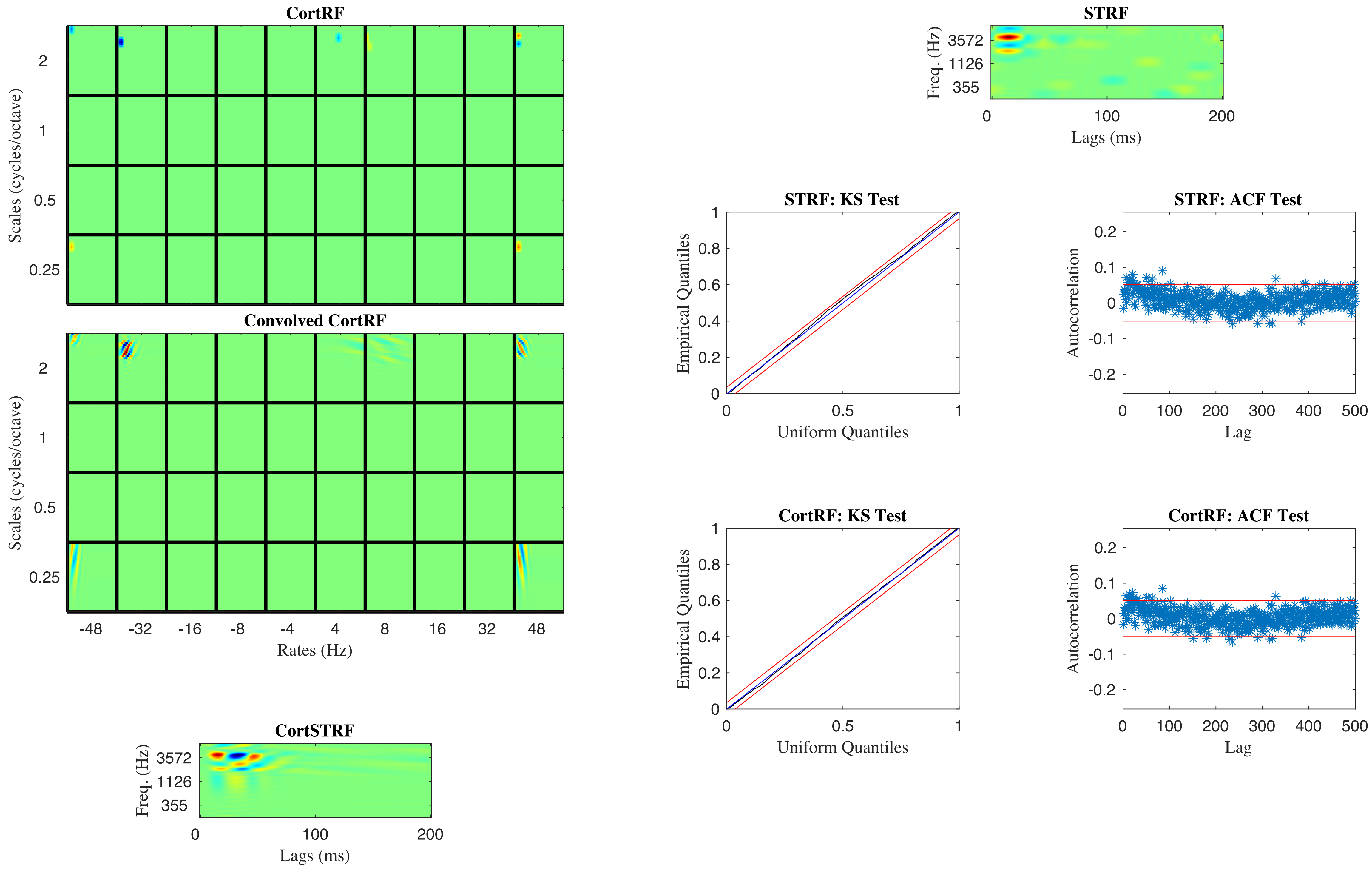
CortRF
Scales (cycles/octave)
2
1
0.5
0.25
Convolved CortRF
Scales (cycles/octave)
2
1
0.5
0.25
-48
-32
-16
-8
-4
4
8
16
32
48
Rates (Hz)
CortSTRF
Freq. (Hz)
3572
1126
355
0
100
200
Lags (ms)
STRF
Freq. (Hz)
3572
1126
355
0
100
200
Lags (ms)
STRF: KS Test
Empirical Quantiles
1
0.8
0.6
0.4
0.2
0
0
0.5
1
Uniform Quantiles
STRF: ACF Test
Autocorrelation
0.2
0.1
0
-0.1
-0.2
0
100
200
300
400
500
Lag
CortRF: KS Test
Empirical Quantiles
1
0.8
0.6
0.4
0.2
0
0
0.5
1
Uniform Quantiles
CortRF: ACF Test
Autocorrelation
0.2
0.1
0
-0.1
-0.2
0
100
200
300
400
500
Lag

ele062_S2-2

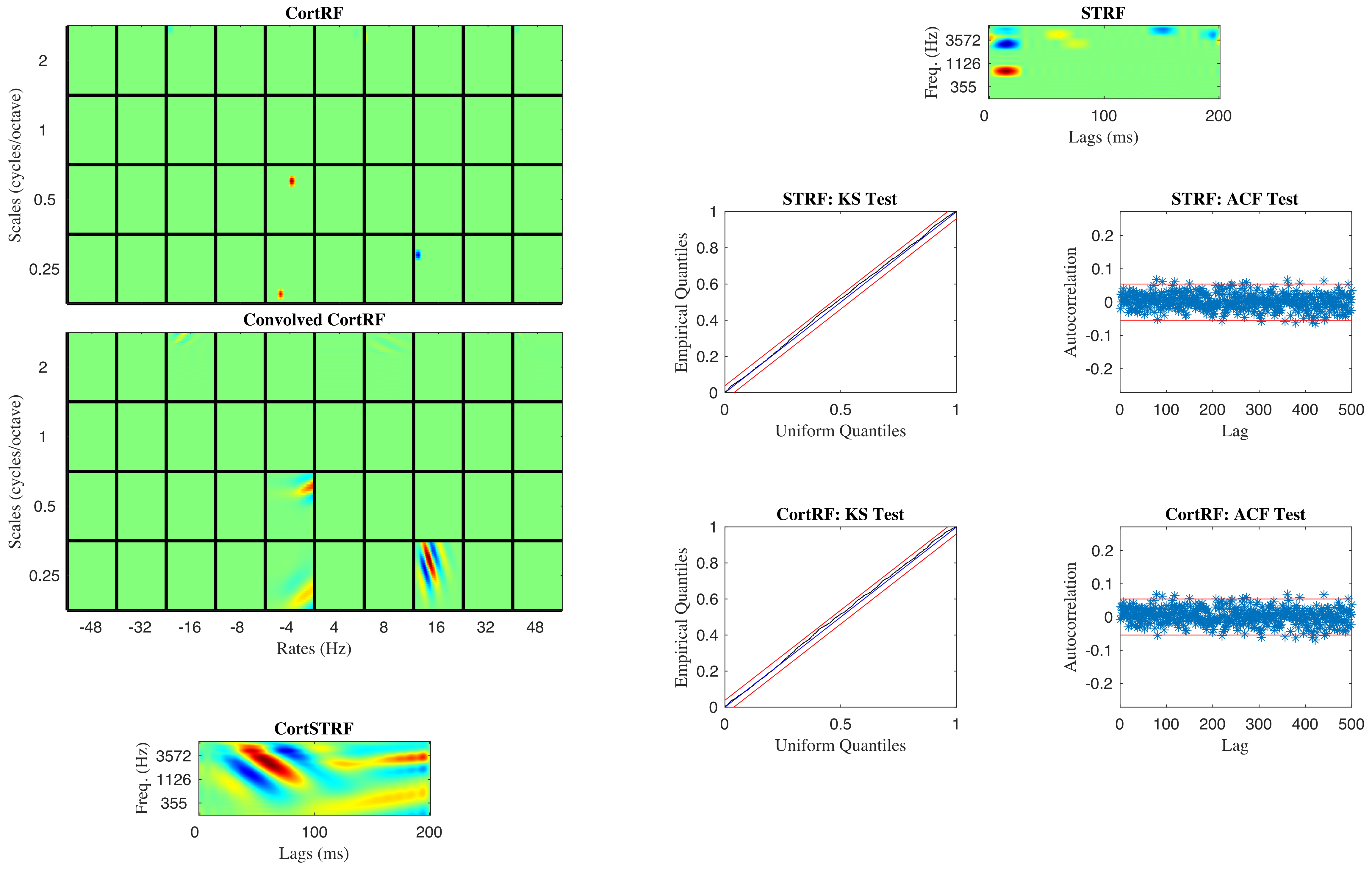

CortRF
Scales (cycles/octave)
2
1
0.5
0.25
Convolved CortRF
Scales (cycles/octave)
2
1
0.5
0.25
-48
-32
-16
-8
-4
4
8
16
32
48
Rates (Hz)
CortSTRF
Freq. (Hz)
3572
1126
355
0
100
200
Lags (ms)
STRF
Freq. (Hz)
3572
1126
355
0
100
200
Lags (ms)
STRF: KS Test
Empirical Quantiles
Uniform Quantiles
STRF: ACF Test
Autocorrelation
Lag
CortRF: KS Test
Empirical Quantiles
Uniform Quantiles
CortRF: ACF Test
Autocorrelation
Lag

ele062_S3-1

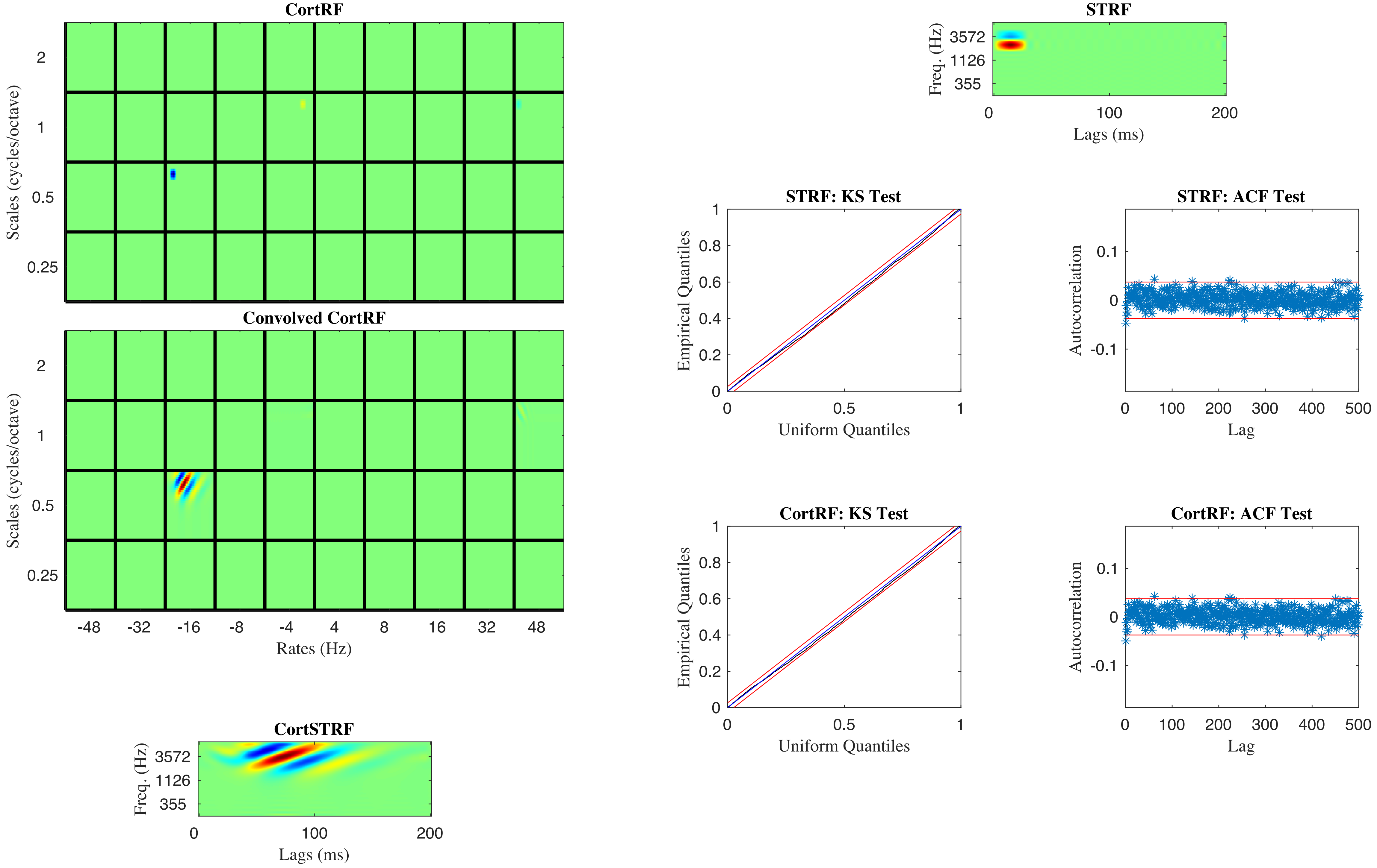
CortRF
Scales (cycles/octave)
2
1
0.5
0.25
Convolved CortRF
Scales (cycles/octave)
2
1
0.5
0.25
-48
-32
-16
-8
-4
4
8
16
32
48
Rates (Hz)
CortSTRF
Freq. (Hz)
3572
1126
355
0
100
200
Lags (ms)
STRF
Freq. (Hz)
3572
1126
355
0
100
200
Lags (ms)
STRF: KS Test
Empirical Quantiles
Uniform Quantiles
STRF: ACF Test
Autocorrelation
Lag
CortRF: KS Test
Empirical Quantiles
Uniform Quantiles
CortRF: ACF Test
Autocorrelation
Lag
