# Supplementary material for: Sparse high-dimensional decomposition of non-primary auditory cortical receptive fields
Source: PLoS Comput Biol. 2025 Jan 2;21(1):e1012721. doi: 10.1371/journal.pcbi.1012721 (PMC11774495; doi:10.1371/journal.pcbi.1012721)
Supplement: S3 Fig — This figures show all 6 CortRF clusters. (PDF) [file pcbi.1012721.s005.pdf]

Sparse high-dimensional decomposition of non-primary auditory cortical receptive fields

S3 Figure: CortRF cluster analysis

Shoutik Mukherjee[1,2], Behtash Babadi[1,2], Shihab Shamma[1,2,3] *

1 Department of Electrical and Computer Engineering, University of Maryland, College Park, Maryland, United States of America
2 Institute for Systems Research, University of Maryland, College Park, Maryland, United States of America
3 Laboratoire des Systèmes Perceptifs, Department des Études Cognitive, École Normale Supériure, Paris Sciences et Lettres University, Paris, France

*sas@umd.edu

Overview

This supporting information file contains all cluster average CortRFs (Fig S3). Three of the six clusters were small and hence were neither shown nor discussed in Fig. 8 of the main results; they are included here for completeness.

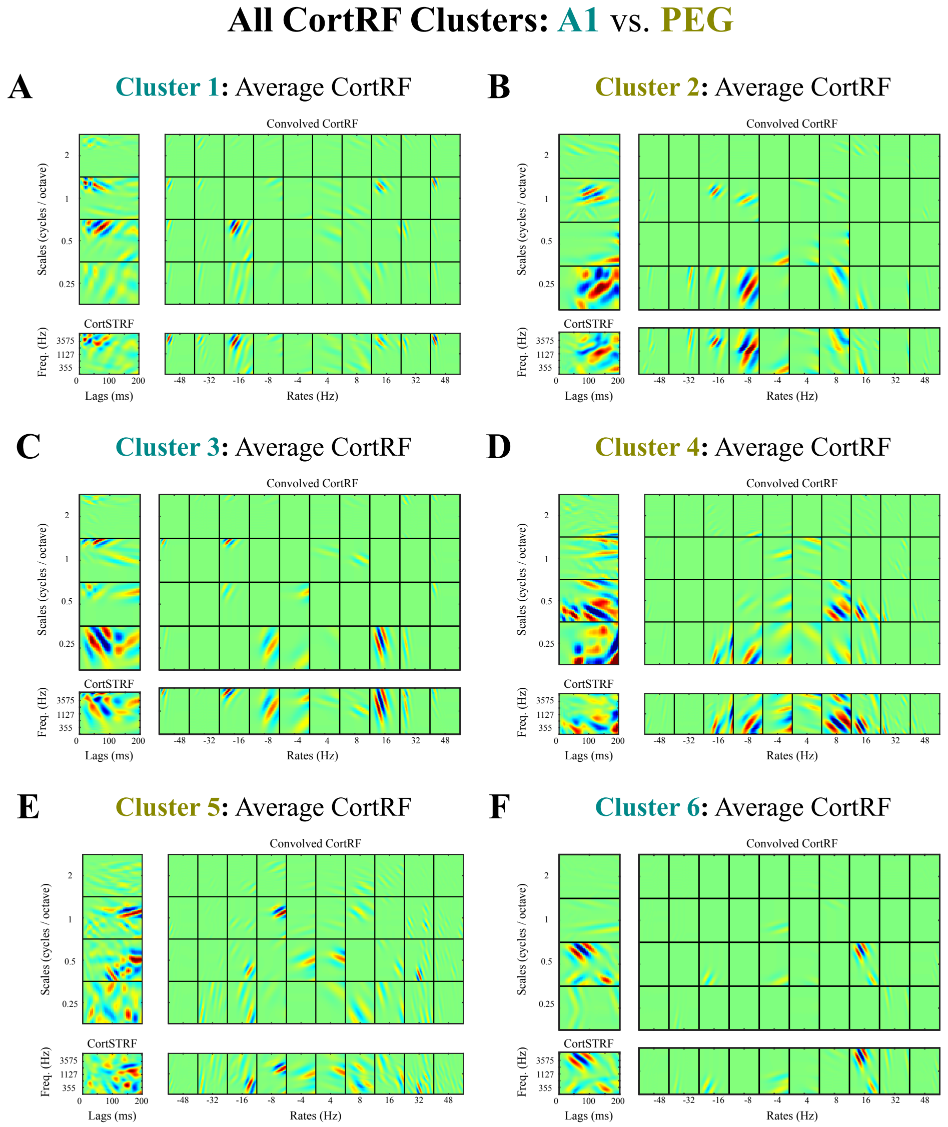

Fig S3. CortRF cluster averages for all six clusters (**A**–**F**). The largest clusters (A, D, and E) are also shown in Fig 8. Each panel shows the average convolved CortRF and its marginalization over rates and scales, both separately and jointly. The latter is equivalent to the average CortSTRF of each cluster.
